# Early enforcement of cell identity by a functional component of the terminally differentiated state

**DOI:** 10.1371/journal.pbio.3001900

**Published:** 2022-12-05

**Authors:** Zahra Bahrami-Nejad, Zhi-Bo Zhang, Stefan Tholen, Sanjeev Sharma, Atefeh Rabiee, Michael L. Zhao, Fredric B. Kraemer, Mary N. Teruel

**Affiliations:** 1 Department of Chemical and Systems Biology, Stanford University, Stanford, California, United States of America; 2 Department of Biochemistry and the Drukier Institute for Children’s Health, Weill Cornell Medical College of Cornell University, New York, New York, United States of America; 3 Department of Medicine/Division of Endocrinology, Stanford University, Stanford, California, United States of America; 4 VA Palo Alto Health Care System, Palo Alto, California, United States of America; 5 Department of Bioengineering, Stanford University School of Medicine, Stanford, California, United States of America; 6 Weill Center for Metabolic Health, Division of Endocrinology, Diabetes & Metabolism, Joan and Sanford I. Weill Department of Medicine, Weill Cornell Medical College of Cornell University, New York, New York, United States of America; 7 Department of Physiology and Pharmacology, Thomas J. Long School of Pharmacy and Health Sciences, University of the Pacific, Stockton, California, United States of America; Institute for Systems Biology, UNITED STATES

## Abstract

How progenitor cells can attain a distinct differentiated cell identity is a challenging problem given the fluctuating signaling environment in which cells exist and that critical transcription factors are often not unique to a differentiation process. Here, we test the hypothesis that a unique differentiated cell identity can result from a core component of the differentiated state doubling up as a signaling protein that also drives differentiation. Using live single-cell imaging in the adipocyte differentiation system, we show that progenitor fat cells (preadipocytes) can only commit to terminally differentiate after up-regulating FABP4, a lipid buffer that is highly enriched in mature adipocytes. Upon induction of adipogenesis in mouse preadipocyte cells, we show that after a long delay, cells first abruptly start to engage a positive feedback between CEBPA and PPARG before then engaging, after a second delay, a positive feedback between FABP4 and PPARG. These sequential positive feedbacks both need to engage in order to drive PPARG levels past the threshold for irreversible differentiation. In the last step before commitment, PPARG transcriptionally increases FABP4 expression while fatty acid-loaded FABP4 increases PPARG activity. Together, our study suggests a control principle for robust cell identity whereby a core component of the differentiated state also promotes differentiation from its own progenitor state.

## Abstract 2

How can progenitor cells attain a distinct differentiated cell identity, given the fluctuating signaling environment and redundancy of critical transcription factors? This study identifies a control principle whereby progenitor cells induced to differentiate can reach the desired distinct differentiated cell fate despite the possibility of many alternative fates en route.

**Highlights**

- Fatty acid-loaded FABP4 increases PPARG expression, thereby turning on PPARG positive feedback loops that further increase PPARG expression. The small molecule rosiglitazone bypasses the need for FABP4.
- FABP4 critically controls the second phase of adipogenesis between activation of the feedback loops and reaching the threshold to differentiate.
- Only a small fraction (approximately 10%) of the FABP4 levels typically attained in mature fat cells is needed to commit cells to the differentiated state, thus providing an explanation for why maintenance of the mature adipocyte state is so robust.

## Introduction

Terminal cell differentiation is fundamental for developing, maintaining, and regenerating tissues in all multicellular organisms and is the process by which specialized cells such as adipocytes (fat cells), osteoblasts, neurons, and muscle cells are generated from progenitor cells [1]. However, how progenitor cells can reach a specific and unique terminally differentiated state is not well understood. In many differentiation processes, the critical transcription factors and signaling elements driving cell fate are not unique to a specific differentiation process [2–5], raising the question how and when cells can be guided to a unique cell fate. Furthermore, single-cell RNA-seq and other single-cell technologies have provided direct evidence that cell-to-cell variability in protein expression and signaling activities can cause differentiating cells to pass through stochastic intermediate states that could misdirect cells to alternative final fates [6–9]. How a differentiating cell avoids becoming lost in these intermediate states and can reliably find its way to the desired specific terminal cell fate is not clear.

Negative feedback has been shown to play a role in regulating the differentiation decision between multiple fates whereby one fate suppresses the programs that drive differentiation of the other fates [10]. However, since negative feedback mostly prevents cells that have chosen one path from differentiating into alternative cell types, additional regulatory mechanisms must exist that selectively drive cells onto a unique path and allow cells to robustly assume and maintain a specific differentiated cell identity. A number of studies focusing on directed and transdifferentiation processes showed that differentiation can proceed by multiple routes and yet converge onto similar transcriptional states [11,12], consistent with the view that terminally differentiated cell states are “attractor basins” in a transcriptional and signaling differentiation landscape. The finding that the same differentiated state could be reached by passing through different intermediate states motivated us to ask the question whether a unique attractor basin requires a unique cell identify factor that would allow for the same terminally differentiated state to be reached from different intermediate states. Specifically, we considered that cells may assume a robust differentiated cell identity by using a type of positive feedback whereby a core component that is uniquely expressed and needed in the differentiated state doubles up as a signaling co-factor that drives an irreversible step in the differentiation process.

Such a self-reinforcing mechanism that promotes robust cell identity (1) would have to control a late step before commitment to ensure that cells select the correct differentiation path and cell identity; and (2) once cells have committed, would have to robustly lock the cell into its differentiated state. To test if such a positive-feedback mechanism exists and how it may reinforce cell identity of a differentiated state, we used the adipocyte differentiation system since it is a well-characterized and experimentally accessible terminal cell differentiation process [13]. We were also intrigued by a previous observation that increased levels of fatty acids can promote differentiation of precursor cells. As a candidate for such a self-reinforcing mechanism for cell identity, the fatty acid-binding protein, FAPB4, is one of the most abundant proteins in adipocytes, where it makes up between 0.5% to 6% of soluble protein [14]. FABP4 is normally expressed at high levels only in adipocytes and is a critical core component of adipocytes that has important cell-internal functions such as binding to hormone sensitive lipase (HSL) and buffering lipid release [15,16]. However, there is also evidence that FABP4 may have an additional role in positively regulating the transition from progenitor cells into mature adipocytes [17–19]. These observations motivated our study here that FABP4 could provide such a self-reinforcement mechanism for unique cell identity. We note that FABP4 can also be released from mature adipocytes and has cell-external roles in different cell types including preadipocytes and other adipocytes where it has been shown to reduce PPARG expression as a pathological and possibly also normal regulatory function [20].

It is well established that the expression of FABP4 is induced by PPARG, the master regulator of adipocyte differentiation [21]. However, whether FABP4 has a role in regulating PPARG activity during the differentiation process has been difficult to resolve since very little FABP4 is expressed early in adipogenesis [15,22]. Also, it is not clear how FABP4 regulates PPARG and if and how FABP4 and PPARG may reinforce each other’s expression during adipogenesis. Finally, if FABP4 is indeed uniquely important for adipogenesis, it is not clear why genetic studies in FABP4 knockout mice failed to show a suppression of adipogenesis [23].

Here, using live single-cell analysis of fluorescently tagged endogenous PPARG and FABP4, we show that FABP4 and PPARG build up only very slowly during the first phase of the adipogenic program until they transition to a second phase marked by engagement of positive feedback between each other. This feedback starts after about 24 hours when the FABP4 level is very low and ends approximately 12 hours later when the levels of PPARG and FABP4 rapidly build up and pass a critical threshold for differentiation. We show that the activation of PPARG by lipid ligands is suppressed when cellular levels of FABP4 are reduced by small interfering RNA (siRNA)-mediated depletion. This suggests that FABP4 has—at much lower levels than seen in differentiated cells—a transport function to enhance fatty acid binding to PPARG, a mechanism which is known to increase PPARG activity [21]. Together, our study provides support for a general model that robust cell identity can be initiated and reinforced by having a unique core component of the differentiated state double up as a signaling factor that initiates and then reinforces the path to this unique differentiated state.

## Results

### FABP4 is needed for adipogenesis in vitro and in vivo

**Fig 1:**
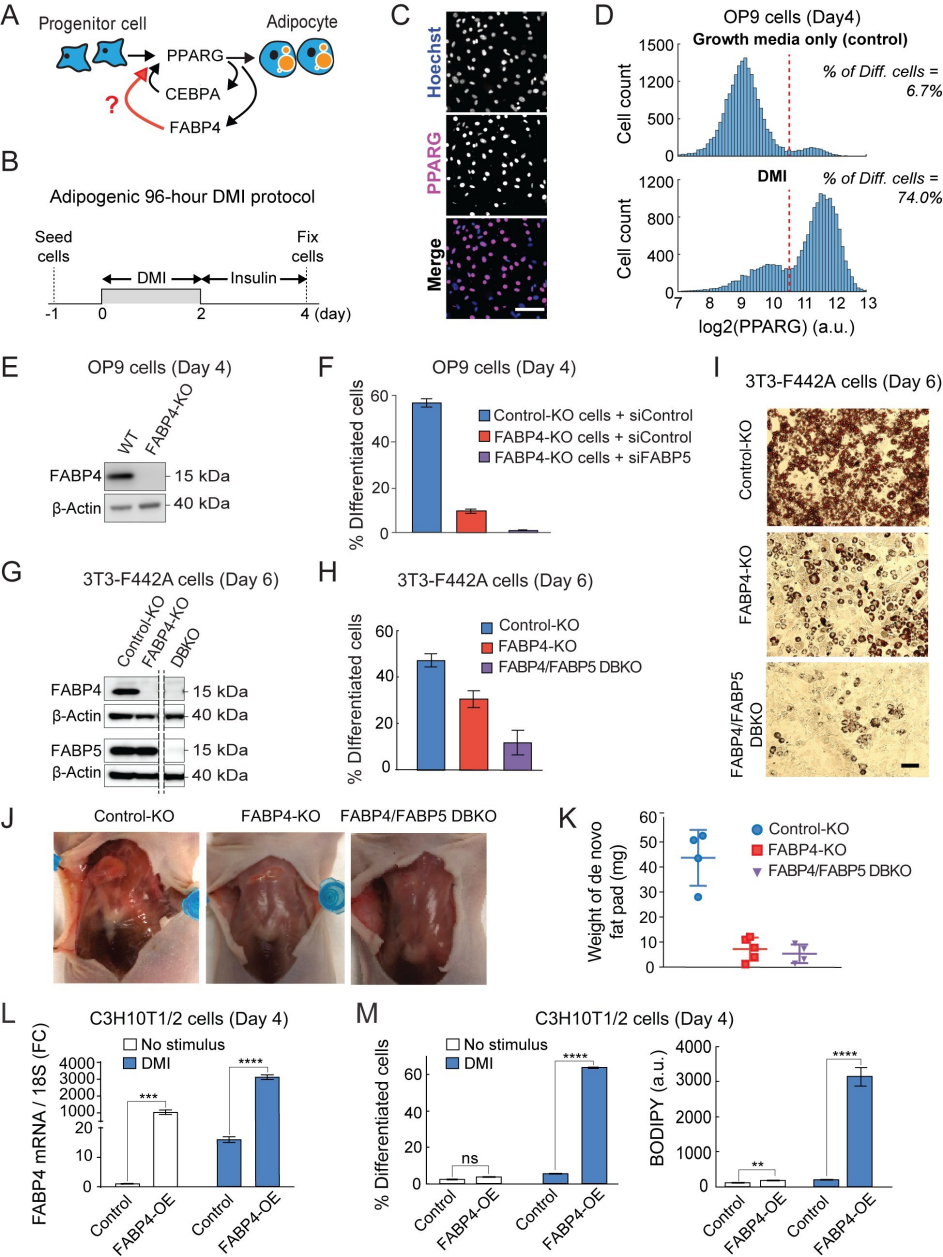
FABP4 is needed to drive adipogenesis in vitro and in vivo. (A) Schematic of proposed cell-identity positive feedback. (B) Schematic of the standard 96-hour DMI protocol used to induce adipogenesis. (C) OP9 preadipocyte cells were induced to differentiate using the 96-hour DMI protocol as shown in (B). Cells were stained with Hoechst to visualize nuclei. Immunocytochemistry was performed to measure PPARG protein levels. Scale bar, 50 μm. (D) Bimodality in single cell abundance of PPARG. The percent of differentiated cells was assessed by counting the number of cells above the PPARG threshold and dividing by the total number of cells as described in [17,27] (see also Methods). (E) FABP4 was knocked out in OP9 preadipocyte cells using CRISPR-mediated genome editing. The cells were induced to differentiate by using the standard 96-hour DMI protocol. The loss of FABP4 protein was validated by western blot analysis. β-actin was used as a loading control. (F) OP9 cells that were transfected and sorted at the same time but that did not harbor FABP4 knockout were used as a control (Control-KO). Control-KO and FABP4-KO OP9 cells transfected with FABP5 or nontargeting siRNA were stimulated with 96-hour DMI protocol. PPARG expression and adipogenesis (% of differentiated cells) were assessed as in (D). Bar plots show mean +/–SEM from 3 technical replicates with approximately 1,000 cells per replicate. Data is representative of 3 independent experiments. See also S1 Fig. (G, H) FABP4 and FABP5 in 3T3-F442A cells were knocked out as in (E). 3T3-F442A cells that were transfected and sorted at the same time but that did not harbor FABP4 or FABP5 knockout were used as a control (Control-KO). Cells were grown to 2 days post-confluence and were induced to differentiate by addition of insulin. Cells were assayed at day 6 post-induction. (G) The loss of FABP4 and FABP5 proteins was validated by western blot analysis. β-actin was used as a loading control. (H) The percent of differentiated cells were assessed as in (D). Bar plots show mean +/–SEM from 3 technical replicates with approximately 1,000 cells per replicate. Data is representative of 3 independent experiments. See also S2 and S3 Figs. (I) The extent of adipogenesis was assessed by Oil Red O staining for intracellular lipid accumulation. Scale bar, 100 μm. (J) 3T3-F442A preadipocytes were subcutaneously injected at the sternum of mice, and photographs were taken 4 weeks after injection. (K) The weight of the fat pads extracted 4 weeks after implantation. Error bars show mean +/− SD. (L, M) C3H10T1/2 MSCs suitable to induce CRISPR-based activation of genes (CRISPRa, see Methods) were transfected with sgRNA targeting the FABP4 promoter (FABP4-OE) in order to induce expression of FABP4, or with an empty vector (Control); 48 hours later, the cells were induced to differentiate by the standard 96-hour DMI protocol as in (B). (L) qRT-PCR was carried out to measure FABP4 expression. Data is normalized to 18s expression. (M) Immunocytochemistry was carried out as in (D) using anti-PPARG antibody to measure the percent of differentiated cells and BODIPY to measure lipid content. (L, M) Bar plots show mean +/–SEM from 3 technical replicates. Data is representative of 3 independent experiments. Unpaired *t* test, *, *p* < 0.05; ***, *p* < 0.001; ****, *p* < 0.0001. See also S4 Fig. The data underlying the graphs in the figure can be found in https://zenodo.org/record/7012787#.Y2I5I0zP3b0. MSC, mesenchymal stem cell; sgRNA, single guide RNA; siRNA, small interfering RNA.

Previous studies suggested that FABP4 and PPARG are in a positive feedback relationship [17,19,22,24] (Fig 1A). One arm of this positive feedback is well established since there are PPARG binding sites on the FABP4 promoter, and PPARG activity has been shown to strongly up-regulate FABP4 mRNA and protein levels [14]. However, the relevance of FABP4 in regulating PPARG has been controversial as FABP4 knockout mice are not defective in adipogenesis [23]. We therefore performed a series of experiments to address if and how FABP4 can regulate PPARG expression and adipogenesis.

Previous studies showed that the process of adipogenesis results in a bimodal distribution of PPARG levels in which cells are either undifferentiated (low PPARG) or differentiated (high PPARG) [17,25]. When mouse OP9 preadipocyte cells are induced to differentiate using a standard 96-hour DMI protocol (Fig 1B), the percent of differentiated cells at any time point during adipogenesis can be assessed by carrying out immunocytochemistry to determine whether PPARG expression is above or below a threshold value (Fig 1C and 1D, see also Methods) [17,26,27]. We first carried out CRISPR-mediated genome editing to completely knock out FABP4 expression in OP9 cells (Figs 1E and S1A). We then induced adipogenesis using the 96-hour DMI protocol and tested how adipogenesis could be affected by measuring the percent of differentiated cells as in Fig 1D.

As expected, control-KO OP9 cells differentiated robustly (Fig 1F). In contrast, FABP4-KO OP9 cells were strongly defective in increasing PPARG expression and differentiating, with only a small fraction of cells differentiating compared to control-KO cells. We suspected that the residual differentiation in FABP4-KO cells could be due to compensation by FABP5. FABP5 is expressed at an approximately 100-fold lower concentration than FABP4 in adipocytes[28], but studies by us and others have shown that FABP5 can be up-regulated to partially compensate for the loss of FABP4 in cells and mice (S1B Fig) [23,29,30]. FABP5 does not bind to PPARG directly, but rather increases PPARG activity and adipogenesis indirectly, possibly through binding to PPARdelta which has been shown to potentiate PPARG activity [31,32]. To test whether FABP5 expression was responsible for the remaining small fraction of differentiated cells in the FABP4-KO cells, we carried siRNA-mediated knockdown of FABP5 and indeed adipogenesis was reduced to very low levels (Figs 1F and S1C and S1D).

We further validated these findings in another commonly used preadipocyte cell system, 3T3-F442A cells [33]. We used CRISPR-mediated genome editing to knockout FABP4 or both FABP4 and FABP5 (Figs 1G and S2A and S2B and S3). 3T3-F442A cells that had been subjected to the same FABP4 knockout protocol, but which did not harbor any FABP4 knockout were used as control cells. We plated the 3T3-F442A cells into 96-well plates and induced adipogenesis using the standard protocol in 3T3-F442A cells which is to add insulin [33]. Indeed, when FABP4 was knocked out, 3T3-F442A preadipocytes showed reduced differentiation and reduced lipid accumulation, as measured by PPARG staining and Oil Red O staining, respectively (Figs 1H and 1I and S2C). A lack of lipid accumulation is indicative that most of the KO cells are not functional mature adipocytes. As in the case of the OP9 preadipocyte cells (Fig 1F), some differentiation was observed in the FABP4-KO cells, but this differentiation was abolished when FABP5 was also knocked out (Fig 1H and 1I), supporting that FABP5 can compensate for a lack of FABP4.

To test whether FABP4 is essential in vivo for adipogenesis, we used a previously established method in which 3T3-F442A preadipocytes are subcutaneously injected into the sternum of immune-deficient mice, giving rise to fat pads resembling normal adipose tissue [34]. Since fat is not normally present at the sternum of mice, the fat pad formed at the sternum after injection of preadipocyte cells is generated by de novo adipogenesis of the injected cells [34]. We injected our preadipocyte cells into the sternum of 8-week mice, and a fat pad was allowed to form for 4 weeks. The FABP4-KO and FABP4/FABP5 double knockout (DBKO) preadipocyte cells indeed showed a defect in adipogenesis, as measured by weighing the fat that formed at the sternum (Fig 1J and 1K).

Our results so far show that a lack of FABP4 reduces PPARG expression and adipogenesis. To further establish a positive feedback loop from FABP4 to PPARG, we carried out experiments to determine how FABP4 overexpression affected PPARG levels. To overexpress FABP4, we used CRISPR-mediated activation of FABP4 expression (CRISPRa) in C3H10T1/2 cells, a well-established mesenchymal stem cell (MSC) model that is capable of differentiating into different cell fates, including adipocytes [35]. The C3H10T1/2 cells were stably transfected with dCas9-VP64 and MS2-P65, the 2 core components of the CRISPRa SAM system, a second generation CRISPR-mediated activation system [36]. These C3H10T1/2-CRISPRa-SAM cells allowed us to potently activate specific genes by transfecting the cells with targeted single guide RNA (sgRNA). To induce expression of FABP4, we transfected these cells with sgRNA targeting the promoter regions of FABP4. Two days after inducing FABP4 expression, we applied a DMI differentiation stimulus to the cells. Inducing differentiation by addition of DMI stimulus is normally not strong enough to induce adipogenesis in CH310T1/2 MSC cells. However, overexpression of FABP4 resulted in a robust increase in PPARG and adiponectin mRNA (Figs 1L and S4A), as well as in robust expression of PPARG protein and lipid accumulation (Figs 1M and S4B), compared to control cells. Since adiponectin expression and high lipid accumulation are markers of mature adipocytes, our results argue that FABP4 expression can lead to a robust increase in PPARG expression and can drive conversion of progenitor cells into mature adipocytes.

### Live-cell imaging of endogenous PPARG and FABP4 expression shows a small increase in FABP4 levels before cells reach the threshold for differentiation

**Fig 2:**
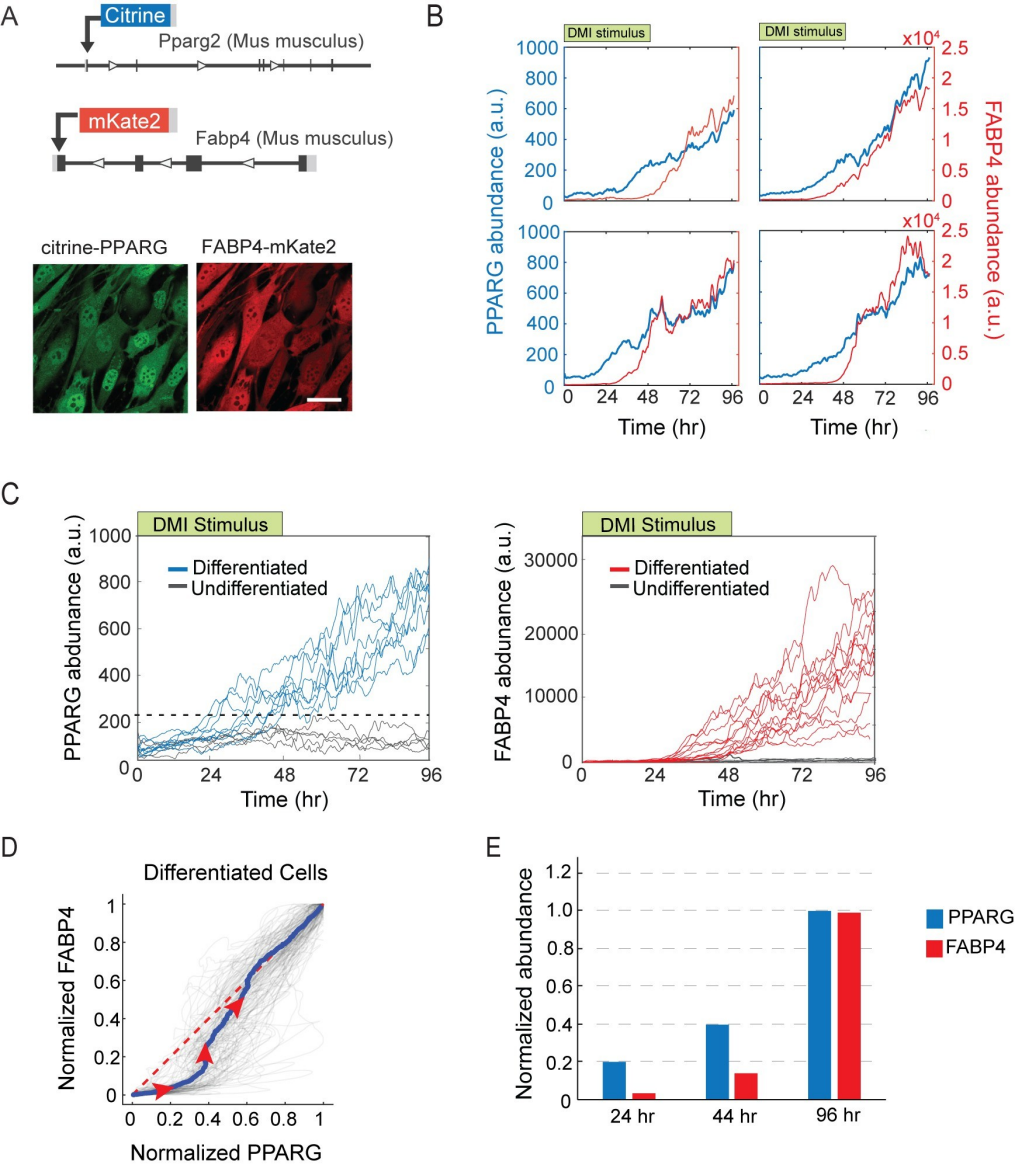
There is a delayed, small increase in FABP4 early in adipogenesis. (A) Endogenous FABP4 was tagged with mKate2 (RFP) in the citrine-PPARG cells. Images show live dual-tagged OP9 cells differentiated for 4 days into adipocytes by applying the standard DMI protocol as in Fig 1B. Scale bar, 10 μm. See S5 and S6 Figs for description of tagging protocol. (B) Changes in citrine-PPARG and FABP4-mKate2 expression measured in the same cell over the time course of adipogenesis. Time courses from 4 representative cells are shown. (C) Analysis of citrine-PPARG and FABP4-mKate2 time courses in a population of differentiating cells show that there is a threshold level at which cells commit to irreversibly differentiate. The dashed black line represents the PPARG threshold level. (D) For each eventually differentiated cells in (C), PPARG and FABP4 time courses were normalized to its minimum and maximum values so that all cells will start from point (0,0) and end at point (1,1). Gray lines are 100 representative cells. The blue line is the mean of about 600 cells. The red dashed line marks the diagonal. Red arrows indicate different directions on the 2D space. (E) Bar plots show the increase in PPARG and FABP4 relative to the maximal levels of the respective proteins reached during the time course in (C). Representative of 3 independent experiments. The data underlying the graphs in the figure can be found in https://zenodo.org/record/7012787#.Y2I5I0zP3b0.

Our in vitro and in vivo results in Fig 1, together with previous in vitro work by us and others [19,22,24], support that PPARG and FABP4 are in a positive feedback relationship: PPARG not only increases FABP4 expression, but also FABP4 can increase PPARG expression and adipocyte differentiation. To now understand if, when, and how a FABP4-PPARG feedback relationship functions during adipogenesis, we designed live-cell imaging experiments that allow us to precisely determine when FABP4 and PPARG increase relative to each other in the same single cells. We used CRISPR-mediated genome editing to tag endogenous FABP4 and PPARG in mouse OP9 preadipocyte cells with orthogonal fluorescent proteins (Figs 2A and S5 and S6). Using these dual-tagged citrine(YFP)-PPARG and FABP4-mKate2(RFP) cells, we carried out time-course analysis in thousands of individual cells stimulated to undergo adipogenesis using the standard 4-day DMI protocol (Fig 1B) [17]. Time courses of PPARG and FABP4 abundance in representative cells displayed significant cell-to-cell variability (Fig 2B). However, PPARG and FABP4 levels consistently showed an overall similar dynamic to each other: Levels of both proteins increased very slowly for the first 24 hours before increasing more rapidly from 24 to 48 hours. Furthermore, even after the adipogenic stimulus was removed after 48 hours, the levels of both PPARG and FABP4 protein continued to further increase for days.

In examining the PPARG time courses in the cell population in response to the DMI stimulus, a threshold level in PPARG is apparent (dashed black line in Fig 2C, left), as has been previously described [17,26,27]. If PPARG levels in a cell reach this threshold level, the cell will go on to differentiate, even if the adipogenic stimulus is removed at 48 hours [17,26,27]. However, if PPARG levels in the cell do not reach this threshold level, PPARG levels in that cell will stay low or drop back down, and the cell will remain undifferentiated. Our analysis showed that cells that go on to differentiate according to their PPARG levels (blue traces in Fig 2C, left) already had increased their FABP4 levels before the threshold was reached (red traces in Fig 2C, right). The cells then increased their FABP4 levels even more strongly after reaching the threshold. Correspondingly, cells that remained undifferentiated according to their PPARG levels also did not increase their FABP4 levels (gray traces in Fig 2C).

Interestingly, even though PPARG and FABP4 protein expression dynamics look similar, the relative increase in the levels of PPARG and FABP4 is significantly different during adipogenesis. This is first indicated in the plots in Fig 2B in which the expression of FABP4 is slightly delayed relative to PPARG expression for each representative single cell. To exclude the possibility that the observed delay is just a unique property of the 4 selected cells, we normalized PPARG and FABP4 expression of about 600 single cells and plotted the time courses in a 2D space such that all the cell time courses start from the bottom left corner (0,0) and end at the top right corner (1,1) (Fig 2D). For cells that eventually differentiated, most of their traces first followed the PPARG axis and then turned to follow the FABP4 axis, indicating that PPARG does indeed increase earlier compared to FABP4 in cells that differentiate (Fig 2D).

We next normalized and plotted the median level of PPARG and FABP4 in the time courses from the Fig 2C experiment. Over the 96-hour DMI differentiation time course, PPARG levels increased only approximately 8-fold while FABP4 levels increased approximately 100- to 2,000-fold, as observed in individual cells that differentiated (Fig 2C). Furthermore, in cells that eventually went on to differentiate, PPARG levels increased steadily over the time course of adipogenesis, by approximately 10% of maximal after 24 hours and approximately 50% of maximal after 44 hours. In striking contrast, the increase in FABP4 was markedly suppressed for the first 44 hours (Fig 2E). FABP4 abundance increased to only a few percent of maximal in the first 24 hours and then to only 10% of maximal by 44 hours before becoming dramatically up-regulated late in adipogenesis (Fig 2E).

The small early increase in FABP4 abundance before cells reach the threshold is difficult to see without single-cell time course analysis. Previous studies using bulk-cell approaches such as western blots to quantify FABP4 expression lacked the sensitivity to observe the small early FABP4 increase that occurs only in the subset of cells that differentiate, which is likely the reason why it has been commonly thought that FABP4 is only downstream of PPARG and is induced late in adipogenesis after the critical signaling events for differentiation have already happened [37]. Taken together, our live, single-cell imaging measurements, as well as previous mass spectrometry measurements [22], show that there is a small but significant increase in FABP4 levels that occurs after a long delay from when the adipogenesis program is initiated, but before cells reach the PPARG threshold at which cells commit to terminally differentiate.

### The small increase in FABP4 protein levels occurs during an intermediate step in adipogenesis and is needed to push PPARG levels up to the threshold to differentiate

**Fig 3:**
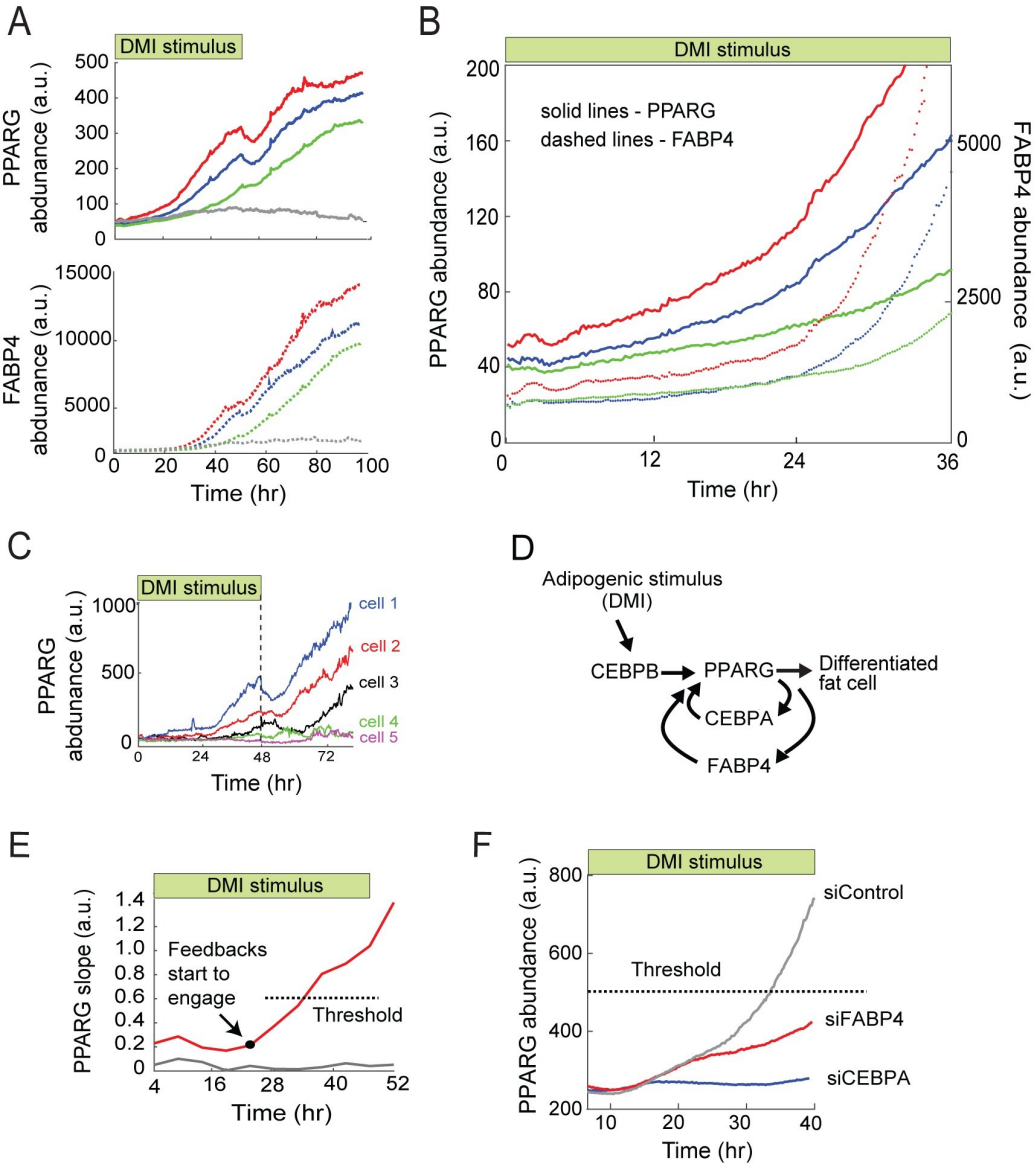
The early, small increase in FABP4 is needed to push PPARG levels to the threshold to differentiate. (A) Time courses of 494 dual-tagged cells were binned according to their PPARG level at 48 hours, and the traces in each bin were averaged. Plot shows representative averaged time courses to show a range of differentiation outcomes. The data shown is representative of 5 independent experiments. (B) Zoomed-in region of (A) showing a direct comparison of the gradual increases in PPARG and FABP4 before both get self-amplified when cells reach the engagement point. (C) Examples of single-cell time courses of PPARG expression in citrine-PPARG OP9 cells stimulated to differentiation using the standard 96-hour DMI protocol. (D) Schematic of the relationship between CEBPB, CEBPA, FABP4, and PPARG to control adipogenesis. (E) Red and gray lines show the average slope of approximately 700 PPARG time courses of eventually differentiated cells and of approximately 300 PPARG time courses of eventually undifferentiated cells, respectively, in a typical DMI-induced differentiation experiment. The slope of PPARG at each time point was calculated by using a linear fit to 8-hour segments of the PPARG abundance trajectory (+/- 4 hours). The time point when the feedbacks start to engage is reflected in a change in PPARG slope and occurs many hours before a cell will cross the PPARG threshold level at which differentiation is irreversibly triggered. Data is representative of 5 independent experiments. (F) Citrine-PPARG OP9 cells transfected with siRNA targeting CEBPA, FABP4, or scrambled control and stimulated to differentiate using the standard 96-hour DMI protocol. Plotted lines are population median traces of approximately 1,000 cells per condition, representative of 6 independent experiments. The data underlying the graphs in the figure can be found in https://zenodo.org/record/7012787#.Y2I5I0zP3b0. siRNA, small interfering RNA.

When inspecting individual time courses of FABP4 and PPARG expression in individual cells, we observed that those cells that increase PPARG early also increase FABP4 early and cells that increase PPARG at a later time also increase FABP4 later. This correlation can best be seen by dividing time courses from a typical differentiation experiment into 4 bins and comparing how the average PPARG and FABP4 levels in the corresponding bins change (Fig 3A). Indeed, cells that increase PPARG early (red), near the median (blue), or late (green), as well as cells that do not differentiate (gray), have corresponding changes in FABP4 levels. When focusing on the moment when PPARG levels start to increase (Fig 3B), it is apparent that there is a rapid change in the slopes of both the PPARG and FABP4 time courses between 18 and 36 hours after adipogenic stimulus is added. This change in the rate of increase of PPARG levels is apparent even in individual PPARG traces (Fig 3C). Such a delayed and abrupt change in slope is a hallmark of positive feedback that may involve both FABP4 and PPARG, as well as other established positive feedback partners of PPARG such as CEBPA [22,38], enhancing the activity and expression of PPARG (Fig 3D). We thus refer to this distinct change in the rate of PPARG expression between approximately 18 to 36 hours, which can perhaps best be seen in a slope analysis (Fig 3E), as the time point when the positive feedbacks to PPARG start to engage. We had previously observed that the feedback engagement point occurs before cells reach the threshold to differentiate [17]. To validate this here, we carried out siRNA experiments. Indeed, knockdown of the 2 PPARG feedback partners, CEBPA and FABP4, resulted in a failure to reach the PPARG threshold (Fig 3F). Interestingly, we found that the CEBPA feedback engages before the FABP4 feedback (Fig 3F).

**Fig 4:**
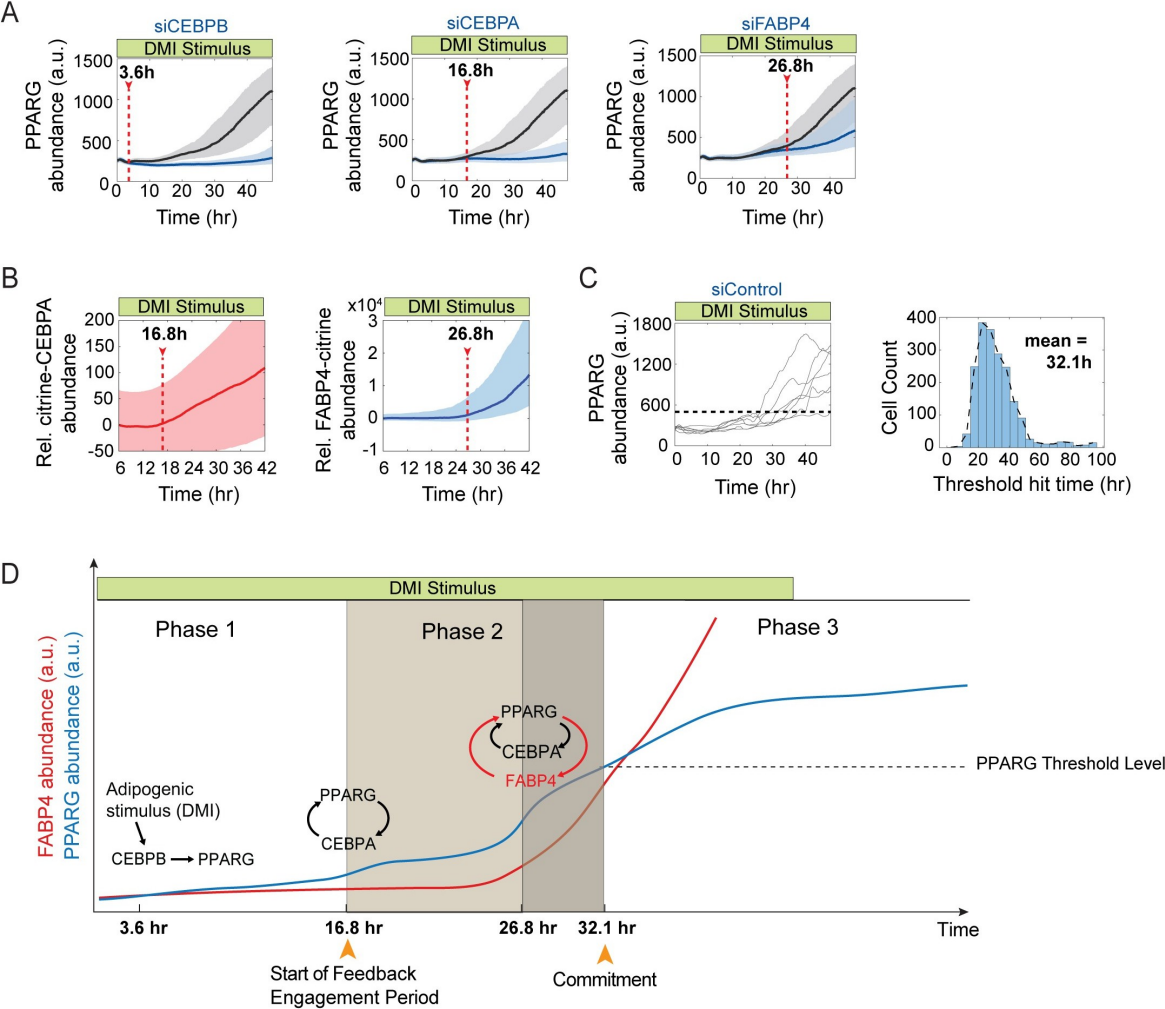
During adipogenesis, the PPARG-FABP4 feedback engages after the PPARG-CEBPA feedback engages. (A) Plots show time courses in citrine-PPARG OP9 cells transfected with siRNA targeting CEBPB, CEBPA, FABP4 (blue lines and shades) or scrambled control (gray lines and shades) and stimulated to differentiate using the standard 96-hour DMI protocol. Plotted lines are population median traces with shaded regions representing 25th and 75th percentiles of approximately 700 cells per condition, representative of 6 independent experiments. For each knockdown time course, the time point at which the targeted protein becomes engaged in adipogenesis is estimated by determining when the traces of knockdown cells start to diverge from that of control cells (see details in S6 Fig). (B) citrine-CEBPA (left) and FABP4-citrine (right) OP9 cells were induced to differentiate by DMI, respectively. Plots are normalized to CEBPA and FABP4 abundance in non-stimulated cells. Bold lines are population median traces with shaded regions representing 25th and 75th percentiles of approximately 3,000 cells per condition. The time points at which CEBPA or FABP4 gets engaged from (A) were marked. (C) Random examples of single-cell time courses of PPARG expression in scrambled control cells from (A) show that the timing to reach PPARG threshold varied across the population (left panel). However, the histogram of the threshold hit times from single cells is strongly unimodel with a mean value of 32.1 hour (right panel). (D) Schematic illustrating the 3 phases of adipogenesis. FABP4 is not active early and only starts to become active later in differentiation to control the critical middle phase of differentiation (Phase 2) during which cells commit irreversibly to differentiate. During Phase 1, PPARG slowly increases to the feedback engagement point, primarily driven by the external DMI stimulus. During Phase 2, both external stimulation and internal self-amplification are needed to increase PPARG levels up to the threshold. In Phase 3, which begins after the threshold is reached, the cell trajectory becomes independent of input stimulus, and a further increase can be driven by internal self-amplification alone until the terminal differentiation state is reached. The observed drop in PPARG level is due to loss of the input stimulus before PPARG levels once again increase due to positive feedback to PPARG. The data underlying the graphs in the figure can be found in https://zenodo.org/record/7012787#.Y2I5I0zP3b0. siRNA, small interfering RNA.

To better determine when the different regulators of adipogenesis become important to drive the terminal differentiation process, we carried out more in-depth analysis of how these proteins influence PPARG increases during adipogenesis. CEBPB is a CEBPA homolog that is believed to function early in adipogenesis (Fig 3D) [39]. We thus considered that CEBPB may be required first and that the 2 positive feedbacks centered on FABP4 and CEBPA may then act either in parallel or sequentially during the feedback engagement point following CEBPB activation (Fig 3D) [17]. Indeed, as shown in Fig 4A, siRNA-mediated depletion of CEBPB suppresses the initial gradual PPARG increase already at approximately 4 hours after DMI addition, supporting a critical role of CEBPB in mediating the initial slow gradual increase in PPARG that occurs before the positive feedbacks engage. In contrast, siRNA-mediated depletion of CEBPA suppresses the normal PPARG increase only at approximately 17 hours after differentiation is induced (Figs 3F and 4A). Strikingly, siRNA-mediated depletion of FABP4 suppresses the normal PPARG increase much later, at approximately 27 hours after stimulation (Figs 3F and 4A and S7), suggesting that CEBPA precedes FABP4 in driving PPARG expression.

To further validate the order of feedback loop engagement using an orthogonal method, we directly measured the expression of CEBPA and FABP4 by using 2 OP9 cell lines in which either endogenous CEBPA or endogenous FABP4 had been tagged with citrine(YFP) using CRISPR-mediated genome editing [26]. The times when the abundances of citrine-CEBPA and FABP4-citrine start to significantly increase from basal levels (Fig 4B) coincides with the times when the CEBPA-PPARG and FABP4-PPARG feedbacks respectively engage (Fig 4A). Taken together, these results argue that the CEBPA-PPARG and FABP4-PPARG feedback loops act sequentially to drive the increase in PPARG in an intermediate phase of the differentiation process before the threshold is reached at approximately 32 hours after DMI stimulation (Fig 4C).

Our results are consistent with the schematic shown in Fig 4D: CEBPB drives the initial slow gradual increase in PPARG during an initial Phase 1, which is then followed by an intermediate Phase 2 of adipogenesis when a first positive feedback between PPARG and CEBPA engages. After a delay, this first positive feedback is further amplified by a second positive feedback between PPARG and FABP4 that engages just before cells reach the threshold for differentiation. Once cells reach the PPARG threshold, high PPARG levels become self-sustaining, being driven by positive feedback independently of input stimulus. In this maintenance phase (Phase 3), the positive feedbacks stay active and cause further increases in PPARG levels, perhaps to lock cells more strongly in the differentiated state. In this Phase 3 of the differentiation process, FABP4 levels also dramatically increase too much higher levels as shown in the time courses in Fig 3.

The PPARG threshold, which marks the transition between Phase 2 and Phase 3, cannot be seen just by inspecting the single-cell or averaged traces. The threshold needs to be identified either (1) by using live-cell imaging to measure PPARG levels, removing the external differentiation stimulus, and then continuing to track each individual cell to know their final differentiation state days later [17,27]; or (2) by taking advantage of the binary nature of the differentiation decision and carrying out suitable analyses such as a Gaussian mixture model analysis [27] or a receiver operating characteristic (ROC) analysis in which different threshold levels are surveyed to find the one that maximizes the difference between true and false-positive rates for predicting cell fate choice [26,40]. Taken together, our results support that the intermediate phase of adipogenesis, Phase 2, is driven by engagement of first CEBPA and then FABP4 to trigger sequential positive feedbacks that drive PPARG to the threshold necessary for cells to become irreversibly differentiated.

### FABP4 is needed to transport lipid ligand to PPARG in order for preadipocytes to differentiate

**Fig 5:**
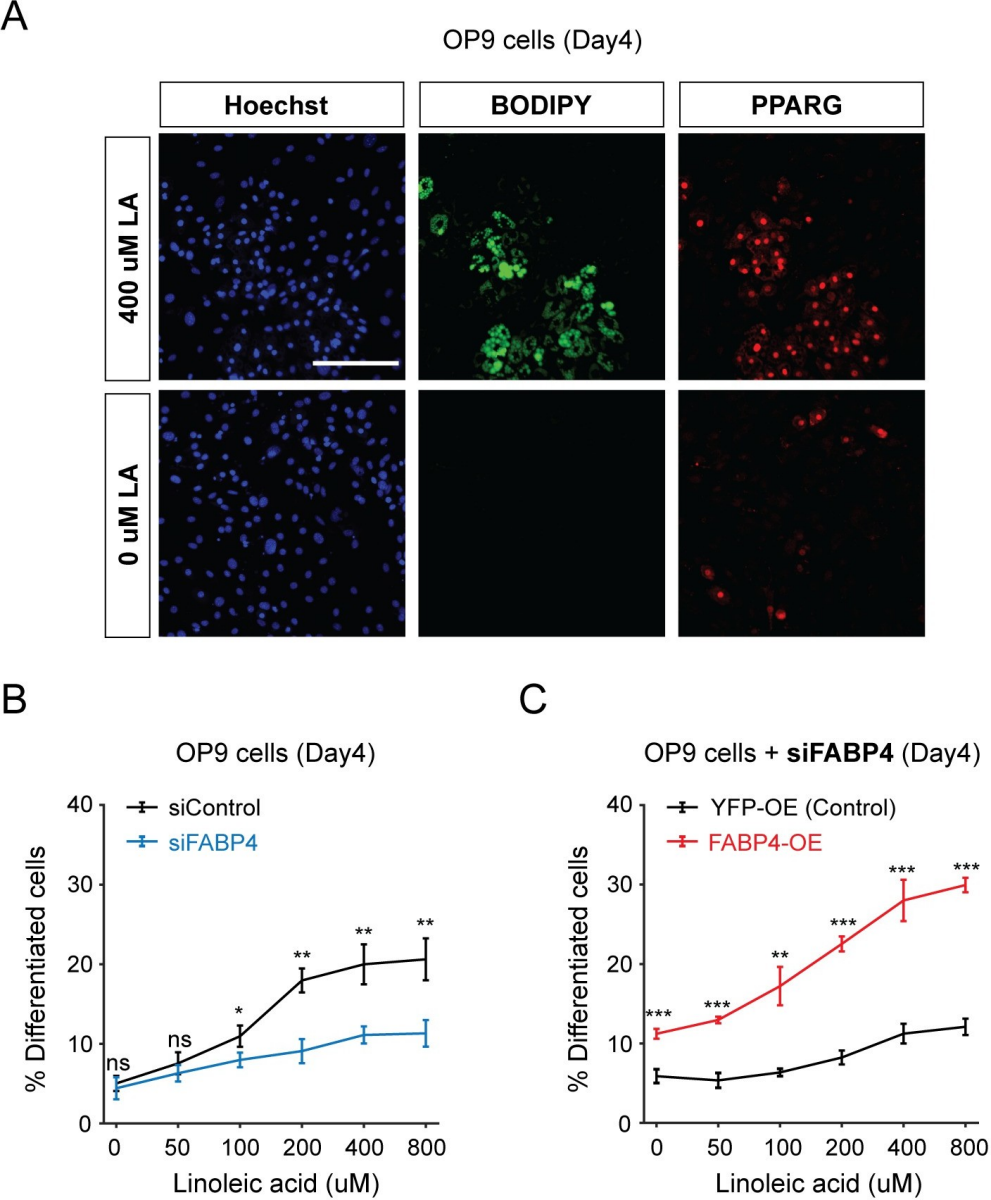
The lipid-binding activity of FABP4 is needed to increase PPARG expression and induce adipogenesis. (A) Linoleic acid (LA) can induce adipogenesis in OP9 cells. Approximately 400 μm LA were added to OP9 cells at day 0 and refreshed every 2 days. Cells were fixed at day 4 and stained with Hoechst to visualize nuclei, BODIPY to visualize lipids and antibody against PPARG. LA-treated cells accumulated more lipids and PPARG protein than the ones treated with only growth media. Scale bar, 100 μm. (B) OP9 cells transfected with FABP4 or control siRNA were stimulated to differentiate by different doses of LA for 4 days. (C) OP9 cells transfected with FABP4 siRNA along with CMV-FABP4 (FABP4-OE) or CMV-YFP (YFP-OE, control) construct were stimulated to differentiate by different doses of LA for 4 days. (B, C) The percentage of differentiated cells was assessed by counting the cells with PPARG levels above the threshold divided by the total number of cells. Bar plots represent mean +/- SD from 3 technical replicates with approximately 6,000 cells per replicate. The significance of the difference between conditions was assessed with unpaired *t* tests (ns, not significant; *p < 0.05; **p < 0.01; ***p < 0.001). Data is representative of 3 independent experiments. The data underlying the graphs in the figure can be found in https://zenodo.org/record/7012787#.Y2I5I0zP3b0. LA, linoleic acid; siRNA, small interfering RNA.

PPARG is a nuclear receptor that is activated by lipid ligands [21]. Previous studies proposed that FABP4 may have a role to deliver fatty acid ligands from the cytosol to PPARG in the nucleus [18,19,24], thereby enhancing the transcriptional activity of PPARG and ultimately promoting adipogenesis. Since such a role of FABP4 in increasing transcriptional activity of PPARG activity has been controversial, we setup an assay to test for such a role of FABP4 during adipogenesis. We first confirmed that exogeneous fatty acids can cause differentiation of fat cells. We incubated OP9 cells for 4 days with linoleic acid, a naturally occurring PPARG fatty acid ligand [41]. We then fixed the cells and carried out immunocytochemistry in which we stained with PPARG to assess the degree of adipogenesis and BODIPY to measure lipid accumulation. As shown in Fig 5A, linoleic acid-treated cells accumulated significant more lipid droplets and PPARG proteins than the ones treated with only control media, indicating that linoleic acid stimulation can drive OP9 preadipocytes to differentiate into mature fat cells. We then carried out siRNA-mediated depletion of FABP4 and induced adipogenesis by adding linoleic acid. We assessed the percent of differentiated cells as in Fig 1D and found that differentiation was significantly reduced across a wide range of linoleic acid concentrations (Fig 5B). Furthermore, overexpressing a siRNA-resistant version of FABP4 rescued the reduction in differentiation caused by FABP4 knockdown (Fig 5C). Since PPARG needs fatty acids to be activated and cause adipogenesis [21], our results in Fig 5B and 5C support that FABP4 is essential for adipogenesis since the activation of PPARG by fatty acid ligands requires FABP4 expression.

### Rosiglitazone is a small molecule, high-affinity activator of PPARG that can bypass the need for FABP4 to transport lipid to PPARG

**Fig 6:**
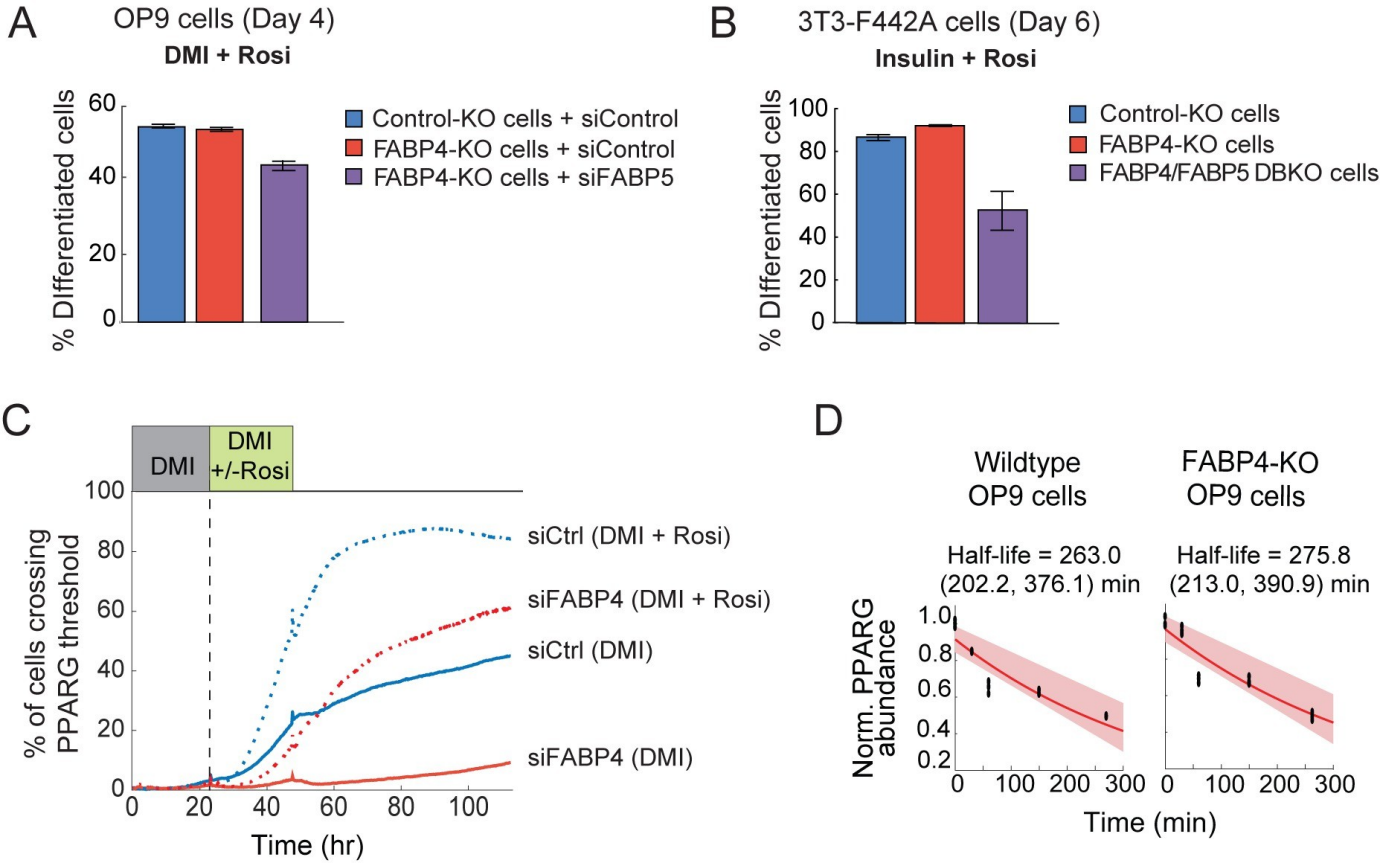
Rosiglitazone, a PPARG agonist that binds to the same binding pocket in PPARG as fatty acid, bypasses the need for FABP4 to up-regulate PPARG expression during adipogenesis. (A) Addition of rosiglitazone (1 μm) to the differentiation stimulus (DMI) rescues the loss of adipogenesis shown in Fig 1F in FABP4-KO OP9 cells and in FABP4-KO OP9 cells transfected with FABP5 siRNA. The percent of differentiated cells was quantitated as in Fig 1D. See also S1D and S1E Fig. (B) The addition of rosiglitazone (1 μm) to the differentiation media (insulin added to the growth media) rescues the loss of adipogenesis in FABP4-KO, FABP5-KO, and DBKO 3T3-F442A cells shown in Fig 1H. See also S2C and S2D and S3 Figs. (C) Citrine-PPARG cells were transfected with FABP4-targeted or control siRNA and were imaged while being induced to differentiate by the standard DMI protocol with and without rosiglitazone added to the DMI stimulus. Knockdown of FABP4 results in loss of differentiation. However, this phenotype was partially rescued by addition of rosiglitazone. (D) OP9 cell lines were stimulated with linoleic acid for 48 hours to induce interaction between FABP4 and PPARG, and then cyclohexamide was added. The cells were fixed at the indicated time points, and immunocytochemistry was performed to measure PPARG protein levels. Three technical replicates for each condition are shown (black dots). The shaded region represents the 95th confidence bounds on the fitted coefficients. Values enclosed in parentheses show lower and upper bounds on the estimated half-life derived from the 95% confidence bounds. The data underlying the graphs in the figure can be found in https://zenodo.org/record/7012787#.Y2I5I0zP3b0. siRNA, small interfering RNA.

Our results thus far support that FABP4, or FABP5 in an indirect compensatory role [19,31], is needed to increase PPARG expression to high enough levels to switch progenitor cells into adipocytes. However, there is a puzzling finding in the literature that mouse embryonic fibroblasts (MEFs) from FABP4 and FABP5 DBKO mice can still differentiate into adipocytes [42]. We tested whether the contradiction could be due to the fact that rosiglitazone had been added to the standard cocktail used to induce adipogenesis. Rosiglitazone is a small molecule, high-affinity activator of PPARG that binds to the same binding pocket of PPARG as fatty acids [43]. We were indeed able to reproduce this result and could induce FABP4-KO and FABP4/FABP5-KO preadipocytes to differentiate by also adding rosiglitazone to the adipogenic cocktail. We had previously shown that knockout of FABP4 alone, or together with knockdown or knockout of FABP5, resulted in a dramatic reduction in PPARG expression and differentiation in response to an adipogenic stimulus (Fig 1F and 1H). However, when we added rosiglitazone along with the adipogenic stimulus to FABP4-KO OP9 and 3T3-F442A cells, the differentiation capacity of the cells was restored (Fig 6A and 6B compared to Fig 1F and 1H). Approximately 50% of the differentiation capacity was restored in the DBKO 3T3-F442A cells, and an even higher fraction of differentiation was restored for the DBKO OP9 cells (Fig 6A and 6B compared to Fig 1F and 1H).

A live-cell analysis of rosiglitazone addition in cells with endogenously tagged PPARG provides mechanistic insights how rosiglitazone acts (Figs 6C and S8). In DMI-stimulated control cells, addition of rosiglitazone had little effect early in adipogenesis but then caused a much more rapid increase in PPARG levels when the feedback engagement point is reached after 24 to 36 hours. For the same conditions with and without rosiglitazone, siRNA-mediated depletion of FABP4 suppressed the rapid increase in control cells. However, rosiglitazone partially restored the fast increase of PPARG in FABP4 knockdown cells. Our results can be interpreted that rosiglitazone, which is soluble in water at low concentrations and has a very high affinity for PPARG [43], can directly access the activating binding pocket of PPARG without needing a cell-internal lipid transport mechanism provided by FABP4. In contrast, fatty acids, which have a much lower solubility in water, require a lipid transport mechanism to reach PPARG and this transport function requires FABP4 in these cell systems.

An alternative regulatory mechanism is that FABP4, which is not a transcription factor, may increase the level of PPARG protein. Such a mechanism is possible since FABP4 can bind to PPARG [19,44] in the presence of lipid, and it is conceivable that FABP4 may increase PPARG expression indirectly by stabilizing PPARG proteins. To test for such a potential stabilization, we measured the degradation rate of PPARG in a condition in which lipid-loaded FABP4 was present to interact with PPARG. We added linoleic acid to wild-type OP9 preadipocyte cells that expressed FABP4 versus to OP9 cells in which FABP4 had been knocked out (FABP4-KO cells from Fig 1E). However, as shown in Fig 6D, we found no significant difference in PPARG degradation rate, suggesting that FABP4 does not stabilize PPARG. Together, these results provide support for the model that lipid-loaded FABP4 increases PPARG expression, not by stabilizing PPARG, but rather indirectly by bringing lipid to PPARG to increase PPARG activity [19,24,44] which then drives PPARG to increase its expression through its transcriptional feedback partners such as CEBPA or also through autoregulation [25,38].

## Discussion

Our study focuses on a general question how cells ensure that they assume and maintain a specific terminally differentiated state given that differentiating cells typically have multiple cell-fate options, and there is also cellular plasticity with cells being able to transition between different reversible progenitor states. Our study considered that one solution to the cell identity problem is to reinforce the differentiation path to a specific differentiated state by using a unique core component of the differentiated state also in a separate regulatory capacity to both control a key step in the differentiation process and to then maintain the differentiated state. Here, we tested whether FABP4, one of the most abundant proteins in mature adipocytes that regulates lipid flux, could function as such a cell identity factor in adipogenesis.

Our rationale for focusing on FABP4 was previous evidence that FABP4 may regulate PPARG expression and/or activity [17,19,22,24] and that FABP4 is highly expressed specifically in adipocytes [16]. We investigated the role of FABP4 by using simultaneous single-cell analysis of endogenously tagged FABP4 and PPARG to test if and when FABP4 and PPARG regulate each other. We used this type of analysis in order to reconcile the seemingly conflicting results in the literature that we thought might be tied to a high cell-to-cell variability in the dynamics of FABP4 expression and the possibility that low levels of FABP4 might be sufficient to activate PPARG. By tracking cells and monitoring expression of PPARG in individual cells over the time course of DMI-induced adipogenesis, we showed that PPARG expression during adipogenesis can be divided into 3 sequential phases with DMI mediating a linear increase in PPARG in Phase 1, positive feedback starting to engage in Phase 2 and ending with PPARG levels reaching a critical threshold where self-amplification takes over and the external input stimulus is not needed any more. Markedly, we showed that this Phase 2 is started by a first positive feedback between PPARG and CEBPA and is about 8 hours later amplified by a second positive feedback between PPARG and FABP4. In Phase 3, this same positive feedback between FABP4 and PPARG helps maintain the differentiated adipocyte state. Only low levels of FABP4 are needed for this role of FABP4 in increasing PPARG activity that ensures first induction and then robust maintenance of the terminally differentiated state.

FABP4 has many essential functions in mature adipocytes, and thus FABP4 would not be a good control signal if a large proportion of it was needed also as a feedback signal. FABP4 and PPARG levels can vary in differentiated cells, but since only a small fraction of the maximal FABP4 levels (<10%) is needed to feedback to PPARG, the differentiated state can be robustly maintained even when there is a 90% drop in FAPB4 level in cells. Such a robust control mechanism is likely useful for cells in vivo to keep cells differentiated once they commit to the terminally differentiated state.

We showed that FABP4 is a necessary co-factor both in OP9 and 3T3-F442A preadipocyte cells to accelerate the buildup of PPARG in Phase 2 to the threshold where differentiation is triggered. It is possible that a similar FABP4-mediated buildup of PPARG also exists in macrophages since there is evidence that FABP4 and PPARG are in a positive feedback loop both in macrophages [18] and in monocytes, which differentiate into macrophages [45]. Thus, our study suggests that FABP4 has at least 2 roles in adipocytes: to transport and buffer fatty acids in mature cells during lipolysis, and at very low levels, to act in adipocyte precursor cells as a signaling mediator that transfers fatty acids to PPARG and increase its activity to promote differentiation. Interestingly, in the ASE neurons, which are important for chemotaxis in *Caenorhabditis elegans*, a critical factor for maintaining the differentiated state was found to also initiate the differentiation program, supporting that the mechanism we identified could be more universal [46].

**Fig 7:**
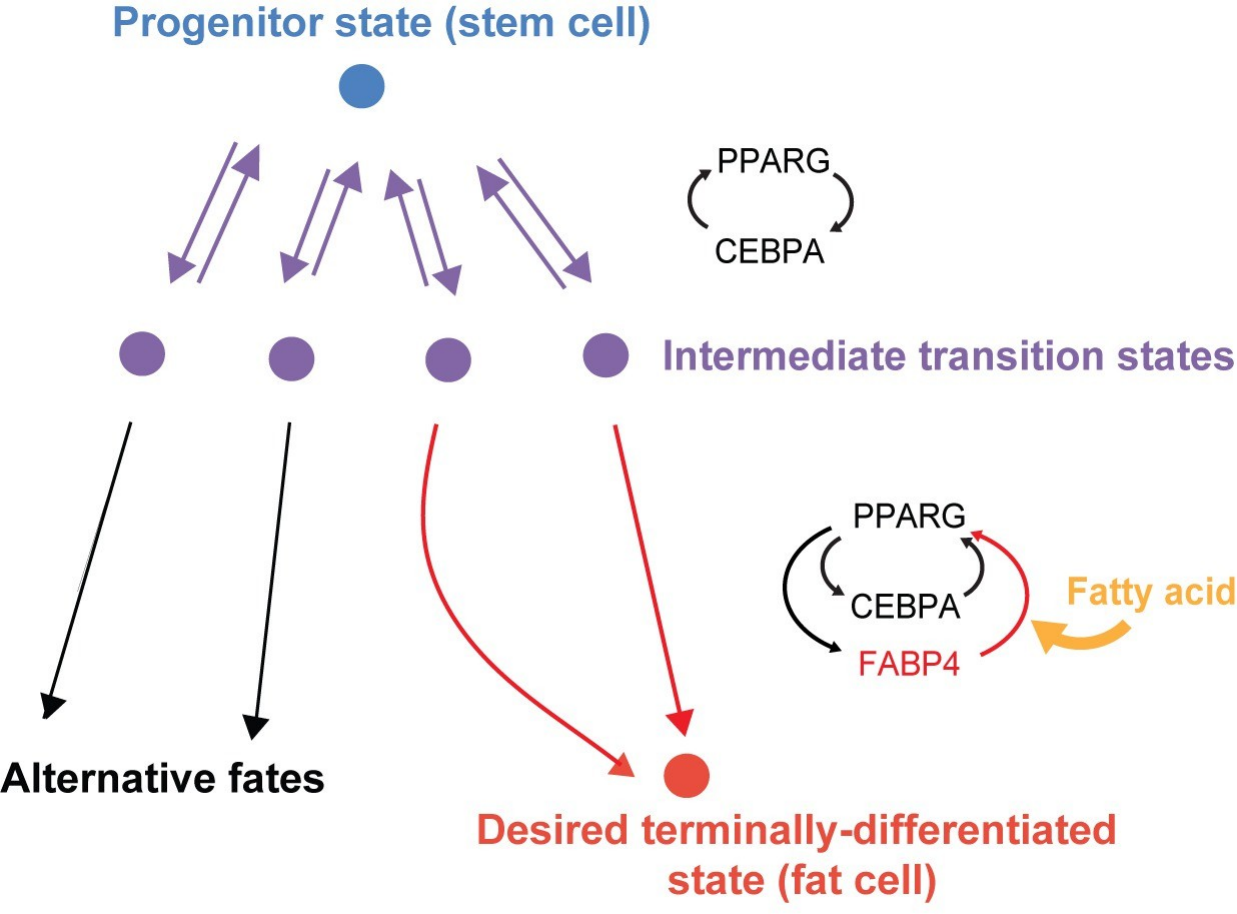
Model for how FABP4 functions as an attractor molecule for the terminally differentiated state. When preadipocytes are stimulated to differentiate, engagement of the CEBPA-PPARG feedback loop allows the cells to reach an intermediate transition state. The cells can only reach the terminally differentiated final adipocyte state if the FABP4-PPARG feedback also engages.

Interestingly, we found that the FABP4-PPARG feedback engages many hours after the CEBPA-PPARG positive feedback, offering a possible explanation of how to selectively achieve the mature adipocyte fate despite that PPAR and CCAAT/enhancer-binding protein transcription factors such as PPARG and CEBPA are common in many cell types [4,47–50]. It is possible that in the many cell types that express PPARG and CEBPA, PPARG levels can only reach an intermediate level without FABP4 engagement as we showed in Fig 3F and summarized in Fig 7. Only when the FABP4-PPARG positive feedback engages can PPARG and FABP4 levels to rise so strongly and rapidly that the differentiating cell can selectively reach the final adipocyte differentiated state.

Our findings may have implications for treating insulin resistance, diabetes, and other metabolic diseases for which defective adipogenesis and lack of functioning fat cells to properly store and release lipids is a hallmark. Drugs such as rosiglitazone that potently and directly activate PPARG are believed to treat insulin resistance in part by promoting adipogenesis and fat cell function, but they have side effects since PPARG is critical in other differentiation and regulatory processes in different tissues. Our finding that adipogenesis is selectively driven by a druggable and late-acting adipocyte specific factor FABP4 may ultimately end up being a useful selective strategy to therapeutically control adipogenesis.

Our study further addressed the question of how FABP4 can regulate PPARG since previous studies have yielded contradictory results. Some studies suggested that FABP4 up-regulates PPARG expression and activity in adipocytes [19,22,24], whereas others suggested that FABP4 suppresses PPARG expression and activity [20,51]. We showed that knocking down or knocking out both FABP4 and FABP5 in OP9 cells and 3T3-F442A results in a significant suppression of adipogenesis. This first appeared to contradict earlier results that showed that FABP4/FABP5 KO cells can still effectively differentiate when rosiglitazone was added along with the DMI [42]. Our study provides an explanation for this result, since we found that these DBKO cells fail to respond to DMI but differentiate in response to the PPARG activator rosiglitazone that was included in the previous experiments. Together, these results suggest that rosiglitazone can bind directly to the lipid-binding pocket of PPARG and may therefore bypass the need for FABP4 to bring fatty acids to PPARG and activate PPARG. Such a mechanism is plausible as fatty acids have low water solubility while rosiglitazone was developed as a partially water-soluble compound. Together with our knockout and rescue analysis, we also used live-cell imaging and demonstrated a rapid increase of endogenously tagged PPARG activity mediated by rosiglitazone. Thus, by mediating direct activation, rosiglitazone can bypass the need for FABP4. We therefore conclude that FABP4 has a dual role in adipocytes, as an essential lipid buffer and transporter that promotes lipid metabolism in the differentiated state, and also as an essential co-factor for terminal cell differentiation that drives precursor cells towards a specific commitment step and then helps maintain adipocyte cell identity.

### Experimental model details

OP9 mouse stromal cell line [52]

3T3-F442A mouse preadipocyte cell [53]

Immune-deficient mouse-J:NU (Jackson Labs, Cat #007850).

### Method details

#### Cell culture and differentiation

OP9 cells were cultured according to previously published protocols [17,22,27,52]. OP9 cells were cultured in 20% fetal bovine serum (FBS) in growth media consisting of MEM-α (Invitrogen, #12561) and 100 units/ml penicillin, 100 μg/ml streptomycin, and 292 μg/ml L-glutamate (Invitrogen, # 10378–016). To induce differentiation of OP9 cells using linoleic acid, confluent cells were treated with desired concentrations of bovine serum albumin adsorbed linoleic acids (Sigma Cat #L9530) plus 10% FBS. To induce differentiation of OP9 cells using the standard 96-hour DMI protocol, confluent cells were treated with a differentiation medium containing a commonly used DMI (dexamethasone/IBMX/insulin) stimulus to initiate adipogenesis. DMI consists of dexamethasone (dex), a synthetic glucocorticoid; 3-isobutyl-1-methylxanthine (IBMX), an inhibitor of phosphodiesterase that increases cAMP levels; and insulin. Applying the DMI stimulus consisted of replacing the media on the cells with growth media plus 10% FBS, 0.25 mM IBMX (Sigma Cat # 7018), 1 μm dexamethasone (Sigma Cat #D1756), and 1.75 nM insulin (Sigma Cat # I6634) (Stimulus I). Forty-eight hours after initiating differentiation, Stimulus I was removed from the cells and was replaced with Stimulus II consisting of growth media plus 10% FBS and 1.75 nM insulin for another 48 hours. As noted in some experiments, rosiglitazone (Cayman, United States of America) was added to the media to result in a final concentration of 1 μm.

3T3-F442A preadipocytes were grown and differentiated according to established protocols [33]. Briefly, 3T3-F442A cells were cultured in 10% bovine calf serum in growth media consisting of DMEM, 2 mM L-glutamine, 100 U/ml penicillin, and 100 U/ml streptomycin. Cells were passaged when pre-confluent. For differentiation, preadipocytes were grown to confluency on plates coated with collagen-1 (Advanced BioMatrix, 5005). When confluent, differentiation was induced by changing media to growth media + insulin (5 μg/ml) for 4 days. After day 4, the media was replaced with just growth media. This latter media was replaced every 2 days on the cells until the cells were fully differentiated, which occurred typically around day 10 after induction of differentiation.

### Generation of siRNA-resistant FABP4 overexpression construct

We obtained a commercially synthesized a FABP4 coding sequence (CDS) gene-block where all the siRNA-targeted sequences were changed to synonymous codons. This siRNA-resistant FABP4 CDS was then cloned into the pEGFP-C1 vector backbone using a standard Gibson assembly protocol between the restriction sites NheI and EcoRI (this removed EGFP coding sequence). As a control, we used an EYFP coding sequence cloned into the same vector backbone containing between the same restriction sites.

### siRNA and DNA transfection

#### (A) siRNA-mediated gene silencing

siRNA targeting FABP5, PPARG, CEBPB, CEBPA, FABP4, and the AllStars Negative Control siRNA were purchased from Qiagen or Dharmacon. Cells were transfected by reverse-transfection using Lipofectamine RNAiMax (Invitrogen). Briefly, our reverse-transfection protocol per well is as follows: mix 20 μl of Opti-MEM media (Thermo Fisher Scientific), 0.2 μl of a 10 μm siRNA stock solution, and 0.5 μl of RNAiMax. The solution was incubated at room temperature for 10 minutes, and then 80 μl of culture media containing the desired number of cells per well was added. Then, the entire (100 μl) volume was plated into 1 well of a Costar 96-well plate (#3904). For live-cell imaging experiments, reagents and cells were doubled due to the larger well volume of Ibidi μ-Plate (#89626). The siRNA/RNAiMax mixture was left on the cells for 24 hours before being aspirated away and replaced with fresh media for further tests.

#### (B) DNA transfection and DNA/siRNA co-transfection

Cells were plated in 96-well plate about 24 hours before transfection. A total of 100 ng of midi-prepped DNA (w/ or w/o 2 pmol siRNA) was transfected for each well using Lipofectamine 2000 (Invitrogen) in Opti-MEM media according to the manufacturer’s protocol. The transfection mixture was left on the cells for 24 hours before being aspirated away and replaced with fresh media for further tests.

### Oil Red O staining

To determine lipid accumulation, 3T3-F442A preadipocytes were differentiated for 10 days and stained with Oil Red O. The cells were washed with phosphate-buffered saline (PBS) and fixed using 4% paraformaldehyde (PFA) for 1 hour. Cells were washed using double-distilled H_2_O and incubated with the filtered Oil Red O solution (Cat #O0625, Sigma) for 1 hour. Cells were washed twice with ddH_2_O and analyzed by bright-field microscopy.

### De novo adipogenesis in vivo

To induce de novo fat pad formation, preadipocytes were grown to near confluency and resuspended in PBS; 3 × 10^7^ preadipocytes were injected subcutaneously at the sternum of 8-week-old male athymic mice (Cat. #007850, Jackson Laboratory) as described previously [34]. After 4 weeks, mice were anesthetized with isoflurane and sacrificed by cervical dislocation. Fat pads derived from the implanted cells were excised and weighed.

### Generation of FABP4-KO OP9 cells and FABP4-KO, FABP5-KO, and DBKO 3T3-422A cells

The CRISPR-Cas9 constructs targeting FABP4 and FABP5 were generated based on a previously described protocol [54]. Briefly, different guide RNAs targeting FABP4 N-terminus or FABP5 N-terminus were designed (crispr.mit.edu). Oligos including the sequences for targeting FABP4 were annealed and cloned into pSpCas9n (BB)-2A-GFP (PX461; Addgene #48140). Oligos including the sequences for targeting FABP5 were annealed and cloned into pSpCas9(BB)-2A-miRFP670 (Addgene #91854). Constructs were transfected into 3T3-F442A preadipocytes or OP9 preadipocytes via electroporation using the Amaxa Cell Line Nucleofector Kit V (Lonza, Cologne, Germany). Afterwards, cells were grown for 2 days in DMEM, 10% BS, and 100 U/ml penicillin/streptomycin for 3T3-F442A, or in MEM-alpha with L-glutamine, 20% FBS, and 100 U/ml penicillin/streptomycin for OP9 cells. FACS-sorting was performed to select for construct marker (GFP and/or iRFP expression), and cells were seeded in 96-well plates (1 cell/well) to analyze them for FABP4 and/or FABP5 expression. Single cells that did not harbor any FABP4-KO were expanded to obtain Control-KO clones. Successfully transfected cells were sorted for positive cells. The loss of FABP4, FABP5, or both proteins were confirmed by western blot analysis (Fig 1). To characterize the precise alterations caused by CRISPR at the genomic level, Sanger sequencing using TA cloning [55] was performed for each cell clone. Specifically, genomic DNA was first extracted using DNeasy Blood & Tissue Kits (#69504, Qiagen) by following the protocol from the manufacture. Cut-site analysis was performed by PCR-amplifying the region targeted by the guide RNA (primers listed in S5 Table), and then TOPO cloning (#K450002, Invitrogen) was applied to sequence individual alleles found within the clone. The analysis of sequencing results was included in S1 and S2 Figs.

### Workflow used to generate mouse OP9 cells with endogenously tagged PPARG and FABP4 (S4 Fig)

Previously, CRISPR-mediated genome editing had been used to tag the N-terminus of endogenous PPARG in OP9 cells [17]. These cells had also been stably transfected with a H2B-mTurquoise (CFP) as a nuclear marker to facilitate cell tracking. We now used these cells and tagged the C-terminus of FABP4 with mKate2, a bright red fluorescent protein [56].

#### (A) Construction of DNA plasmids

To carry out CRISPR genome editing to generate FABP4-mkate2, we used the “double nickase” system that uses 2 different guide RNAs that create adjacent and opposing nicks in the DNA at the site of insertion [17,57]. To carry out double-nickase genome editing, 2 different targeting sequences (sgRNA) directed to the FABP4 locus were designed as described above and inserted into the guide RNA site of 2 plasmids, pX335-U6-Chimeric_BBCBh-hSpCas9n (pX335) (Addgene plasmid #42335) encoding the SpCas9 D10A nickase. Oligonucleotide duplexes encoding each desired targeting sequence were ligated into the BbsΙ cut sites of px335. Construction and design of the donor template were carried out based on our previously established protocol [17]. The DNA repair template to promote homology directed repair (HDR)-mediated insertion of the fluorescent protein (mKate2) was constructed by inserting the cDNA of mKate2-3xGly flanked by two 800 bp homology arms into the entry vector backbone pENTR1a (Addgene Plasmid #17398). The pENTR1a backbone vector was digested with EcoRΙ-HF and BamHΙ-HF (NEB) and assembled together with 3 DNA fragments by using Gibson assembly. All constructs were validated by sequencing.

#### (B) Transfection

Approximately 1 mg of each of the 2 pX335 guide RNA/SpCas9n constructs and 5 mg of the mKate2 donor template was transfected into 1 million OP9 cells using Lipofectamine 2000 (Invitrogen) following the manufacturer’s protocol.

#### (C) Clone selection by single-cell FACS

Clone selection was carried out based on our previous protocol [17]. In briefly, 7 days post transfection, single cells expressing mKate2 were sorted into separate wells of 96-well culture plates and allowed to grow. We chose to wait 7 days post transfection to avoid false-positive fluorescent signal originating from the un-integrated donor DNA plasmid.

#### (D) Stimulus response test

Once the single-cell colonies grew to 50% confluency, each colony was passaged into wells on 2 different 96-well plates. One plate was used to expand the colonies, and the other half was imaged using a Molecular Devices MicroXL fluorescence imaging system to select for clones with correct localization of the citrine signal and the appropriate response to stimuli. Before imaging, PPARG/FABP4 clones were stimulated for 24 hours with DMI to induce expression of citrine-PPARG/FABP4-mKate2.

#### (E) Differentiation capacity test

Clones that expressed citrine were further characterized for their differentiation capacity using the standard 4-day adipocyte differentiation protocol detailed under “Cell Culture and Differentiation.” Clones that acquired mature adipocyte morphology and accumulated lipid droplets in response to DMI treatment were expanded and subjected to further validation steps.

#### (F) Validation of citrine-PPARG/FABP4-mKate2 clones

We first performed genomic PCR with different primer sets to look at the genotype and verify correct insertion of mKate2 into the FABP4 locus. The first PCR was performed using primers annealing to regions flanking the site where mKate2 is inserted (S5 Table and S6A Fig). Using this set of primers, FABP4 tagged clone2 or 4 were shown to be heterozygous, and the PCR products were subjected to sequencing. The second set of PCRs was performed using primers annealing end of the inserted mKate2 (S6 Table and S6B Fig). Both reactions resulted in a single band, indicating that mKate2 was correctly inserted into the FABP4 locus in clone2 or 4. Next, western blot analysis of FABP4-mKate2 clone2 was performed using anti-RFP antibody and anti-FABP4 to verify protein expression and to check for the correct molecular weight of the tagged protein (S6C and S6D Fig). The size compatible with the correct predicted molecular weight of mKate2-protein fusion were shown for each clone. The anti RFP blot shows the expression of tagged-FABP4 and did not detect any free RFP. Next, Southern blot analysis was performed to confirm locus-specific knock-in using a probe directed toward mKate2. All examined clones showed the presence of a specific copy of mKate2 within the genome, as evidenced by the detection of a single band of expected size (approximately 2 kb) in the Southern blot (S6E Fig). Finally, immunohistochemistry analysis was used to validate co-localization of the mKate2 fluorescence signal with the immunohistochemistry signal of the FABP4 proteins throughout 4 days of differentiation. Since it passed all the validation criteria described above, the FABP4-2 clone was used for all the time course measurements in the current manuscript.

### Generation of OP9 citrine-CEBPA and OP9 FABP4-citrine cell lines

To generate OP9 cells in which endogenous CEBPA is tagged at the N-terminus with citrine and OP9 cells in which endogenous FABP4 is tagged at the C-terminus with citrine, we followed the same protocol used to tag the N-terminus of endogenous PPARG in OP9 cells with citrine [17]. Single clones were isolated and tested in the same manner as described above.

### Measuring protein decay rates using cyclohexamide

To obtain protein decay rates, 10,000 OP9 cells were seeded in 96-well plates (1 plate for each time point). Cells were induced to differentiate with linoleic acid for 48 hours. Then, cyclohexamide was added at a final concentration of 30 μm. Cells were fixed and stained at different times after addition of cyclohexamide, and immunofluorescence was used to quantify protein concentration. Half-lives were obtained by fitting first order exponential decay curves to the data.

### Immunoblotting

For SDS-PAGE, 20 ug protein per lane was used. Proteins were detected with anti-PPARG (1:1,000, Santa Cruz Biotechnology, sc-7273), anti-FABP4 (1:1,000 R&D Systems, AF1443), anti-FABP5 (1:1,000 Cell Signaling, 5174), anti-GFP (1:1,000 Abcam, ab290), and anti-tRFP (1:1,000 Evrogen EVN-AB233-C100).

### FABP4 gain-of-function in pre-adipocytes via CRISPRa SAM

Murine mesenchymal C3H10T1/2 cells stably expressing the CRISPRa-SAM complex were used for gain of function experiments following a previously established protocol [36]. Briefly, to make the sable cell line, the CRISPRa-SAM components (dCas9-VP64 and MS2-P65-HSF1) were delivered in lentiviruses by plasmid co-transfection of C3H10T1/2 cells. The resulting cell line (C3H10T1/2-CRISPRa-SAM) can be used for activation of endogenous genes via chemical transfection with a sgRNA-containing plasmid. C3H10T1/2-CRISPRa-SAM cells were maintained in high glucose-DMEM media containing FBS (10%), penicillin-streptomycin (1%), and blasticidin (2.5 ug/ml) and hygromycin (200 ug/ml) to ensure a retained dCas9-SaM expression. sgRNA sequences targeting FABP4 promoter regions were designed using the “SAM genome engineering online tool” (http://sam.genomeengineering.org/database/), and annealed and ligated into the backbone vector following the original protocol [58]. Correct insertion was verified by sequencing of plasmids. The sequence used was FABP4 (NM_024406.3) CATACAGGGTCTGGTCATGA. Cell transfections were performed as previously described [36].

For gene expression analysis (RT-qPCR), 300,000 cells were seeded per well into a 12-well plate (day -3), transfected at confluence (day -2) using Mirus TransIT-X2 transfection reagent (3 ul per well; Mirus Bio LLC, USA) and 250 ng plasmid DNA (either empty vector or FABP4 sgRNA). For staining experiments, 2,000 cells were seeded per well into a 96-well Costar Plastic plate. The day after, the cells were transfected with a total of 50 ng sgRNA per well using 0.25 μl TransIT-X2 reagent per well. Two days following transfection at day 0, all cells received either only DMI or DMI and rosiglitazone (1 μm) to induce adipogenesis. The non-stimulated cells were used as a control. At day 2, the media was replaced with media containing only insulin. At day 4, the cells were either fixed for protein staining or lysed for mRNA analysis.

### RNA isolation and quantitative polymerase chain reaction (qPCR)

Cells were lysed in RLT buffer (β-Me, 1:100) on ice; RNAs were isolated using RNeasy spin columns (Qiagen, CAT. 74106) and cDNA synthesized using the qScript kit (Quantabio, Cat. 101414–098) according to the manufacturer’s instructions. Real-time PCR was performed using the GoTaq qPCR Master Mix (Promega, Cat. M3001) in LightCycler 480-Roche System according to the supplier’s manual. Raw CT data were normalized to 18S reference gene following the ΔΔ-CT calculation. PCR primer sequences were synthesized by Elim Biopharmaceuticals (California, USA) and are listed in S8 Table.

### Fluorescent imaging

Imaging was conducted using an ImageXpress MicroXL (Molecular Devices, USA) with a 10× Plan Apo 0.45 NA objective. Live fluorescent imaging was conducted at 37°C with 5% CO_2_. A camera bin of 2 × 2 was used for all imaging condition. Cells were plated in optically clear 96-well plates: plastic-bottom Costar plates (#3904) for fixed imaging or Ibidi μ-Plate (#89626) for live imaging. Living cells were imaged in FluoroBrite DMEM media (Invitrogen) with 10% FBS, 1% penicillin/streptomycin, and insulin to reduce background fluorescence. Images were taken every 12 minutes in different fluorescent channels: CFP, YFP, and/or RFP. Total light exposure time was kept less than 700 ms for each time point. Four, non-overlapping sites in each well were imaged. Cell culture media were changed at least every 48 hours.

### Determining the percent of differentiated cells in fixed-cell experiments

The percent of differentiated cells for both OP9 and 3T3-F442A cells in fixed-cell experiments is calculated as described in [17,27]. Briefly, immunocytochemistry was carried out to measure single-cell abundance of PPARG. PPARG levels at the end of a differentiation experiment typically exhibit a bimodal distribution. PPARG threshold is calculated as the center between the 2 peaks in the PPARG histogram. The percent of differentiated cells was assessed by counting cells that above the PPARG threshold and dividing by the total number of cells (Fig 1D).

### Imaging data processing for live-cell time courses

Data processing of fluorescent images was conducted in MATLAB (MathWorks). Unless stated otherwise, fluorescent imaging data were obtained by automated image segmentation, tracking, and measurement using the MACKtrack package for MATLAB. Quantification of PPARG- and FABP4-positive cells in fixed samples was based on quantification of mean fluorescence signal over nuclei. Cells were scored as PPARG- and FABP4-positive if the marker expression level was above a preset cutoff determined by the bimodal expression at the end of the experiment.

For live-imaging data of OP9 cells, the CFP channel capturing H2B-mTurquoise fluorescence was used for nuclear segmentation and cell tracking. Obtained single-cell traces were filtered to removed incomplete or mistracked traces according to the following criteria: cells absent within 6 hours of the endpoint, cell traces that started more than 4 hours after the first time point, and cells that had large increase or decrease in PPARG intensity normalized to the previous time point. If cells were binned according to their PPARG expression, cells were binned based on their mean nuclear PPARG expression at the described time points. The percent of PPARG high cells was assessed by counting cells that above the PPARG threshold at that time point and dividing by the total number of cells at that time point.

### Estimating the value of PPARG threshold for live-cell experiments

The value of PPARG threshold is calculated for live-cell experiments as described in [17,27]. Briefly, PPARG values at the end of a differentiation experiment typically exhibit a bimodal distribution. PPARG values at the last frame of the experiment were fit to a 2 component Gaussian mixture model. Cells were then classified as either differentiated or undifferentiated based on whether they more closely associated with the high or low component of the mixture model, respectively. The value of PPARG threshold was assessed as the value of PPARG at the 48-hour time point, before the stimuli was removed, that predicted the final differentiation classification with a false-positive rate of less than 5%. If there are multiple conditions are present in the same experiment, the Gaussian mixture model was fitted to the non-stimulated control, and the threshold value was selected based on the non-stimulated control model and applied to all other conditions.

### Statistics

#### All statistical parameters are reported in the figures and figure legends. Statistical analysis was performed in MATLAB R2022a (MathWorks) or R

In Fig 4, to determine the time point at which the PPARG dynamics in siRNA knockdown versus control cells start to become distinct from each other, we calculated the *p*-value by Kruskal–Wallis test at each time point. By plotting time versus log(*p*-value), we found −20 could be a suitable threshold for the *p*-value. Then, we searched for the first time point at which log(*p*-value) became less than −20 and used this time point as the time at which the PPARG between these 2 populations started to become significantly distinct from each other. The same scrambled control time courses were used to compare against the 3 knockdown time courses (siCEBPA, siCEBPA, and siFABP4) in Fig 4A.

### Ethics statement

All animal work was reviewed and approved by the Stanford University Institutional Animal Care and Use Committee (IACUC) under Assurance Number A3213091.

## Supporting information

S1 Fig Additional experiments supporting that FABP4 regulates PPARG expression and adipogenesis in OP9 cells. (A) The analysis of sequencing results for the OP9 FABP4-KO clone used in Fig 1. (B) Measurements of FABP5 levels by immunocytochemistry show that FABP5 is up-regulated in FABP4 knockout cells. Bar plots are mean +/–SEM from 3 technical replicates with approximately 2,000 cells per replicate. (C) Validation of the efficiency of FABP5 siRNA in OP9 preadipocytes assessed by carrying out immunocytochemistry. Bar plots show mean +/–SEM from 3 technical replicates with approximately 5,000 cells per replicate. (B, C) Unpaired *t* test, **, *p* < 0.01; ***, *p* < 0.001. (D, E) Knockout of FABP4 and FABP5 impairs adipogenesis in OP9 preadipocyte cells induced to differentiate by the standard DMI protocol. The addition of 1 μm rosiglitazone rescues the loss of adipogenesis in FABP4-KO OP9 cells and in FABP4-KO OP9 cells transfected with FABP5 siRNA. Scale bar is 90 μm. The data underlying the graphs in the figure can be found in https://zenodo.org/record/7012787#.Y2I5I0zP3b0.
(PDF)

S2 Fig Additional experiments supporting that FABP4 regulates PPARG expression and adipogenesis in 3T3-F442A cells. (A, B) The analysis of sequencing results for the 3T3-F442A FABP4-KO (A) and FABP4/FABP5 DBKO (B) clones used in Fig 1. (C, D) Knockout of FABP4 and FABP5 impairs adipogenesis in 3T3-FF42A preadipocyte cells induced to differentiate by the standard protocol of adding insulin. The addition of 1 μm rosiglitazone rescues the loss of adipogenesis in FABP4-KO and FABP4/FABP5 DBKO 3T3-F442A cells. Scale bar is 30 μm.
(PDF)

S3 Fig Data for the second clones of 3T3-F442A Control-KO, FABP4-KO, and FABP4/FABP5 DBKO cells. In addition to the clones for the 3T3-F442A Control-KO, FABP4-KO, and FABP4/FABP5 DBKO cells as shown in Figs 1 and 6 and S2, a second clone was validated and tested for each cell lines in the same manner. Cells were grown to 2 days post-confluence and were induced to differentiate by addition of insulin (A–C) or insulin + 1 μm rosiglitazone (D). Cells were assayed at day 6 post-induction. (A) The loss of FABP4 and FABP5 proteins was validated by western blot analysis. β-actin was used as a loading control. (B) The extent of adipogenesis was assessed by Oil Red O staining for intracellular lipid accumulation. Scale bar, 100 μm. (C, D) The percent of differentiated cells were assessed by counting cells that above the PPARG threshold and dividing by the total number of cells (see Methods). Bar plots show mean +/–SEM from 3 technical replicates with approximately 1,000 cells per replicate. Data is representative of 3 independent experiments. The data underlying the graphs in the figure can be found in https://zenodo.org/record/7012787#.Y2I5I0zP3b0.
(PDF)

S4 Fig Increasing FABP4 expression by CRISPRa increases PPARG expression. C3H10T1/2-CRISPRa-SAM cells were transfected with either empty vector (Control) or guide RNA targeting the FABP4 promoter region (FABP4-OE); 48 hours later, the cells were induced to differentiate using 96-hour DMI protocol as in Fig 1B. (A) qRT-PCR to measure PPARG and Adiponectin (AdipoQ) expression. Data are normalized to 18s expression. Three biological replicates were used. A Student *t* test, 2 tail, type 2 was applied for statistical analysis. Values represent means ± SEM. ns, *p* > 0.05, ****p < 0.0001. (B) Immunocytochemistry to assess PPARG expression (red), lipid accumulation using Bodipy (green), and Hoechst to mark the nuclei (blue). Scale bar, 50 μm. The data underlying the graphs in the figure can be found in https://zenodo.org/record/7012787#.Y2I5I0zP3b0.
(PDF)

S5 Fig Workflow for using CRISPR-mediated genome editing to generate and validate single clones with endogenous PPARG tagged with citrine(YFP) (already existing cells from Bahrami-Nejad and colleagues, 2018) and endogenous FABP4 tagged with mKate2(RFP). The different steps in the workflow are described in detail in the Methods section.
(TIF)

S6 Fig Validation of FABP4-mKate2(RFP) OP9 cell clones. The different steps of the validation procedure are detailed in the Methods section. The data underlying the graphs in the figure can be found in https://zenodo.org/record/7012787#.Y2I5I0zP3b0.
(PDF)

S7 Fig Probability analysis to determine the timing of feedback engagement. To determine the timing at which the PPARG dynamics in siRNA knockdown cells start to become diverged from that in negative control cells, we calculated the *p*-value by Kruskal–Wallis test at each time point. By plotting logarithm of *p*-value versus time, we found −20 could be a suitable value as the threshold. Then, we search for the time point at which the logarithm of *p*-value become, at the first time, less than −20, and use this very time point (red dashed line, indicated as separated time) as the timing from which the PPARG dynamics between knockdown cells and negative control cells start to get remarkably diverged. The data underlying the graphs in the figure can be found in https://zenodo.org/record/7012787#.Y2I5I0zP3b0.
(PDF)

S8 Fig Validation of FABP4 knockdown in OP9 cells. Citrine-PPARG and FABP4-mKate2 cells were transfected with FABP4-targeted or control siRNA and were imaged while being induced to differentiate by the standard DMI protocol. Plots are the median of FABP4 abundance from about 8,000 cells. The data underlying the graphs in the figure can be found in https://zenodo.org/record/7012787#.Y2I5I0zP3b0.
(PDF)

S1 Table Plasmids. (PDF)

S2 Table Oligonucleotide sequences used to insert sgRNA sequences into the px335. Guide sequences are targeted to the FABP4 C-terminal. The underlined and italicized nucleotides denote the overhang for ligation of the oligonucleotide duplex into the px335 guide sequence insertion site.
(PDF)

S3 Table Oligonucleotide sequences used for CRISPR KO of FABP4 or FABP5. (PDF)

S4 Table Primers used for PCR amplification of fragments that were joined by Gibson assembly to create donor vectors to insert citrine at the C-terminal of FABP4 via homologous recombination. (PDF)

S5 Table Primers used for genomic PCR analysis of the FABP4 CRISPR clones. (PDF)

S6 Table Primers used for genomic PCR analysis to verify the fluorophore integration sites of the FABP4 tagged clones. (PDF)

S7 Table Primers used for the PCR amplification of a 504 bp probe directed towards citrine and 505 bp probe directed towards mKate2. (PDF)

S8 Table List of primer sequences used for quantitative PCR-based gene expression analysis. (PDF)

S9 Table Cell lines. (PDF)

S10 Table Key resources table. (PDF)

S1 Raw images Raw images of western blots. (PDF)

## Abbreviations

DBKO: double knockout
FBS: fetal bovine serum
HDR: homology directed repair
HSL: hormone sensitive lipase
MEF: mouse embryonic fibroblast
MSC: mesenchymal stem cell
PBS: phosphate-buffered saline
PFA: paraformaldehyde
qPCR: quantitative polymerase chain reaction
ROC: receiver operating characteristic
sgRNA: single guide RNA
siRNA: small interfering RNA

## References

[1] Buttitta LA, Edgar BA. Mechanisms controlling cell cycle exit upon terminal differentiation. Curr Opin Cell Biol. 2007;19:697–704. doi: 10.1016/j.ceb.2007.10.004 18035529 PMC2700000

[2] Hu M, Krause D, Greaves M, Sharkis S, Dexter M, Heyworth C, et al. Multilineage gene expression precedes commitment in the hemopoietic system. Genes Dev. 1997;11:774–785. doi: 10.1101/gad.11.6.774 9087431

[3] Komori T. Runx2, A multifunctional transcription factor in skeletal development. J Cell Biochem. 2002;87:1–8. doi: 10.1002/jcb.10276 12210716

[4] Poulsen Lla C, Siersbæk M, Mandrup S. PPARs: Fatty acid sensors controlling metabolism. Semin Cell Dev Biol. 2012;23:631–639. doi: 10.1016/j.semcdb.2012.01.003 22273692

[5] Sagner A, Briscoe J. Establishing neuronal diversity in the spinal cord: a time and a place. Development. 2019;146:dev182154. doi: 10.1242/dev.182154 31767567

[6] Brackston RD, Lakatos E, Stumpf MPH. Transition state characteristics during cell differentiation. Maini PK, editor. PLoS Comput Biol. 2018;14:e1006405. doi: 10.1371/journal.pcbi.1006405 30235202 PMC6168170

[7] MacArthur BD, Ma’ayan A, Lemischka IR. Systems biology of stem cell fate and cellular reprogramming. Nat Rev Mol Cell Biol. 2009;10:672–681. doi: 10.1038/nrm2766 19738627 PMC2928569

[8] Mojtahedi M, Skupin A, Zhou J, Castaño IG, Leong-Quong RYY, Chang H, et al. Cell Fate Decision as High-Dimensional Critical State Transition. PLoS Biol. 2016;14:e2000640. doi: 10.1371/journal.pbio.2000640 28027308 PMC5189937

[9] Moris N, Pina C, Arias AM. Transition states and cell fate decisions in epigenetic landscapes. Nat Rev Genet. 2016;17:693–703. doi: 10.1038/nrg.2016.98 27616569

[10] Kutejova E, Sasai N, Shah A, Gouti M, Briscoe J. Neural Progenitors Adopt Specific Identities by Directly Repressing All Alternative Progenitor Transcriptional Programs. Dev Cell. 2016;36:639–653. doi: 10.1016/j.devcel.2016.02.013 26972603 PMC4819439

[11] Aydin B, Mazzoni EO. Cell Reprogramming: The Many Roads to Success. Annu Rev Cell Dev Biol. 2019;35:433–452. doi: 10.1146/annurev-cellbio-100818-125127 31340126

[12] Briggs JA, Li VC, Lee S, Woolf CJ, Klein A, Kirschner MW. Mouse embryonic stem cells can differentiate via multiple paths to the same state. eLife. 2017;6:e26945. doi: 10.7554/eLife.26945 28990928 PMC5648529

[13] Rosen ED, Spiegelman BM. What We Talk About When We Talk About Fat. Cell. 2014;156:20–44. doi: 10.1016/j.cell.2013.12.012 24439368 PMC3934003

[14] Coe NR, Bernlohr DA. Physiological properties and functions of intracellular fatty acid-binding proteins. Biochim Biophys Acta. 1998;1391:287–306. doi: 10.1016/s0005-2760(97)00205-1 9555061

[15] Hotamisligil GS, Bernlohr DA. Metabolic functions of FABPs—mechanisms and therapeutic implications. Nat Rev Endocrinol. 2015;11:592–605. doi: 10.1038/nrendo.2015.122 26260145 PMC4578711

[16] Uhlen M, Fagerberg L, Hallström BM, Lindskog C, Oksvold P, Mardinoglu A, et al. Tissue-based map of the human proteome. Science. 2015;347:1260419. doi: 10.1126/science.1260419 25613900

[17] Bahrami-Nejad Z, Zhao ML, Tholen S, Hunerdosse D, Tkach KE, van Schie S, et al. A Transcriptional Circuit Filters Oscillating Circadian Hormonal Inputs to Regulate Fat Cell Differentiation. Cell Metabolism. 2018;27:854–868.e8. doi: 10.1016/j.cmet.2018.03.012 29617644 PMC5889123

[18] Boss M, Kemmerer M, Brüne B, Namgaladze D. FABP4 inhibition suppresses PPARγ activity and VLDL-induced foam cell formation in IL-4-polarized human macrophages. Atherosclerosis. 2015;240:424–430. doi: 10.1016/j.atherosclerosis.2015.03.042 25897794

[19] Tan N-S, Shaw NS, Vinckenbosch N, Liu P, Yasmin R, Desvergne B, et al. Selective Cooperation between Fatty Acid Binding Proteins and Peroxisome Proliferator-Activated Receptors in Regulating Transcription. Mol Cell Biol. 2002;22:15. doi: 10.1128/MCB.22.14.5114-5127.2002 12077340 PMC139777

[20] Garin-Shkolnik T, Rudich A, Hotamisligil GS, Rubinstein M. FABP4 attenuates PPARγ and adipogenesis and is inversely correlated with PPARγ in adipose tissues. Diabetes. 2014;63:900–911. doi: 10.2337/db13-0436 24319114

[21] Tontonoz P, Hu E, Spiegelman BM. Stimulation of adipogenesis in fibroblasts by PPAR gamma 2, a lipid-activated transcription factor. Cell. 1994;79:1147–1156. doi: 10.1016/0092-8674(94)90006-x 8001151

[22] Ahrends R, Ota A, Kovary KM, Kudo T, Park BO, Teruel MN. Controlling low rates of cell differentiation through noise and ultrahigh feedback. Science (New York, NY). 2014;344:1384–1389. doi: 10.1126/science.1252079 24948735 PMC4733388

[23] Hotamisligil GS, Johnson RS, Distel RJ, Ellis R, Papaioannou VE, Spiegelman BM. Uncoupling of Obesity from Insulin Resistance Through a Targeted Mutation in *aP2*, the Adipocyte Fatty Acid Binding Protein. Science. 1996;274:1377–1379. doi: 10.1126/science.274.5291.1377 8910278

[24] Ayers SD, Nedrow KL, Gillilan RE, Noy N. Continuous Nucleocytoplasmic Shuttling Underlies Transcriptional Activation of PPARγ by FABP4. Biochemistry. 2007;46:6744–6752. doi: 10.1021/bi700047a 17516629

[25] Park BO, Ahrends R, Teruel MN. Consecutive Positive Feedback Loops Create a Bistable Switch that Controls Preadipocyte-to-Adipocyte Conversion. Cell Rep. 2012;2:976–990. doi: 10.1016/j.celrep.2012.08.038 23063366 PMC4959269

[26] Zhang Z-B, Sinha J, Bahrami-Nejad Z, Teruel MN. The circadian clock mediates daily bursts of cell differentiation by periodically restricting cell differentiation commitment. Proc Natl Acad Sci U S A. 2022;119:e2204470119. doi: 10.1073/pnas.2204470119 35939672 PMC9388110

[27] Zhao ML, Rabiee A, Kovary KM, Bahrami-Nejad Z, Taylor B, Teruel MN. Molecular Competition in G1 Controls When Cells Simultaneously Commit to Terminally Differentiate and Exit the Cell Cycle. Cell Rep. 2020;31:107769. doi: 10.1016/j.celrep.2020.107769 32553172 PMC8198760

[28] Furuhashi M, Saitoh S, Shimamoto K, Miura T. Fatty Acid-Binding Protein 4 (FABP4): Pathophysiological Insights and Potent Clinical Biomarker of Metabolic and Cardiovascular Diseases. Clin Med Insights Cardiol. 2014;8:23–33. doi: 10.4137/CMC.S17067 25674026 PMC4315049

[29] Coe NR, Simpson MA, Bernlohr DA. Targeted disruption of the adipocyte lipid-binding protein (aP2 protein) gene impairs fat cell lipolysis and increases cellular fatty acid levels. J Lipid Res. 1999;40:967–972. doi: 10.1016/S0022-2275(20)32133-7 10224167

[30] Shaughnessy S, Smith ER, Kodukula S, Storch J, Fried SK. Adipocyte Metabolism in Adipocyte Fatty Acid Binding Protein Knockout (aP2–/–) Mice After Short-Term High-Fat Feeding. Diabetes. 2000;49:8. 10866041 10.2337/diabetes.49.6.904

[31] Matsusue K, Peters JM, Gonzalez FJ. PPARβ/δ potentiates PPARγ-stimulated adipocyte differentiation. FASEB J. 2004;18:1477–1479. doi: 10.1096/fj.04-1944fje 15247146

[32] Tan N, Shaw NS, Vinckenbosch N, Liu P, Yasmin R, Desvergne B, et al. Selective Cooperation between Fatty Acid Binding Proteins and Peroxisome Proliferator-Activated Receptors in Regulating Transcription. Mol Cell Biol. 2002;22:5114–5127. doi: 10.1128/MCB.22.14.5114-5127.2002 12077340 PMC139777

[33] Djian P, Phillips M, Green H. The activation of specific gene transcription in the adipose conversion of 3T3 cells. J Cell Physiol. 1985;124:554–556. doi: 10.1002/jcp.1041240327 4044664

[34] Mandrup S, Loftus TM, MacDougald OA, Kuhajda FP, Lane MD. Obese gene expression at *in vivo* levels by fat pads derived from s.c. implanted 3T3-F442A preadipocytes. Proc Natl Acad Sci U S A. 1997;94:4300–4305. doi: 10.1073/pnas.94.9.4300 9113984 PMC20717

[35] Reznikoff CA, Brankow DW, Heidelberger C. Establishment and Characterization of a Cloned Line of C3H Mouse Embryo Cells Sensitive to Postconfluence Inhibition of Division. Cancer Res. 1973;33:3231–3238. 4357355

[36] Lundh M, Pluciñska K, Isidor MS, Petersen PSS, Emanuelli B. Bidirectional manipulation of gene expression in adipocytes using CRISPRa and siRNA. Mol Metab. 2017;6:1313–1320. doi: 10.1016/j.molmet.2017.07.001 29031730 PMC5641601

[37] Christy RJ, Yang VW, Ntambi JM, Geiman DE, Landschulz WH, Friedman AD, et al. Differentiation-induced gene expression in 3T3-L1 preadipocytes: CCAAT/enhancer binding protein interacts with and activates the promoters of two adipocyte-specific genes. Genes Dev. 1989;3:1323–1335. doi: 10.1101/gad.3.9.1323 2606350

[38] Rosen ED, Hsu C-H, Wang X, Sakai S, Freeman MW, Gonzalez FJ, et al. C/EBPalpha induces adipogenesis through PPARgamma: a unified pathway. Genes Dev. 2002;16:22–26. doi: 10.1101/gad.948702 11782441 PMC155311

[39] Farmer SR. Transcriptional control of adipocyte formation. Cell Metab. 2006;4:263–273. doi: 10.1016/j.cmet.2006.07.001 17011499 PMC1958996

[40] Schröter C, Rué P, Mackenzie JP, Arias AM. FGF/MAPK signaling sets the switching threshold of a bistable circuit controlling cell fate decisions in ES cells. Development. 2015:dev.127530. doi: 10.1242/dev.127530 26511924 PMC4689219

[41] Kliewer SA, Sundseth SS, Jones SA, Brown PJ, Wisely GB, Koble CS, et al. Fatty acids and eicosanoids regulate gene expression through direct interactions with peroxisome proliferator-activated receptors α and γ. Proc Natl Acad Sci U S A. 1997;94:4318–4323. doi: 10.1073/pnas.94.9.4318 9113987 PMC20720

[42] Furuhashi M, Tuncman G, Görgün CZ, Makowski L, Atsumi G, Vaillancourt E, et al. Treatment of diabetes and atherosclerosis by inhibiting fatty-acid-binding protein aP2. Nature. 2007;447:959–965. doi: 10.1038/nature05844 17554340 PMC4076119

[43] Kroker AJ, Bruning JB. Review of the structural and dynamic mechanisms of PPAR γ partial agonism. PPAR Res. 2015;2015. doi: 10.1155/2015/816856 26435709 PMC4578752

[44] Adida A, Spener F. Adipocyte-type fatty acid-binding protein as inter-compartmental shuttle for peroxisome proliferator activated receptor γ agonists in cultured cell. Biochim Biophys Acta. 2006;1761:172–181. doi: 10.1016/j.bbalip.2006.02.006 16574478

[45] Lamas Bervejillo M, Bonanata J, Franchini GR, Richeri A, Marqués JM, Freeman BA, et al. A FABP4-PPARγ signaling axis regulates human monocyte responses to electrophilic fatty acid nitroalkenes. Redox Biol. 2020;29:101376. doi: 10.1016/j.redox.2019.101376 31926616 PMC6926352

[46] Leyva-Díaz E, Hobert O. Transcription factor autoregulation is required for acquisition and maintenance of neuronal identity. Development. 2019;146:dev177378. doi: 10.1242/dev.177378 31227642 PMC6633604

[47] Argmann CA, Cock T-A, Auwerx J. Peroxisome proliferator-activated receptor gamma: the more the merrier? Eur J Clin Invest. 2005;35:82–92. doi: 10.1111/j.1365-2362.2005.01456.x 15667578

[48] Cirilli M, Bereshchenko O, Ermakova O, Nerlov C. Insights into specificity, redundancy and new cellular functions of C/EBPa and C/EBPb transcription factors through interactome network analysis. Biochim Biophys Acta. 2017;1861:467–476. doi: 10.1016/j.bbagen.2016.10.002 27746211

[49] Ramji DP, Foka P. CCAAT/enhancer-binding proteins: structure, function and regulation. Biochem J. 2002;365:561–575. doi: 10.1042/BJ20020508 12006103 PMC1222736

[50] Soccio RE, Chen ER, Lazar MA. Thiazolidinediones and the Promise of Insulin Sensitization in Type 2 Diabetes. Cell Metabolism. 2014;20:573–591. doi: 10.1016/j.cmet.2014.08.005 25242225 PMC4192012

[51] Kralisch S, Klöting N, Ebert T, Kern M, Hoffmann A, Krause K, et al. Circulating adipocyte fatty acid-binding protein induces insulin resistance in mice *in vivo*. Obesity. 2015;23:1007–1013. doi: 10.1002/oby.21057 25865078

[52] Wolins NE, Quaynor BK, Skinner JR, Tzekov A, Park C, Choi K, et al. OP9 mouse stromal cells rapidly differentiate into adipocytes: characterization of a useful new model of adipogenesis. J Lipid Res. 2006;47:450–460. doi: 10.1194/jlr.D500037-JLR200 16319419

[53] Green H, Kehinde O. An established preadipose cell line and its differentiation in culture II. Factors affecting the adipose conversion. Cell. 1975;5:19–27. doi: 10.1016/0092-8674(75)90087-2 165899

[54] Ran FA, Hsu PD, Wright J, Agarwala V, Scott D, Zhang F. Genome engineering using the CRISPR-Cas9 system. Nat Protoc. 2013;8:2281–2308. doi: 10.1038/nprot.2013.143 24157548 PMC3969860

[55] Giuliano CJ, Lin A, Girish V, Sheltzer JM. Generating Single Cell–Derived Knockout Clones in Mammalian Cells with CRISPR/Cas9. Curr Protoc Mol Biol. 2019;128:e100. doi: 10.1002/cpmb.100 31503414 PMC6741428

[56] Shcherbo D, Murphy CS, Ermakova GV, Solovieva EA, Chepurnykh TV, Shcheglov AS, et al. Far-red fluorescent tags for protein imaging in living tissues. Biochemical J. 2009;418:567–574. doi: 10.1042/BJ20081949 19143658 PMC2893397

[57] Ran FA, Hsu PD, Lin C-Y, Gootenberg JS, Konermann S, Trevino AE, et al. Double Nicking by RNA-Guided CRISPR Cas9 for Enhanced Genome Editing Specificity. Cell. 2013;154:1380–1389. doi: 10.1016/j.cell.2013.08.021 23992846 PMC3856256

[58] Konermann S, Brigham MD, Trevino AE, Hsu PD, Heidenreich M, Cong L, et al. Optical control of mammalian endogenous transcription and epigenetic states. Nature. 2013;500:472–476. doi: 10.1038/nature12466 23877069 PMC3856241

