# Supplementary material for: Early enforcement of cell identity by a functional component of the terminally differentiated state
Source: PLoS Biol. 2022 Dec 5;20(12):e3001900. doi: 10.1371/journal.pbio.3001900 (PMC9721491; doi:10.1371/journal.pbio.3001900)
Supplement: S1 Fig — (A) The analysis of sequencing results for the OP9 FABP4-KO clone used in Fig 1. (B) Measurements of FABP5 levels by immunocytochemistry show that FABP5 is up-regulated in FABP4 knockout cells. Bar plots are mean +/–SEM from 3 technical replicates with approximately 2,000 cells per replicate. (C) Validation of the efficiency of FABP5 siRNA in OP9 preadipocytes assessed by carrying out immunocytochemistry. Bar plots show mean +/–SEM from 3 technical replicates with approximately 5,000 cells per replicate. (B, C) Unpaired t test, **, p < 0.01; ***, p < 0.001. (D, E) Knockout of FABP4 and FABP5 impairs adipogenesis in OP9 preadipocyte cells induced to differentiate by the standard DMI protocol. The addition of 1 μm rosiglitazone rescues the loss of adipogenesis in FABP4-KO OP9 cells and in FABP4-KO OP9 cells transfected with FABP5 siRNA. Scale bar is 90 μm. The data underlying the graphs in the figure can be found in https://zenodo.org/record/7012787#.Y2I5I0zP3b0. (PDF) [file pbio.3001900.s001.pdf]

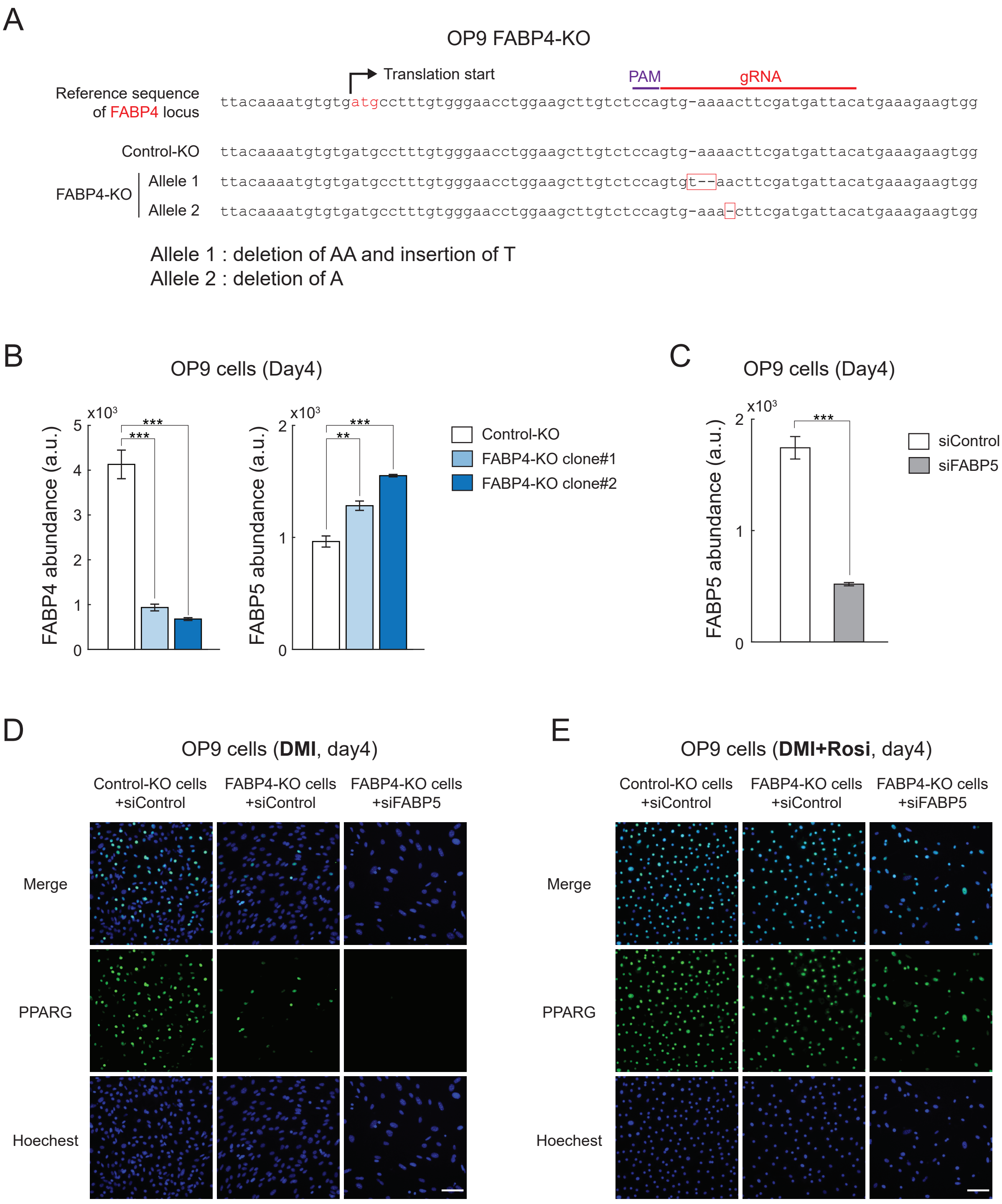

Figure S1. Additional experiments supporting that FABP4 regulates PPARG expression and adipogenesis in OP9 cells.
(A) The analysis of sequencing results for the OP9 FABP4-KO clone used in Figure 1.
(B) Measurements of FABP5 levels by immunocytochemistry show that FABP5 is upregulated in FABP4 knockout cells. Bar plots are mean +/– SEM from 3 technical replicates with approximately 2,000 cells per replicate.
(C) Validation of the efficiency of FABP5 siRNA in OP9 preadipocytes assessed by carrying out immunocytochemistry. Bar plots show mean +/– SEM from 3 technical replicates with approximately 5,000 cells per replicate.
(B, C) Unpaired t-test, **, $p < 0.01$; ***, $p < 0.001$.
(D, E) Knockout of FABP4 and FABP5 impairs adipogenesis in OP9 preadipocyte cells induced to differentiate by the standard DMI protocol. The addition of 1 μM Rosiglitazone rescues the loss of adipogenesis in FABP4-KO OP9 cells and in FABP4-KO OP9 cells transfected with FABP5 siRNA. Scale bar is 90 μm.
