# Supplementary material for: Early enforcement of cell identity by a functional component of the terminally differentiated state
Source: PLoS Biol. 2022 Dec 5;20(12):e3001900. doi: 10.1371/journal.pbio.3001900 (PMC9721491; doi:10.1371/journal.pbio.3001900)
Supplement: S2 Fig — (A, B) The analysis of sequencing results for the 3T3-F442A FABP4-KO (A) and FABP4/FABP5 DBKO (B) clones used in Fig 1. (C, D) Knockout of FABP4 and FABP5 impairs adipogenesis in 3T3-FF42A preadipocyte cells induced to differentiate by the standard protocol of adding insulin. The addition of 1 μm rosiglitazone rescues the loss of adipogenesis in FABP4-KO and FABP4/FABP5 DBKO 3T3-F442A cells. Scale bar is 30 μm. (PDF) [file pbio.3001900.s002.pdf]

A

3T3-F442A FABP4-KO

Translation start

PAM gRNA

Reference sequence of FABP4 locus ttacaaaatgtgtgatgcctttgtgggaacctggaagcttgtctccagtgaaaa-cttcgatgattacatgaaagaagtgg

Control-KO ttacaaaatgtgtgatgcctttgtgggaacctggaagcttgtctccagtgaaaa-cttcgatgattacatgaaagaagtgg

FABP4-KO
Allele 1 ttacaaaatgtgtgatgcctttgtgggaacctggaagcttgtctccagtgaaaaacttcgatgattacatgaaagaagtgg
Allele 2 ttacaaaatgtgtgatgcctttgtgggaacctggaagcttgtctccagtgaa---cttcgatgattacatgaaagaagtgg

Allele 1 : insertion of A
Allele 2 : deletion of AA

B

3T3-F442A FABP4/FABP5 DBKO

Translation start

PAM gRNA

Reference sequence of FABP4 locus ttacaaaatgtgtgatgcctttgtgggaacctggaagcttgtctccagtgaaaacttcgatgattacatgaaagaagtgg

Control-KO ttacaaaatgtgtgatgcctttgtgggaacctggaagcttgtctccagtgaaaacttcgatgattacatgaaagaagtgg

FABP4/FABP5 DBKO
Allele 1 ttacaaaatgtgtgatgcctttgtgggaacctggaagcttgtctccagtga-----tcgatgattacatgaaagaagtgg
Allele 2 ttacaaaatgtgtgatgcctttgtgggaacctggaagcttgtctccagtga-----tcgatgattacatgaaagaagtgg

Allele 1 & 2 : deletion of AAACT

Translation start

PAM gRNA

Reference sequence of FABP5 locus ctctccctcccaccatggccagtcttaaggatctcgaagggaagtggcgcctgatggaaagccacggctttgaggagtac

Control-KO ctctccctcccaccatggccagtcttaaggatctcgaagggaagtggcgcctgatggaaagccacggctttgaggagtac

FABP4/FABP5 DBKO
Allele 1 ctctccctcccaccatggccagtct-aaggatctcgaagggaagtggcgcctgatggaaagccacggctttgaggagtac
Allele 2 ctctccctcccaccatggccagtct-aaggatctcgaagggaagtggcgcctgatggaaagccacggctttgaggagtac

Allele 1 & 2 : deletion of T

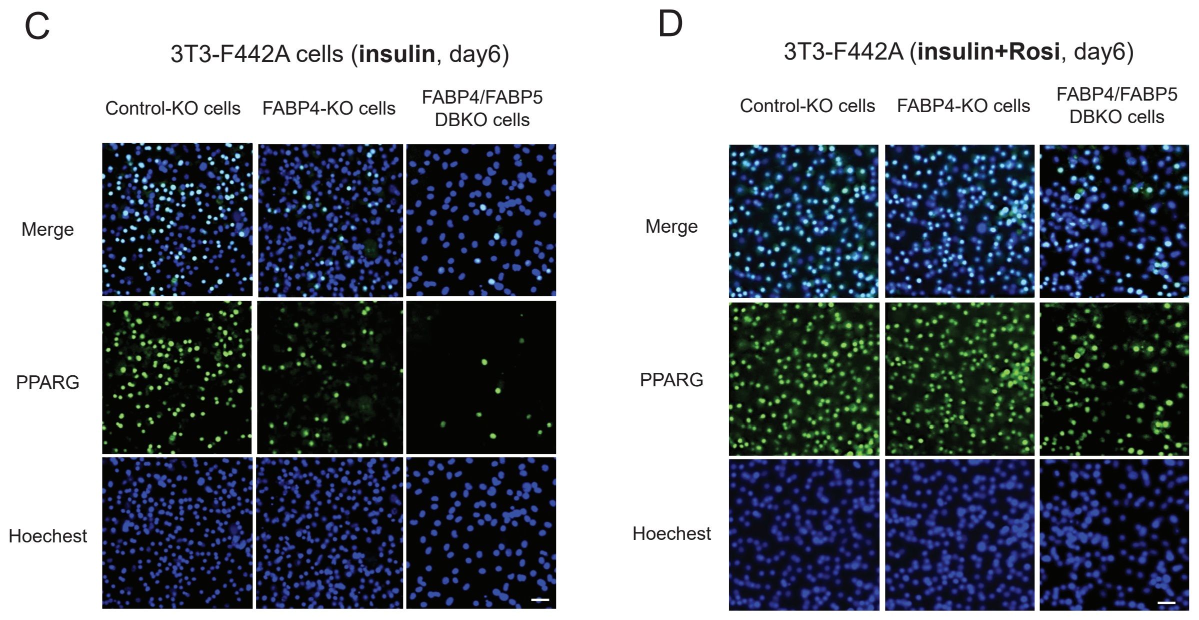

Figure S2. Additional experiments supporting that FABP4 regulates PPARG expression and adipogenesis in 3T3-F442A cells.
(A, B) The analysis of sequencing results for the 3T3-F442A FABP4-KO (A) and FABP4/FABP5 DBKO (B) clones used in Figure 1. (C, D) Knockout of FABP4 and FABP5 impairs adipogenesis in 3T3-FF42A preadipocyte cells induced to differentiate by the standard protocol of adding insulin. The addition of 1 μM Rosiglitazone rescues the loss of adipogenesis in FABP4-KO and FABP4/FABP5 DBKO 3T3-F442A cells. Scale bar is 30 μm.
