# Supplementary material for: Early enforcement of cell identity by a functional component of the terminally differentiated state
Source: PLoS Biol. 2022 Dec 5;20(12):e3001900. doi: 10.1371/journal.pbio.3001900 (PMC9721491; doi:10.1371/journal.pbio.3001900)
Supplement: S3 Fig — In addition to the clones for the 3T3-F442A Control-KO, FABP4-KO, and FABP4/FABP5 DBKO cells as shown in Figs 1 and 6 and S2, a second clone was validated and tested for each cell lines in the same manner. Cells were grown to 2 days post-confluence and were induced to differentiate by addition of insulin (A–C) or insulin + 1 μm rosiglitazone (D). Cells were assayed at day 6 post-induction. (A) The loss of FABP4 and FABP5 proteins was validated by western blot analysis. β-actin was used as a loading control. (B) The extent of adipogenesis was assessed by Oil Red O staining for intracellular lipid accumulation. Scale bar, 100 μm. (C, D) The percent of differentiated cells were assessed by counting cells that above the PPARG threshold and dividing by the total number of cells (see Methods). Bar plots show mean +/–SEM from 3 technical replicates with approximately 1,000 cells per replicate. Data is representative of 3 independent experiments. The data underlying the graphs in the figure can be found in https://zenodo.org/record/7012787#.Y2I5I0zP3b0. (PDF) [file pbio.3001900.s003.pdf]

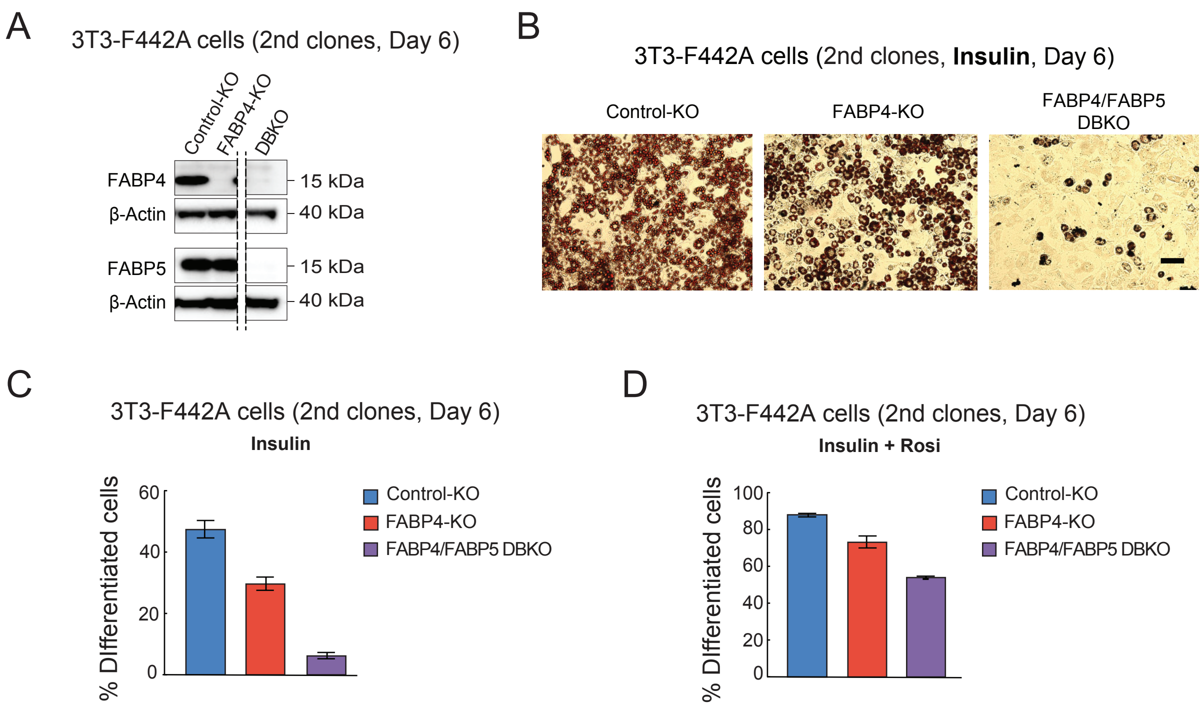

Figure S3. Data for the second clones of 3T3-F442A Control-KO, FABP4-KO and FABP4/FABP5 DBKO cells.
In addition to the clones for the 3T3-F442A Control-KO, FABP4-KO and FABP4/FABP5 DBKO cells as shown in Figure 1, 6 and S2, a second clone was validated and tested for each cell lines in the same manner. Cells were grown to 2 days post-confluence and were induced to differentiate by addition of insulin (A-C) or insulin + 1 μM Rosiglitazone (D). Cells were assayed at day 6 post-induction.
(A) The loss of FABP4 and FABP5 proteins was validated by western blot analysis. β-actin was used as a loading control.
(B) The extent of adipogenesis was assessed by Oil Red O staining for intracellular lipid accumulation. Scale bar, 100 μm.
(C, D) The percent of differentiated cells were assessed by counting cells that above the PPARG threshold and dividing by the total number of cells (See Methods). Bar plots show mean +/– SEM from three technical replicates with approximately 1,000 cells per replicate. Data is representative of 3 independent experiments.
