# Supplementary material for: Early enforcement of cell identity by a functional component of the terminally differentiated state
Source: PLoS Biol. 2022 Dec 5;20(12):e3001900. doi: 10.1371/journal.pbio.3001900 (PMC9721491; doi:10.1371/journal.pbio.3001900)
Supplement: S4 Fig — C3H10T1/2-CRISPRa-SAM cells were transfected with either empty vector (Control) or guide RNA targeting the FABP4 promoter region (FABP4-OE); 48 hours later, the cells were induced to differentiate using 96-hour DMI protocol as in Fig 1B. (A) qRT-PCR to measure PPARG and Adiponectin (AdipoQ) expression. Data are normalized to 18s expression. Three biological replicates were used. A Student t test, 2 tail, type 2 was applied for statistical analysis. Values represent means ± SEM. ns, p > 0.05, ****p < 0.0001. (B) Immunocytochemistry to assess PPARG expression (red), lipid accumulation using Bodipy (green), and Hoechst to mark the nuclei (blue). Scale bar, 50 μm. The data underlying the graphs in the figure can be found in https://zenodo.org/record/7012787#.Y2I5I0zP3b0. (PDF) [file pbio.3001900.s004.pdf]

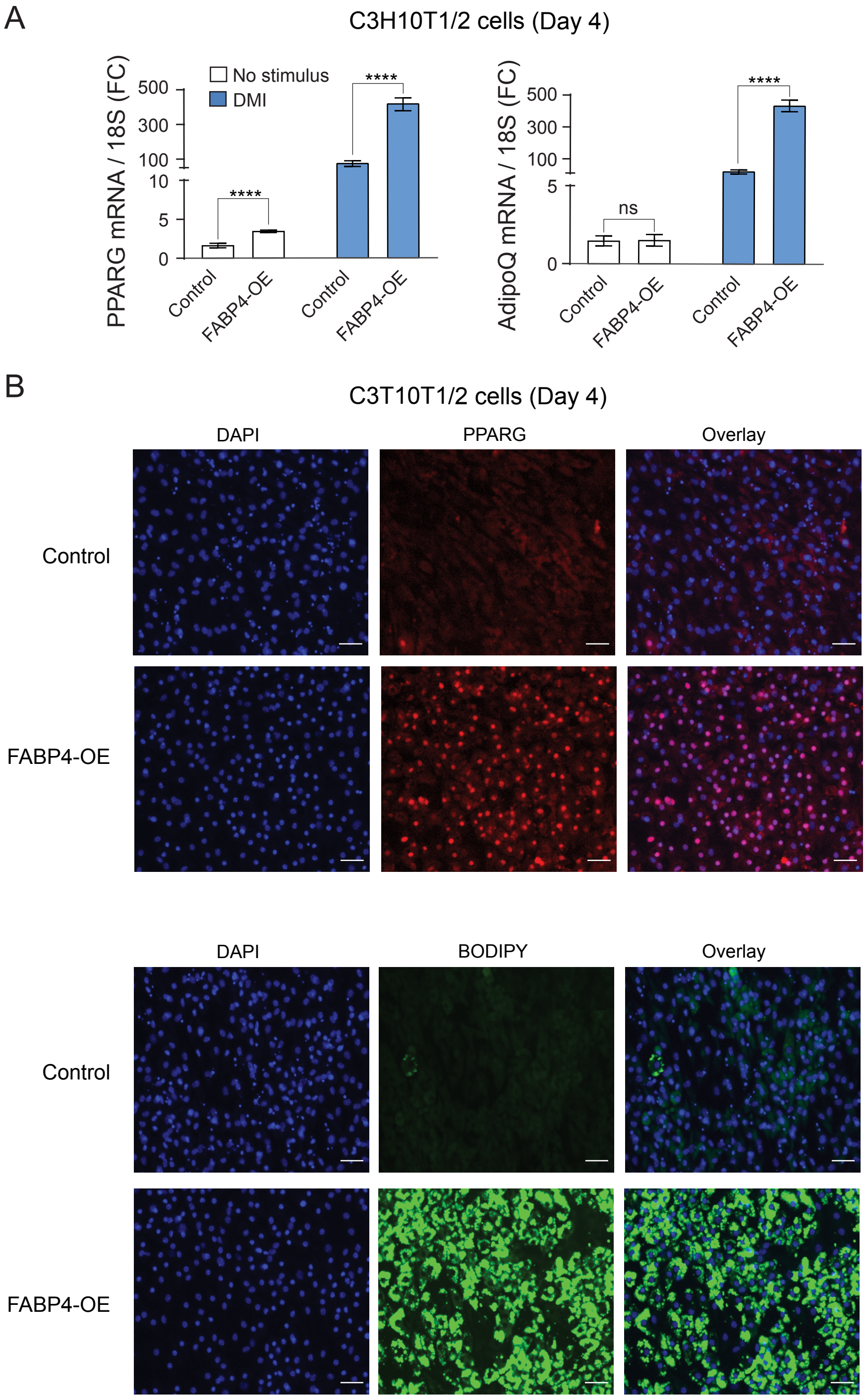

Figure S4. Increasing FABP4 expression by CRISPRa increases PPARG expression.
C3H10T1/2-CRISPRa-SAM cells were transfected with either empty vector (Control) or guide RNA targeting the FABP4 promoter region (FABP4-OE). 48 hours later, the cells were induced to differentiate using 96-hour DMI protocol as in Figure 1B.
(A) qRT-PCR to measure PPARG and Adiponectin (AdipoQ) expression. Data are normalized to 18s expression. Three biological replicates were used. A student T test, 2 tail, type 2 was applied for statistical analysis. Values represent means ± SEM. ns, $p>0.05$, ****$p<0.0001$.
(B) Immunocytochemistry to assess PPARG expression (red), lipid accumulation using Bodipy (green) and Hoechst to mark the nuclei (blue). Scale bar, 50 μM.
