# Supplementary figures and images for: Early enforcement of cell identity by a functional component of the terminally differentiated state
Source: PLoS Biol. 2022 Dec 5;20(12):e3001900. doi: 10.1371/journal.pbio.3001900 (PMC9721491; doi:10.1371/journal.pbio.3001900)

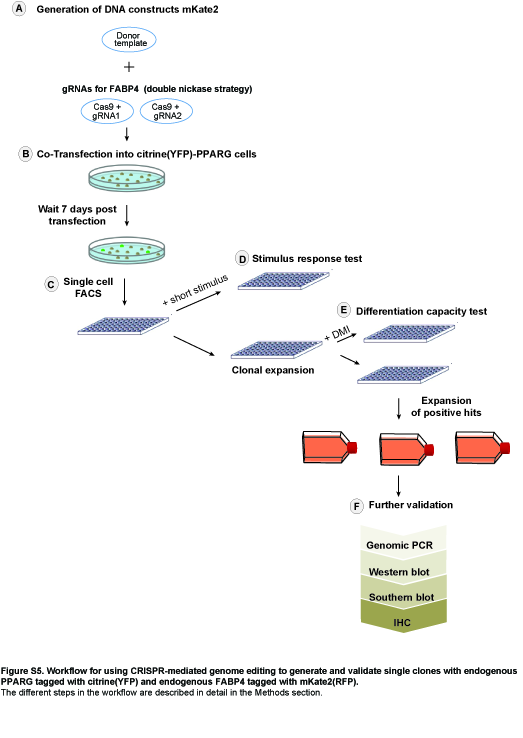

Supplement: S5 Fig — The different steps in the workflow are described in detail in the Methods section. (TIF) [file pbio.3001900.s005.tif]
