# Supplementary material for: Early enforcement of cell identity by a functional component of the terminally differentiated state
Source: PLoS Biol. 2022 Dec 5;20(12):e3001900. doi: 10.1371/journal.pbio.3001900 (PMC9721491; doi:10.1371/journal.pbio.3001900)
Supplement: S6 Fig — The different steps of the validation procedure are detailed in the Methods section. The data underlying the graphs in the figure can be found in https://zenodo.org/record/7012787#.Y2I5I0zP3b0. (PDF) [file pbio.3001900.s006.pdf]

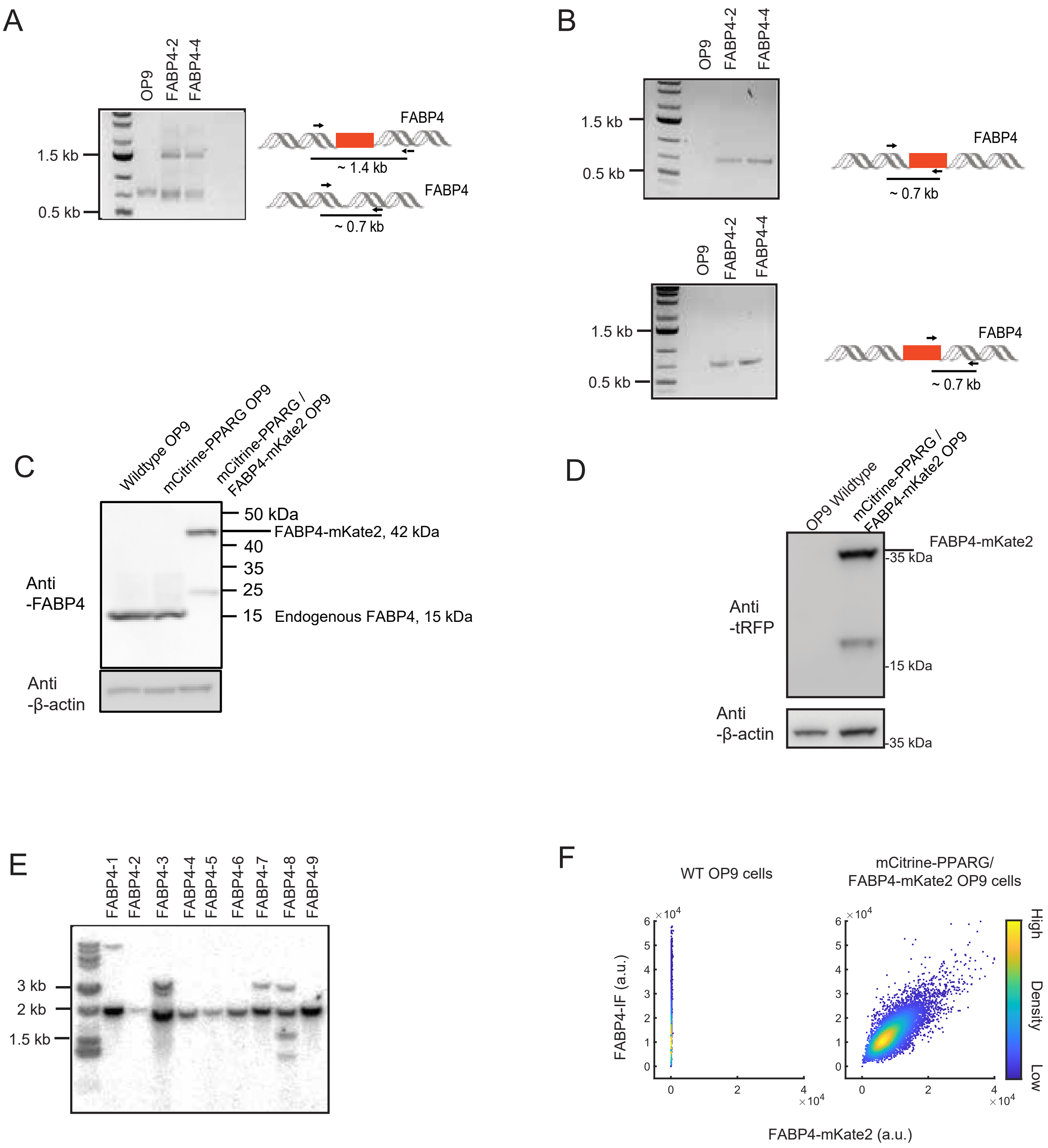

Figure S6. Validation of FABP4-mKate2(RFP) OP9 cell clones.
The different steps of the validation procedure are detailed in the Methods section.
