# Supplementary material for: Early enforcement of cell identity by a functional component of the terminally differentiated state
Source: PLoS Biol. 2022 Dec 5;20(12):e3001900. doi: 10.1371/journal.pbio.3001900 (PMC9721491; doi:10.1371/journal.pbio.3001900)
Supplement: S7 Fig — To determine the timing at which the PPARG dynamics in siRNA knockdown cells start to become diverged from that in negative control cells, we calculated the p-value by Kruskal–Wallis test at each time point. By plotting logarithm of p-value versus time, we found −20 could be a suitable value as the threshold. Then, we search for the time point at which the logarithm of p-value become, at the first time, less than −20, and use this very time point (red dashed line, indicated as separated time) as the timing from which the PPARG dynamics between knockdown cells and negative control cells start to get remarkably diverged. The data underlying the graphs in the figure can be found in https://zenodo.org/record/7012787#.Y2I5I0zP3b0. (PDF) [file pbio.3001900.s007.pdf]

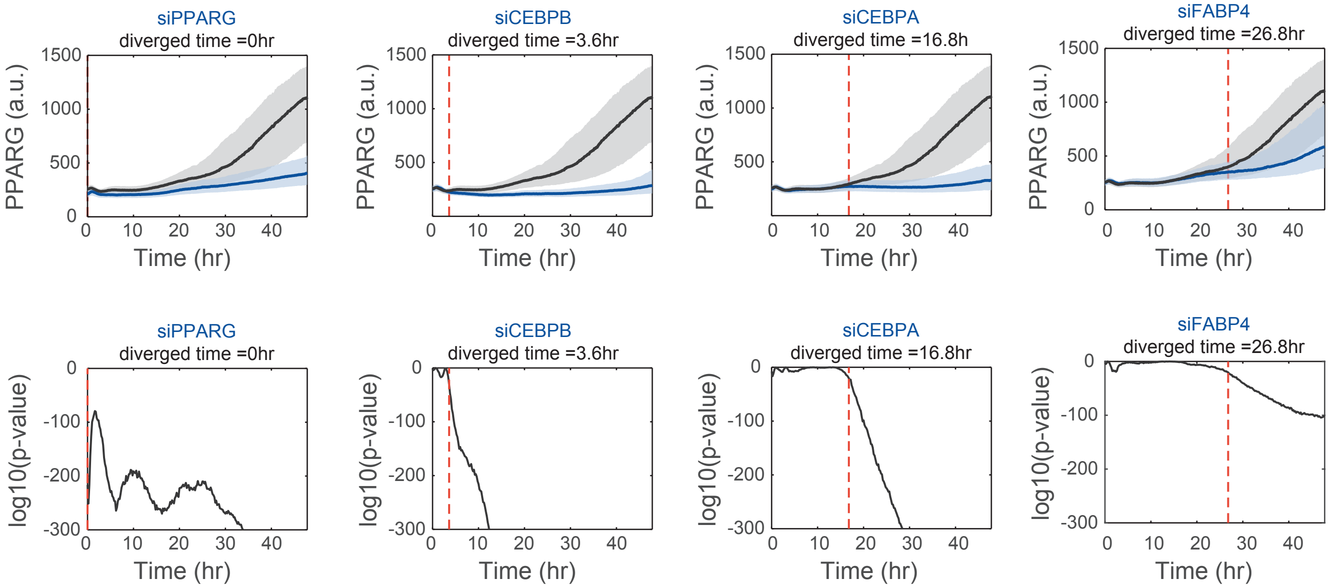

Figure S7. Probability analysis to determine the timing of feedback engagement.
To determine the timing at which the PPARG dynamics in siRNA knockdown cells start to become diverged from that in negative control cells, we calculated the p-value by Kruskal-Wallis test at each time point. By plotting logarithm of p-value versus time, we found -20 could be a suitable value as the threshold. Then we search for the time point at which the logarithm of p-value become, at the first time, less than -20, and use this very time point (red dashed line, indicated as separated time) as the timing from which the PPARG dynamics between knockdown cells and negative control cells start to get remarkably diverged.
