# Supplementary material for: Early enforcement of cell identity by a functional component of the terminally differentiated state
Source: PLoS Biol. 2022 Dec 5;20(12):e3001900. doi: 10.1371/journal.pbio.3001900 (PMC9721491; doi:10.1371/journal.pbio.3001900)
Supplement: S8 Fig — Citrine-PPARG and FABP4-mKate2 cells were transfected with FABP4-targeted or control siRNA and were imaged while being induced to differentiate by the standard DMI protocol. Plots are the median of FABP4 abundance from about 8,000 cells. The data underlying the graphs in the figure can be found in https://zenodo.org/record/7012787#.Y2I5I0zP3b0. (PDF) [file pbio.3001900.s008.pdf]

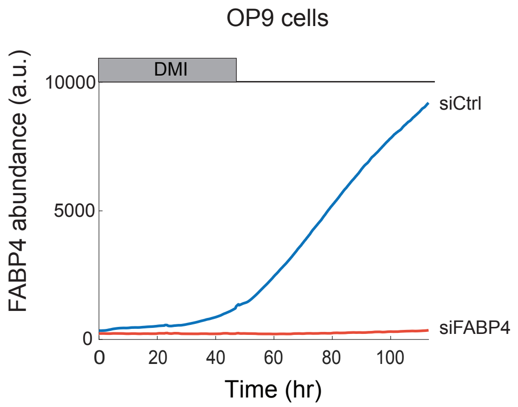

Figure S8. Validation of FABP4 knockdown in OP9 cells.
Citrine-PPARG and FABP4-mKate2 cells were transfected with FABP4-targeted or control siRNA and were imaged while being induced to differentiate by the standard DMI protocol. Plots are the median of FABP4 abundance from about 8,000 cells.
