# Supplementary material for: Early enforcement of cell identity by a functional component of the terminally differentiated state
Source: PLoS Biol. 2022 Dec 5;20(12):e3001900. doi: 10.1371/journal.pbio.3001900 (PMC9721491; doi:10.1371/journal.pbio.3001900)
Supplement: S1 Table — (PDF) [file pbio.3001900.s009.pdf]

**Name**	**Source**	**Identifier**
pX335-U6-Chimeric_BB-CBh-hSpCas9n(D10A)	Addgene #42335	Addgene
pX335-U6-Chimeric_BB-CBh-hSpCas9n(D10A)-FABP4-1<br><br>pX335-U6-Chimeric_BB-CBh-hSpCas9n(D10A)-FABP4-2	This paper	These 2 constructs each introduce a FABP4 guide RNA together with a double nickase Cas9 in order to “nick” the DNA at the C-terminal of FABP4 in order to insert mKate2(RFP).
ENTR1A-FABP4-mKate2(RFP)-donor vector	This paper	Construct to introduce mKate2(RFP) at the C-terminal of FABP4
pSpCas9n(BB)-2A-GFP (PX461)	Addgene #48140	Addgene
pSpCas9(BB)-2A-miRFP670	Addgene #91854	Addgene
pSpCas9n(BB)-2A-GFP (PX461)-FABP4	This paper	Construct to KO FABP4 using CRISPR-Cas9
pSpCas9n(BB)-2A-miRFP670 (PX461)-FABP5	This paper	Construct to KO FABP5 using CRISPR-Cas9
pCMV-YFP	This paper	Construct to overexpress YFP
pCMV-FABP4	This paper	Construct to overexpress FABP4

S1_Table: Plasmids
