# Supplementary material for: Early enforcement of cell identity by a functional component of the terminally differentiated state
Source: PLoS Biol. 2022 Dec 5;20(12):e3001900. doi: 10.1371/journal.pbio.3001900 (PMC9721491; doi:10.1371/journal.pbio.3001900)
Supplement: S2 Table — Guide sequences are targeted to the FABP4 C-terminal. The underlined and italicized nucleotides denote the overhang for ligation of the oligonucleotide duplex into the px335 guide sequence insertion site. (PDF) [file pbio.3001900.s010.pdf]

Target	Strand	Oligonucleotide sequence<br>(5' to 3')
FABP4_Cterm_1	Top	*CACCGCATAACACATTCCTAGACAC*
FABP4_Cterm_1	Bottom	*AAACGTGTCTAGGAATGTGTTATGC*
FABP4_Cterm_2	Top	*CACCGTATGAAAGGGCATGAGCCAA*
FABP4_Cterm_2	Bottom	*AAACTTGGCTCATGCCCTTTCATAC*

S2_Table: Oligonucleotide sequences used to insert sgRNA sequences into the px335. Guide sequences are targeted to the FABP4 C-terminal. The underlined and italicized nucleotides denote the overhang for ligation of the oligonucleotide duplex into the px335 guide sequence insertion site.
