# Supplementary material for: Early enforcement of cell identity by a functional component of the terminally differentiated state
Source: PLoS Biol. 2022 Dec 5;20(12):e3001900. doi: 10.1371/journal.pbio.3001900 (PMC9721491; doi:10.1371/journal.pbio.3001900)
Supplement: S3 Table — (PDF) [file pbio.3001900.s011.pdf]

**Target**	**Strand**	**Oligonucleotide sequence (5' to 3')**
FABP4	Top	*CACCgGTAATCATCGAAGTTTTCAC*
FABP5	Bottom	*CACCgCCCTTCGAGATCCTTAAGAC*

S3_Table: Oligonucleotide sequences used for CRISPR KO of FABP4 or FABP5
