# Supplementary material for: Early enforcement of cell identity by a functional component of the terminally differentiated state
Source: PLoS Biol. 2022 Dec 5;20(12):e3001900. doi: 10.1371/journal.pbio.3001900 (PMC9721491; doi:10.1371/journal.pbio.3001900)
Supplement: S4 Table — (PDF) [file pbio.3001900.s012.pdf]

**Primer Name**	**Template**	**Primer sequence (5' to 3')**
FABP4_homology_region1_FWD	OP9 genomic DNA	AACCAATTCAGTCGACTGCTGTGCC CACAGAGCATCATAAC
FABP4_homology_region1_REV	OP9 genomic DNA	TCGCTCACTCCTCCTCCTGCCCTTT CATAAACTCTTGTGGAAGTCAC
FABP4_homology_region2_FWD	OP9 genomic DNA	ACTGGGGCACAGATGAGCCAAAGG AAGAGGCCTGGA
FABP4_homology_region2_FWD	OP9 genomic DNA	TCGAGTGCGGCCGCGACTCTCTTGA GCATTCAGCCT
Fabp4_mKate2_FWD	mKate2 plasmid	TGAAAGGGCAGGAGGAGGAGTGAG CGAGCTGATTAAGGAG
Fabp4_mKate2_REV	mKate2 plasmid	CCTCTTCCTTTGGCTCATCTGTGCC CCAGTTTGCTAGGG

S4_Table: Primers used for PCR amplification of fragments that were joined by Gibson assembly to create donor vectors to insert Citrine at the C-terminal of FABP4 via homologous recombination.
