# Supplementary material for: Early enforcement of cell identity by a functional component of the terminally differentiated state
Source: PLoS Biol. 2022 Dec 5;20(12):e3001900. doi: 10.1371/journal.pbio.3001900 (PMC9721491; doi:10.1371/journal.pbio.3001900)
Supplement: S5 Table — (PDF) [file pbio.3001900.s013.pdf]

**Assay**	**Primer sequence (5' to 3')**	**Amplicon (bp)**
genotyping FABP4 Knockout clones	**FWD**: CAGGGTCTGGTCATGAAGG	~600
	**REV**: ACATGATTTGTTTTCTAAGAGGGGCA	
genotyping FABP4 mKate2 clones	**FWD:** TCTTCCTGGTCTTTGTACCACCCT	**736** (wt allele)
	**REV:** CAGGGCAGAAACAAAGCTTCATG	**1429**(knock-in allele)

S5_Table: Primers used for genomic PCR analysis of the FABP4 CRISPR clones.
