# Supplementary material for: Early enforcement of cell identity by a functional component of the terminally differentiated state
Source: PLoS Biol. 2022 Dec 5;20(12):e3001900. doi: 10.1371/journal.pbio.3001900 (PMC9721491; doi:10.1371/journal.pbio.3001900)
Supplement: S6 Table — (PDF) [file pbio.3001900.s014.pdf]

| Assay | Primer sequence (5' to 3') | Amplicon (bp) |
| --- | --- | --- |
| seq. 1 FABP4 mKate2 clones | **FWD:**TCTTCCTGGTCTTTGTACCACCCT | - (wt allele) 712 (knock-in allele) |
| | **REV:** TCCCAGCCGAGTGTTTTCTTCT | |
| seq. 2 FABP4 mKate2 clones | **FWD:**AGAAGAAAACACTCGGCTGGGA | - (wt allele) 739 (knock-in allele) |
| | **REV:** CAGGGCAGAAACAAAGCTTCATG | |

**S6_Table: Primers used for genomic PCR analysis to verify the fluorophore integration sites of the FABP4 tagged clones.**
