# Supplementary material for: Early enforcement of cell identity by a functional component of the terminally differentiated state
Source: PLoS Biol. 2022 Dec 5;20(12):e3001900. doi: 10.1371/journal.pbio.3001900 (PMC9721491; doi:10.1371/journal.pbio.3001900)
Supplement: S7 Table — (PDF) [file pbio.3001900.s015.pdf]

**Primer Name**	**Primer sequence (5' to 3')**
mkate2_probe_FWD	CAACCACCACTTCAAGTGCACA
mkate2_probe_REV	CTTGAGGTTCTTAGCGGGTTTCTTG

S7_Table: Primers used for the PCR amplification of a 504 bp probe directed towards Citrine and 505 bp probe directed towards mKate2.
