# Supplementary material for: Early enforcement of cell identity by a functional component of the terminally differentiated state
Source: PLoS Biol. 2022 Dec 5;20(12):e3001900. doi: 10.1371/journal.pbio.3001900 (PMC9721491; doi:10.1371/journal.pbio.3001900)
Supplement: S8 Table — (PDF) [file pbio.3001900.s016.pdf]

Gene	Forward	Reverse
Adipoq	TGT TCC TCT TAA TCC TGC CCA	CCA ACC TGC ACA AGT TCC CTT
Fabp4	AAG GTG AAG AGC ATC ATA ACC CT	TCA CGC CTT TCA TAA CAC ATT CC
Pparγ2	TCG CTG ATG CAC TGC CTA TG	GAG AGG TCC ACA GAG CTG ATT
18s	AGTCCCTGCCCTTTGTACACA	GATCCGAGGGCCTCACTAAAC

S8_Table: List of primer sequences used for quantitative PCR-based gene expression analysis.
