# Supplementary material for: Early enforcement of cell identity by a functional component of the terminally differentiated state
Source: PLoS Biol. 2022 Dec 5;20(12):e3001900. doi: 10.1371/journal.pbio.3001900 (PMC9721491; doi:10.1371/journal.pbio.3001900)
Supplement: S9 Table — (PDF) [file pbio.3001900.s017.pdf]

Name	Description	Source
FABP4/PPARG dual tagged OP9 cells	citrine(YFP)-PPARG and FABP4mKate2(RFP) OP9 cells	This paper
CEBPA tagged OP9 cells	citrine(YFP)-CEBPA OP9 cells	Ref. 1
FABP4 tagged OP9 cells	FABP4-citrine(YFP) OP9 cells	Ref. 1
FABP4-KO 3T3-F442A cells	CRISPR knockout FABP4 in 3T3F442A cells	This paper
FABP4/FABP5 double knockout 3T3-F442A cells	CRISPR knockout both FABP5 and FABP4 in 3T3-F442A cells	This paper
FABP4-KO OP9 cells	CRISPR knockout FABP4 in OP9 cells	This paper

S9_Table: Cell lines.

Reference

1. Zhang Z-B, Sinha J, Bahrami-Nejad Z, Teruel MN. The circadian clock mediates daily bursts of cell differentiation by periodically restricting cell differentiation commitment. Proc Natl Acad Sci U S A. 2022;119: e2204470119. doi:10.1073/pnas.2204470119.
