# Supplementary material for: Early enforcement of cell identity by a functional component of the terminally differentiated state
Source: PLoS Biol. 2022 Dec 5;20(12):e3001900. doi: 10.1371/journal.pbio.3001900 (PMC9721491; doi:10.1371/journal.pbio.3001900)
Supplement: S10 Table — (PDF) [file pbio.3001900.s018.pdf]

REAGENT or RESOURCE	SOURCE	IDENTIFIER
**Antibodies**		
Mouse monoclonal anti-PPARgamma (E-8)	Santa Cruz Biotechnology	Cat# sc-7273
Rabbit polyclonal anti-PPARgamma2	Abcam	Cat# ab45036
Rabbit polyclonal anti-FABP4	Abcam	Cat# ab13979
Goat polyclonal anti-FABP4	R&D	Cat# AF1443
Rabbit polyclonal anti-GFP	Abcam	Cat# ab290
Rabbit polyclonal anti-tRFP	Evrogen	Cat# EVN-AB233C100
Mouse monoclonal b-actin antibody	Santa Cruz Biotechnology	Cat #sc-47778
Rabbit (D1A7T) monoclonal anti-FABP5	Cell Signaling	Cat #39926
Horseradish peroxidase (HRP)-conjugated anti-mouse	Cell Signaling	Cat #7076
Horseradish peroxidase (HRP)-conjugated anti-rabbit	Cell Signaling	Cat #7074
Goat anti-rabbit IgG (H+L) cross-adsorbed secondary antibody, Alexa Fluor 514	Invitrogen	Cat #A31558
Goat anti-mouse IgG (H+L) cross-adsorbed secondary antibody, Alexa Fluor 594	Invitrogen	Cat #A11032
Donkey anti-mouse IgG (H+L) cross-adsorbed secondary antibody, Alexa Fluor 647	Invitrogen	Cat #A31571
**Chemicals, Peptides, and Recombinant Proteins**		
IBMX	Sigma-Aldrich	Cat #7018
Dexamethasone	Sigma-Aldrich	Cat #D1756
Insulin	Sigma-Aldrich	Cat # I6634
Bovine serum albumin	Sigma-Aldrich	Cat #7906
Rosiglitazone	Cayman	Cat #7906
Linoleic acid	Sigma-Aldrich	Cat #L9530
BODIPY	Invitrogen	Cat #D-3921
Hoechst 33342 (used like DAPI as a nuclear stain)	ThermoFisher	Cat #H3570
**Experimental Models: Cell Lines and Organisms**		
OP9 mouse stromal cell line	Wolins et al., 2006	N/A
3T3-F442A mouse preadipocyte cell line	Lab of Prof. Emeritus Howard Green, Harvard University	N/A
C3H10T1/2 mouse mesenchymal stem cell	ATCC	Clone 8, Cat #CCL226

Immune-deficient mouse-J:NU	Jackson Labs	Cat #007860
**siRNA**		
CEBPB siRNA	Dharmacon	Cat #L-043110-000005
PPARG siRNA	Dharmacon	Cat #L-040712-000005
FABP4 siRNA	Dharmacon	Cat #L-042923-010005
FABP4 siRNA	Qiagen	Cat #SI02695322
FABP5 siRNA	Dharmacon	Cat #L-043807-010005
Control siRNA	Dharmacon	Cat #D-001810-1005
Control siRNA	Qiagen	Cat #1027310
**Plasmids** (See S1_Table)		
**Oligonucleotides** (See S2-8_Tables)		
**Cell Lines** (See S9_Table)		

S10_Table: Key Resources Table.
