# Supplementary material for: Early enforcement of cell identity by a functional component of the terminally differentiated state
Source: PLoS Biol. 2022 Dec 5;20(12):e3001900. doi: 10.1371/journal.pbio.3001900 (PMC9721491; doi:10.1371/journal.pbio.3001900)
Supplement: S1 Raw images — (PDF) [file pbio.3001900.s019.pdf]

<u>Fig 1E related western blots (Chemiluminescence imaging)</u>

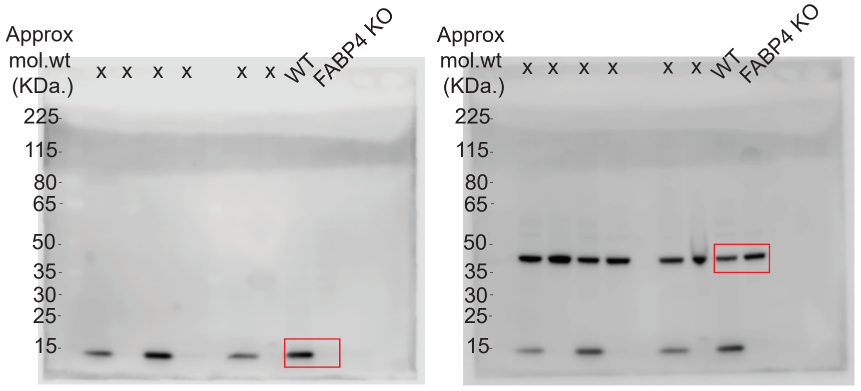

Anti- FABP4

Anti- β-actin

<u>Fig 1G (clone 1) and Fig S3A (clone 2) related western blots (Chemiluminescence imaging)</u>

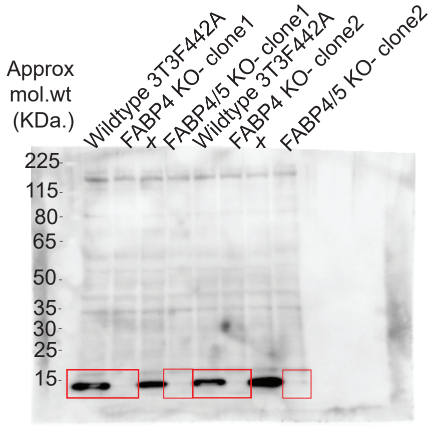

Anti- FABP4

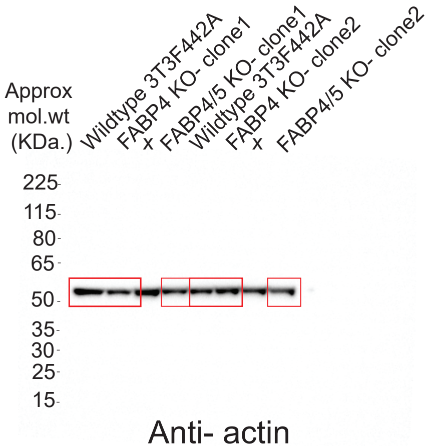

Anti- actin

Lorem ipsum

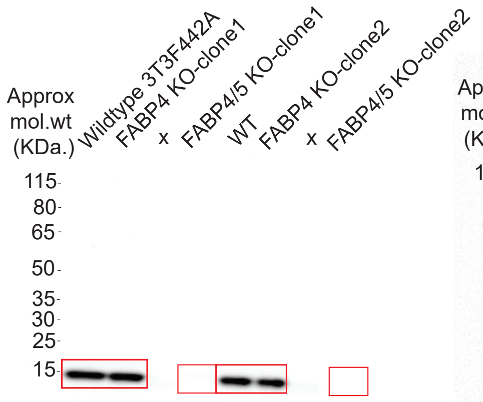

Anti- FABP5

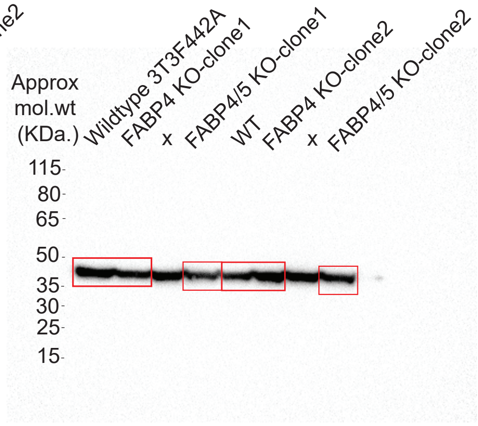

Anti- actin

Note: lanes marked ‘x’ are either similar replicate samples or samples not relavant to the figure. Boxed regions/lanes were used in the article figure panels

Fig S6 A nd B related DNA agarose gels (Fluorescence Imaging)

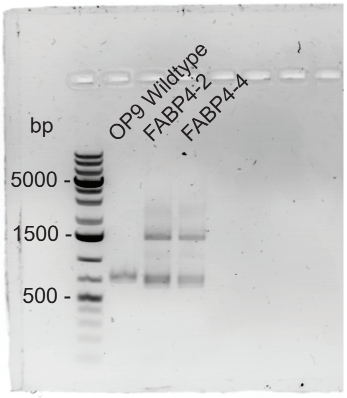

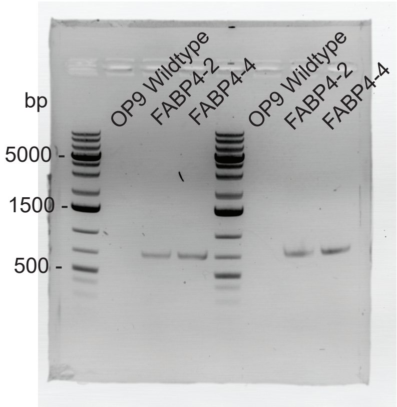

Fig S6C related westen blot (Chemiluminescence Imaging)

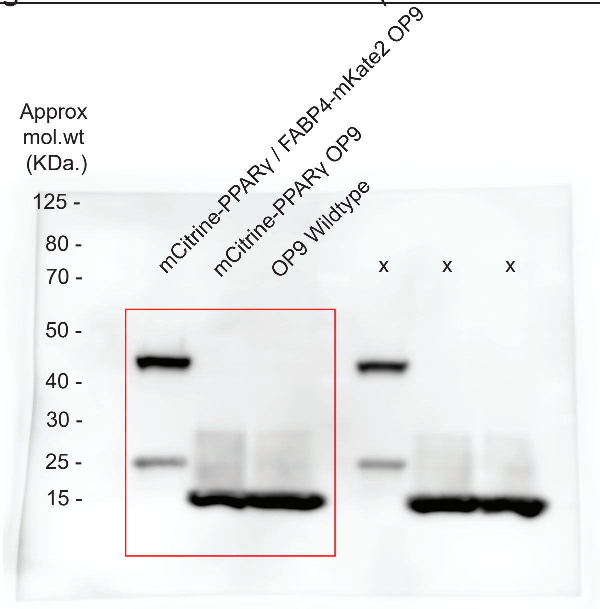

Anti- FABP4

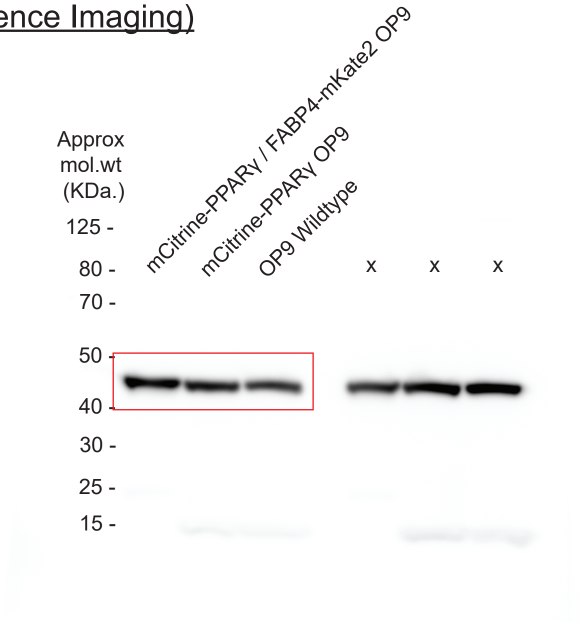

Anti- β-actin

Fig S6D related Western blot (Infared Fluorescence and Chemiluminescence Imaging)

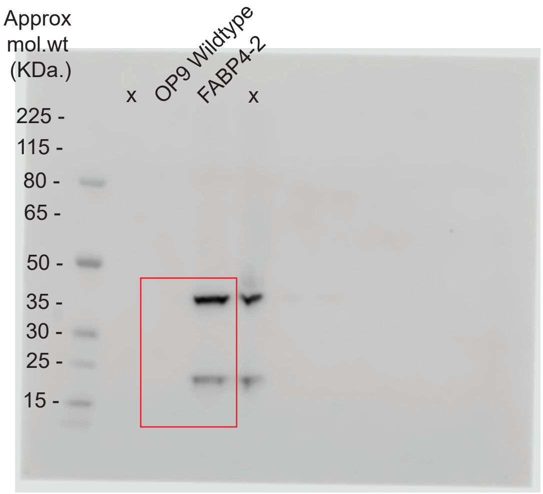

Anti- RFP

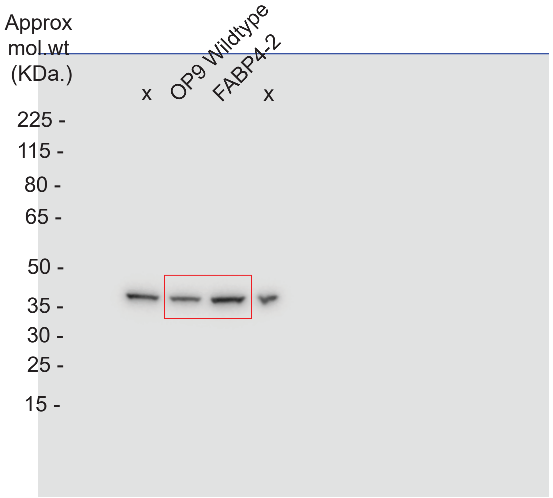

Anti- β-actin

Fig S6E related Southern blot (^{32}P- Phosphor Imaging)

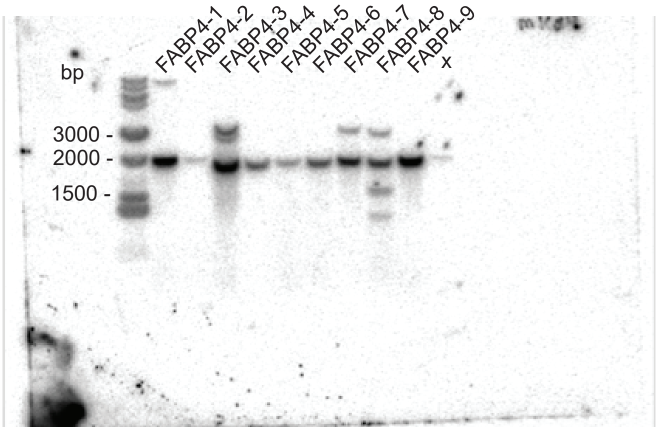

Expected band size: 2000 bp
