# Supplementary material for: Early enforcement of cell identity by a functional component of the terminally differentiated state
Source: PLoS Biol. 2022 Dec 5;20(12):e3001900. doi: 10.1371/journal.pbio.3001900 (PMC9721491; doi:10.1371/journal.pbio.3001900)
Supplement: Attachment — Submitted filename: 2022-08-08-response-to-reviewers.pdf [file pbio.3001900.s020.pdf]

RESPONSE TO REVIEWERS' COMMENTS

Reviewer 1:

In this manuscript, the authors evaluate the interesting concept of positive feedback loops in cellular differentiation and utilize the preadipocyte differentiation system as an experimental tool. They posit that just as negative feedback biases differentiation events towards different fates, positive feedback may drive a differentiation event towards a terminally differentiated state. Case in point for this manuscript is the relationship between PPARgamma and adipogenesis. The authors argue for two feedback loops, one early loop involving CEBPalpha and a second later loop involving FABP4 and PPARg. The experiments are well done, thought provoking and largely agree with their hypothesis. During review, the following questions arose:

1. FABP4 is also expressed normally in macrophages and brain microglial cells. Would the authors argue that the same second phase regulatory system exist in those cells? This should be commented on in the discussion.

It is possible that the second phase regulatory system exists also in macrophages since there is evidence, FABP4 and PPARG are in a positive feedback loop both in macrophages [1] and in monocytes, which differentiate into macrophages [2]. We have now included this point in the Discussion section (Lines 400-402).

2. As reported by Hotamisligil and others, the FABP4 KO mouse exhibits normal adipogenesis and the authors of this manuscript would argue that FABP5 compensates. However, there are no experiments in this manuscript that demonstrate such compensation:

The reviewer raises a good point that we should have been clearer about that we carried out experiments to demonstrate the compensation of FABP5 when FABP4 is missing (Fig. S1B). We now make this clearer in the text, as well as described supporting studies from others:

(Lines 134-145) "FABP4-KO OP9 cells were strongly defective in increasing PPARG expression and differentiating, with only a small fraction of cells differentiating compared to control cells. We suspected that the residual differentiation in FABP4-KO cells could be due to compensation by FABP5. FABP5 is expressed at an approximately 100-fold lower concentration than FABP4 in adipocytes[3], but studies by us and others have shown that FABP5 can be upregulated to compensate to some extent for the loss of FABP4 in cells and mice (Fig. S1B)[4–6]. FABP5 does not bind to PPARG directly, but rather increases PPARG activity and adipogenesis indirectly, possibly through binding to PPARdelta which has been shown to potentiate PPARG activity [7,8]. To test whether FABP5 expression was responsible for the remaining small fraction of differentiated cells in the FABP4-KO cells, we carried siRNA-mediated knockdown of FABP and indeed adipogenesis was reduced to almost nothing (Fig 1C).

We further validated these findings in another commonly used preadipocyte cell system, 3T3-F442A cells [9]. We used CRISPR-mediated genome editing to knockout FABP4 or both FABP4 and FABP5 (Fig 1D). 3T3-F442A cells which had been subjected to the same FABP4 knockout protocol, but which did not harbor any FABP4 knockout were used as control cells. We plated the 3T3-F442A cells into 96-well plates and induced adipogenesis using the standard protocol in 3T3-F442A cells which is to add insulin[9]. Indeed, when FABP4 was knocked out, 3T3-F442A preadipocytes showed reduced differentiation and reduced lipid accumulation, as measured by PPARG staining and Oil Red-O staining, respectively (Fig 1E and 1F). A lack of lipid accumulation is indicative that most of the KO cells are not functional mature adipocytes. As in the case of the OP9 preadipocyte cells (Fig. 1C), some differentiation was observed in the FABP4-KO cells, but this differentiation was abolished when FABP5 was also knocked out (Fig 1E), supporting that FABP5 can compensate for a lack of FABP4."

Can FABP5 bind directly to PPARg and should Figure 1 be amended? If so, can the authors assess the relative binding affinity of FABP4 or FABP5 for PPARg? Alternatively, is the ligand binding specificity of FABP4 vs FABP5 be sufficiently different to influence second phase differentiation?
Please see text included above discussing that FABP5 does not bind to PPARG and has been shown to increase PPARG activity indirectly.

3. An alternate model would be for the authors to consider that in vivo, there exists lipid ligands that either activate or inhibit PPARg. If so, then the function of FABP4 may be to remove an inhibitory ligand (via direct physical interaction) rather than deliver or stabilize a positive ligand. Would this model be consistent with the data in the present paper and could this model be an explanation for why PPARg activity is markedly upregulated in cells lacking FABP4?
The reviewer raises an interesting point. We believe that such a removal of an inhibitory lipid moiety is less likely since addition of linoleic acid increases PPARG activity (Fig. 5A). To explain this in the context of a putative inhibitory mechanism, one would have to propose that such a persistently bound inhibitory lipid moiety is replaced by linolic acid. Also, FABP4 is known to bind linolic acid [1], which makes it likely that the effect of linolic acid addition on PPARG activity can be explained by FABP4 delivering linoleic acid to PPARG. Furthermore, we are not aware of a known inhibitory lipid moiety binding to PPARG.

4. Still a third model might be for FABP4 to not affect ligand delivery to PPARg but to bind holo-PPARg stabilizing and extending ligand occupancy independently of any lipid shuttle function. As such, the authors should consider their discussion and consider that their data may support alternate models.
The reviewer raises again an interesting alternative model. We did not address this model directly but tested a related possibility whether the stability of PPARG is altered by binding FABP4. To address this, we performed in Fig. 6D a PPARG degradation analysis and showed there that the stability of PPARG remains the same whether or not FABP4 is delivering ligand. We believe that resolving the detailed mechanism how fatty acid gets delivered to PPARG will ultimately require structural data of FABP4 and PPARG that we will work on in the future, but we feel that such experiments are beyond the scope of our paper which focuses on the dynamics of the differentiation process.

Reviewer 2:
In their manuscript, Early enforcement of cell identity by a functional component of the terminally differentiated state, Bahrami-Nejad et al. have studied the feedback mechanisms during adipogenesis. They propose as a novel concept that positive feedback to PPARG (the necessary and sufficient adipogenic factor) includes also FABP4-mediated lipid (ligand) transport - and that this fat cell-specific functional protein thus ensures that cells reach and remain in the adipocyte attractor state.

The authors have highlighted three main findings:

1. "Fatty-acid loaded FABP4 binds to and activates PPARG": this has been shown previously. For example, the study by Tan et al 2002 (doi: 10.1128/MCB.22.14.5114-5127.2002) addressed this question and also showed that delivering the ligand to a related nuclear receptor PPARdelta promoted keratinocyte differentiation.
However, the data provided in this study does resolve conflicting results regarding the importance of FABP4 in adipogenesis and demonstrated further evidence that it provides activating ligands by interacting with PPARgamma. Furthermore, the use of rosiglitazone is shown to mask this key role (resolving conflict in previous work) and also the authors demonstrate that FABP4 mutant forms that cannot bind fatty acids act in a dominant negative manner. Due to the key role of PPARgamma in obesity and insulin resistance, the findings are of interest to a wide biomedical audience. However, it would be important to re-phrase this highlight to focus on the new insight gained.
Thank you for the positive comments on our findings. We have now re-phrased the highlights as well as the discussion to focus on the new insights gained.

In addition, I have the following comments regarding data presented:

1a: Is the labelling of Fig. S2 B panel with BODIBY staining wrong? Control cells have high BODIBY signal compared to sgFABP4 cells. This is opposite to bar plot data shown in Fig. 1I.
We thank the reviewer for catching this error on our part. The labels are indeed inverted, and we have now corrected this (please note: Fig. S2B is now Fig. S2C).

1b: What is the relative level of FABP4 vs 5 in preadipocytes in the cellular model studies vs in vivo mouse tissue? Are both upregulated during differentiation?
Yes, they are both upregulated but FABP4 is typically upregulated to >100-fold higher levels than FABP5.

The authors claim that FABP4 is needed for adipogenesis in vitro and in vivo is partially misleading in context of data presented in Fig. 1 that shows that FABP5 can compensate in part the loss of FABP4. Further data / re-phrasing would clarify this aspect to the reader.
Please see our response to Reviewer 1, point 2, since Reviewer 1 asked a similar question.

1c: As third model, the authors use the mesenchymal progenitor C3H10T1/2 cells that typically require a demethylating agent to differentiate to adipocytes in addition to DMI. Currently, only data without stimulation + FABP4 overexpression is shown and compared to with DMI stimulation + FABP4 overexpression (minimal differentiation without FABP4 overexpression). The increase upon overexpression, however, is dramatic. What is the % of cells differentiated with the previously established adipogenic cocktail - is it comparable to what is achieved by overexpression?
The reviewer makes a good point that many studies of C3H10T1/2 cell differentiation use methylation inhibitors to promote adipogenic over bone differentiation. However, there are other established

protocols to direct C3H10T1/2 cells towards adipogenesis that use DMI alone or DMI with added rosiglitazone [10,11]. In our experiments with C3H10T1/2 cells, we found that DMI with added rosiglitazone triggers nearly 100% of the cells undergo adipogenesis, but DMI triggers robust but incomplete adipocyte differentiation. We thus used the DMI protocol since it is an established C3H10T1/2 protocol that also allows us to perform the experiments under conditions where we are still able to measure increases in % of cells differentiated.

Is the basal level or protein stability of FABP4 much lower in these cells compared to the more committed cellular models? (the protein data in Fig. 1I shows arbitrary units, not possible to compare to Western blots shown in other cell lines). These additional data would contribute to a better resolved view of how the "potential" of cells to assume the terminal adipocyte cell fate differs (role of sufficient FABP4) with respect to their commitment stage.
We thank the reviewer for this comment. First, we have indeed carried overexpression of FABP4 experiments in two different progenitor cell models in this paper: C3H10T1/2 (Fig. 1I) and OP9 cells (Fig. 5B). The OP9 cells are much more committed than the CH310T1/2 cells and have much higher levels of FABP4 at basal conditions. In both cell types, overexpression of FABP4 greatly enhances adipogenesis.

We also realize there may have been some confusion in our original Fig. 1I in that there appeared to be only a two-fold difference in FABP4 expression in the overexpression experiments between DMI-treated control and FABP4-OX cells. This is likely because the FABP4 antibody used for immunocytochemistry has a lot of background. To circumvent this antibody issue and to support that the FABP4 levels are really low in C3H10T1/2 cells in control DMI-stimulated conditions and increase >100 in FABP4-OX DMI-stimulated conditions, we have replaced the panel in Fig. 1I with a FABP4 mRNA plot.

1d: Related to time course results shown in Figure 6. Was there some specific reason why the authors used siRNA instead of the FABP4 KO cells in these experiments?
We saw similar defects in differentiation when FABP4 expression was reduced using siRNA or knocked out using CRISPR-mediated genome editing. However, we used siRNA to reduce FABP4 expression in the experiment in Fig. 6C since this particular live-cell timecourse experiment made use of our OP9 cell line in which endogenous PPARG is tagged with citrine(YFP). By using siRNA, we could use the same citrine(YFP)-PPARG cell line and carry out different FABP4 and FABP5 depletion experiments in a reasonable time frame compared to generating CRISPR-KO cells for each condition.

What is the knockdown efficiency of FABP4 in OP9 cells; when does it initiate and how long does it persist? The authors have previously shown that FABP4 protein has a long half-life, therefore this control would be important. It is also relevant to understand this the data with respect to what is shown in Fig. 6A. Also state the time point of cell collection for panel A in the legend.
These were dual tagged PPARG/FABP4 cells, so we know the levels of FABP4 throughout the experiment. KD is near complete and lasts for the whole timecourse of 4 days. We are now including this FABP4 KD data as Supp. Fig. 8. We also added text in the legend (now Fig. 6C) to refer to this new data: “See Figure S8 for FABP4 knockdown efficiency and persistence.”

2. "FABP4 critically controls the second phase of adipogenesis between activation of the feedback loops and reaching the threshold to differentiate": The authors have performed experiments in several cellular models and using several complementary assays to demonstrate this, in part building upon their own previous work (Science 2014, Cell Metab 2019). There are specific experimental details and conclusions that I would ask the authors to clarify (however, I have commented on these below, in context of 3rd

main point in highlights). Overall, I find that the study provides convincing data to clarify the role of FABP4 in adipogenesis.

However, the authors propose that this model system exemplifies a more broadly relevant concept : their hypothesized model of cell differentiation, presented in the introduction, posits that cells potentially navigate through multiple alternative intermediate states, and that this path to fully differentiated state could be affected by FABP4-mediated feedback and control mechanism. This concept of "Early enforcement of cell identity additional regulatory mechanisms that selectively drive cells onto a unique path and allow cells to robustly assume and maintain a specific differentiated cell identity" that is highlighted in the title and introduction, is conceptionally novel and of broad interest. However, this aspect would require more evidence linking the presence of FABP4 (interacting with PPARgamma) and the claimed more directed differentiation path that could act as a control mechanism in context of cell state heterogeneity. Currently, the authors present data on cells tracked using live cell imaging where cells either succeed or fail to differentiate, and I find this insufficient to support their proposed control step.
We appreciate the thoughtful points made by the reviewer here. These are exactly the types of question we will be pursuing in challenging experiments using more molecular live-cell markers for alternate fates and by combining live-cell imaging with single-cell RNAseq analysis. We are currently setting up equipment, developing tools and planning such experiments but we will only be able to get results from this new line of experiment in future studies when these different technologies will be in place.

2a. In the differentiation models used, are such cell states that fail to assume the differentiated state representing cells navigating along different paths? If so, are a significant fraction of cells stuck in an intermediate state or have some cells navigated towards an alternative cell fate? For example, the mesenchymal model could be more prone to initiate alternative cell fate programs.
This is again a very insightful comment by the reviewer, and this is a direction we are planning to pursue in future work -particularly on understanding the branch-points between adipogenesis and bone differentiation, measuring if there are defined transition states on the way to the two differentiation outcomes. In our current analysis, the differentiation path in all three cell models was strongly biased towards adipogenesis. Under these conditions, we already see in some cells with fluctuating PPARG and FABP4 levels and a minority of cells that reverse back to very low PPARG and FABP4 levels. We believe to gain a deeper understanding of such path choices and reversal, we will need a combination of live-cell imaging and single cell RNAseq analysis, and we will only be able to do this with new technologies in place to mark and separate cells on specific differentiation paths.

2b. Does the fraction of such missteps (if they exist) decrease upon ligand delivery via FABP?

These questions could be addressed by analysis of global gene expression patterns as a proxy of cell state. For example, the authors could follow specific adipogenic marker genes activated at consecutive time steps, and include in the analysis also markers of alternative cell fates. This could be achieved using a targeted approach such as RNA fish/RNA flow cytometry or single cell qPCR (the first two would also allow concomitant measurement of protein expression), or in a more unbiased manner through single cell RNA-sequencing along a time series design. Current data on when each feedback loop activates could inform the selection of time points for such analysis. The targeted approach could also be designed based on protein labeling using again markers with known differentiation dynamics in adipogenesis vs alternative cell fates. This characterization could also be focused by first sorting the PPARG low vs high cell fractions at an intermediate differentiation time point,

with the assumption that cells crossing the critical level would differentiate while those not would fail, as the authors have established. Since commercial kits and establish protocols exist for such experiments, the authors should be able to perform these to support their mechanistic model.
These are again great suggestions, and we are very interested to pursue these strategies. We like the idea of using RNA fish and flow cytometry, but both will not be able to help us understand how the variable history that individual cells have define the current cell state. We believe that to address this question of differences in cell states, we will need a better way to combine our long-term live cell imaging of multiple markers with single cell RNAseq analysis. We hope to be able in due course perform such an analysis.

2c. The discussion focuses on the adipogenic model. However, a broader concept is put forward in the paper that the authors could revisit here to substantiate their propose control mechanism. For example, which other differentiation processes (cell fates) they know of that potentially employ a similar mechanism?
The reviewer raises a good question, and we recently were made aware of an example in C. elegans of a terminal selector region in a promoter that controls the cell identity of two neurons, Che-1, in a potentially similar way. Olivier Hobert's paper proposes that this selector region has two roles, to initiate the program and to then maintain the differentiated state by an autocatalytic maintenance mechanism[12]. We have added this to the discussion.
(Lines 414-416): "Interestingly, in the ASE neurons. which are important for chemotaxis in C. elegans, a critical factor for maintaining the differentiated state was found to also initiate the differentiation program, supporting that the mechanism we identified could be more universal [12]. "

Are FABPs present ubiquitous or evolved when fat accumulation in specific cells occurs in evolution?
FABP genes are present in C. elegans but seem to be missing in a number of invertebrates and PPARG appears to be restricted to vertebrate with more distant homologs in invertebrate. The most distant evolutionary evidence for a role of FABP4 and PPARG in the differentiation of adipocytes is to our knowledge in zebrafish. We would also like to note that FABP4 is also frequently abnormally highly expressed in many types of cancer cells.

2d. Along the same lines, the authors should also discuss to what extent this proposed control mechanism could apply to other transcription factors, and could it exist beyond nuclear receptors that represent classical ligand-activated transcription factors. Nuclear receptors also differ in their mechanism of activation - some of them reside in the cytoplasm and only translocate to the nucleus upon ligand binding, while others (including PPARs) have basal activity, and some have no ligand. Example among nuclear receptors that could potential fit under their proposed model would be RARs (retinol binding proteins exist) and are important in blood and skin differentiation (the role of retinoid binding proteins in RAR activation is reviewed in Fisher and Vorhees 1996, https://doi.org/10.1096/fasebj.10.9.8801161).
Yes, we think that this control mechanisms may in a modified form also apply to other transcription factors in other differentiation contexts to direct cells to a specific cell fate from an intermediate timepoint in differentiation. As the reviewer points out, such trigger and maintenance mechanisms may involve regulated nuclear localization. We don't know, however, of any specific examples at this point of an analogous mechanism where a highly expressed protein in the differentiated state is in the same way involved in directing the differentiation path. Our thought is that once a mechanism of this type is identified, there will likely be additional examples identified in other systems, and we are interested in our lab to find similar mechanisms in other differentiation systems as well.

3. "Only a small fraction (~10%) of the FABP4 levels typically attained in mature fat cells is needed to commit cells to the differentiated state, thus providing an explanation for why maintenance of the mature adipocyte state is so robust". This aspect is less clear.
We now make it clearer what we meant. We have now added the following text to the Discussion section (Lines 400-405): "FABP4 has many essential functions in mature adipocytes, and thus FABP4 would not be a good control signal if a large proportion of it was needed also as a feedback signal. FABP4 and PPARG levels can vary in differentiated cells, but since only a small fraction of the maximal FABP4 levels (<10%) are needed to feedback to PPARG, the differentiated state can be robustly maintained even when there is a 90% drop in FAPB4 level in cells. Such a robust control mechanism is likely useful for cells in vivo to keep cells differentiated once they commit to the terminally differentiated state. "

3a. The authors highlight a small initial increase in FABP4 in the single cell tracking, prior to its robust increase together with PPARg. Is the early increase in FABP protein levels necessary? Isn't the key the delivery of ligand to PPARgamma (can be substituted by rosiglitazone)?
The reviewer brings up a good point that we need to better clarify in the text. Yes, the experiments in Fig. 6B and 6C, as well as previous work by others [8,13], support that FABP4 delivers ligand to PPARG to activate it. As shown by the siRNA experiments in the current manuscript and in our previous manuscript [14], without the early increase in FABP4, PPARG levels cannot rise to the threshold for a cell to differentiate, due to lack of lipid-transfer from FABP4. The small molecule rosiglitazone can substitute for FABP4 delivery of ligand to PPARG since rosiglitazone in DMSO can pass through membranes, reach PPARG in the nucleus, and bind to the PPARG ligand domain without needing to be transported through the cytosol.

Presently, there is no mention/data regarding i) FABP sub-cellular localization during early adipogenesis or ii) availability of fatty acid ligands inside the cells through transport and possible changes in these upon DMI stimulus. Insulin (added to cells in DMI) can promote fatty acid transport to cells (Dev Cell. 2002 doi: 10.1016/s1534-5807(02)00143-0) and affect FABP cellular localization. Quantifying the nuclear vs cytoplasmic vs membrane localization of this critical small FABP4 pool in preadipocytes, and potential changes upon the first days upon DMI would be very relevant to substantiate the observation that early increase (missed in previous bulk studies) indeed is a key control step.
This data could potentially be obtained from existing results, in case sufficient resolution to distinguish cellular compartment signals is available.
This is again a good point that we have also considered. We have not observed a difference in FABP4 going up in the nucleus versus cytoplasm in response to either rosiglitazone, linoleic acid, or DMI addition in our many experiments.

3b. In the present manuscript, the authors make the distinction that FABP4 and PPARgamma protein levels increase concordantly but at very different scales (log scale for FABP4 used as it increases much more). The authors have previously shown (Cell Metab 2019) that FABP4 degradation is low compared to e.g. PPARgamma, and relevant in filtering circadian fluctuations that could trigger spurious differentiation. However, no mention of relative stabilities is included in the interpretation of this result, although this could clarify the difference observed. Moreover, this is relevant also in context of the siRNA experiments the authors used to define the order of the feedback loops. If PPARgamma and CEBPalpha stabilities are low, siRNA knockdown would likely rapidly decrease their levels in cells.

However, have the authors confirmed that FABP4 was absent upon induction of DMI in the experiments shown in Fig 4A? Or is there some residual protein left that may delay when the two curves separate?
The reviewer brings up a good point, and it is relevant to relate the regulation we see here to the lifetime of FABP4 proteins. The time for protein levels to reach steady state is dependent on the protein halflife [15], and we have previously shown that the slower increase of FABP4 relative to PPARG is a result of the much longer lifetime of FABP4 compared to PPARG [14]. As for the amount of residual FABP4 protein, the conditions we use for siRNA are in cells before we stimulate with DMI and the knockdown efficiency is very high (Please also see Fig. S8 and our response to your comment 1d). At this timepoint, the protein levels of PPARGgamma, CEBPalpha and FABP4 are very low. Specifically, the cells were treated with siRNA at Day0 before we added differentiation stimuli. At that time PPARgamma, CEBPalpha and FABP4 have not started to be upregulated. Under these conditions combined with our very high siRNA knockdown efficiency, there was very little residual amounts of all three proteins upon induction of DMI in all experiments presented.

3c. Recently, Aprile et al. (2018, Cell Reports https://doi.org/10.1016/j.celrep.2018.10.035) demonstrate, in both human and mouse adipogenesis, the existence of a dominant negative splice variants for PPARgamma. Are the authors familiar with this work? The data in their cellular models using the endogenously tagged PPARgamma would need to address this point. Which splice variants are generated in the system studied here during differentiation and how does the increase in the potential dominant negative form relate to timing of the feedbacks observed here? The abundance of the dominant negative form was shown to increase upon ligand activation. Does this occur in their models? Or could it explain some of the differences between the model systems used here such as differentiation timing?
This dominant negative form of PPARG generated by splicing is an interesting recent finding that adds another regulatory angle to the adipogenesis process. The three cell models we studied, increased expression of PPARG is associated during the critical commitment time with an increase in transcriptional activity (seen as a sigmoidal increase in PPARG levels). This suggests that at least during this critical time window, the positive role of PPARG is dominating. This is confirmed when we look at the splice variants of PPARG that are expressed when we carried out RNA-seq analysis of our OP9 cells over the 5-day timecourse of adipogenesis. The negative isoform that Aprile et al. (2018) identified lacks the ligand binding domain[16]. This isoform is not expressed in our OP9 cells. Only the 201 and 204 isoforms, which both have the ligand binding domain are the only isoforms that are significantly expressed.

What is the interaction domain in PPARgamma that binds FABPs? Is it present in the truncated dominant negative variant?
We also would like to note that the interaction domain in PPARG that binds FABPs is unknown. It is actually something we hope to uncover in a future collaboration with a crystallographer interested in this problem that may also help with understanding how this inhibitory and potentially other known spliced forms of PPARG regulate PPARG target expression.

3d. In their earlier work (Science 2014) the authors have computationally modelled the bifurcation that occurs during differentiation where increase in ligand (x-axis in bifurcation plot), needs to be pushed sufficiently high in pre-adipocytes to trigger bifurcation where PPARgamma protein level (y-axis) threshold is reached, and in this model the additional feedbacks including FABP4 ensure that the system becomes robust to noise (less de-differentiation). In minimum, the highlights statements should also mention the ligand availability, to clarify what is the mechanism relating FABP to commitment and

maintenance, and the authors should explicitly reference their previous bifurcation analysis (unless I misunderstand what is meant by R in the bifurcation plots in previous work). As the group has experience in dynamic models, connecting the data obtained here with the mathematical model (possibly slightly modified from their previous work to include the ligand transport)
could be attempted, and would allow further investigation of the proposed mechanism via additional simulations in physiologically realistic parameter ranges. Such models could also be useful to examine the role of the dominant negative PPARg in future. Moreover, providing such a model freely available to the community would allow this concept to be tested in other differentiation models, attracting the attention of a wider audience of both experimental and theoretical biologists.

This is again an important point that we would very much like to address in future papers. We would like to wait to generate a more comprehensive model since it would need to include an analysis of single-cell RNAseq data for the same process in order to map the live-cell data to potential intermediate states along the differentiation path.

Reviewer. 3: Christian Schröter – note that this reviewer has waived anonymity
Bahrami-Nejad et al. report a new positive feedback loop between FABP4 and PPARG during adipogenic differentiation. Using CRISPR/Cas9 mutagenesis and siRNA knock-down, they show that FABP4 is required for the efficient adipogenic differentiation of several model cell lines. They demonstrate lipid-dependent binding of wild type FABP4 to PPARG, suggesting that FABP functions to enhance PPARG transcriptional activity by transferring lipid ligands. To dissect the temporal relationship between FABP4- and PPARG-expression, they perform single-cell imaging of new reporter cell lines.

The paper reports a comprehensive set of experiments ranging from the molecular to the phenotypic level which are all well carried out. Together, they lead the authors to propose that a protein that has so far been implicated in the metabolism of terminally differentiated cells takes on an additional role in a regulatory network to attract cells to a specific differentiated state. This is a new concept that is of potential relevance in other systems.
Thank you for the positive comments and constructive points of critique.

While the paper is overall well written, I had some problems following the author's arguments regarding the temporal sequence of feedback loop engagement in their system given the single cell imaging data. Furthermore, the proposal that FABP4 and PPARG form a positive feedback loop makes predictions about the dynamical behavior of the system which the authors could test using existing reagents. This would make the manuscript considerably stronger. Before publication, I therefore recommend the following major amendments:

1. In their model in Fig. 4C, the authors suggest that two feedback loops are engaged sequentially during adipogenic differentiation, the first one involving PPARG and CEBPA, the second, later one involving PPARG and FABP4. This would predict that in single cells, PPARG expression starts to rise before FABP4 expression. This sequential rise in expression in single cells is not obvious from the data shown in Figs. 2 and 3. The binned data in Figs. 3A and B appears to indicate a slight delay in FABP4 expression relative to PPARG expression, but in the single cell traces in Fig. 2B, both seem to increase at the same time. Instead of aggregating the data, could the authors plot PPARG vs. FABP4 reporter expression in single cells (with perhaps time encoded in a third dimension)? This should show a characteristic delay in the rise of FABP4 expression in single cells, which would be expected to mirror the delay seen in the siRNA experiments shown in Fig. 4A. Additional analysis of their single-cell imaging data is essential to support the authors' conclusions regarding the sequential engagement of distinct feedback loops.
This is a good point, and the issue might be that we had originally plotted the FABP4 data in Fig. 2B with a logscale which meant the differences were not so obvious. We have now plotted the data on a linear scale and have added additional analysis to Fig. 2 to make the timing differences more obvious.

2. The authors' proposal that the positive feedback loop established by PPARG and FABP4 establishes and maintains the differentiated state makes two key predictions: First, differentiation should require a threshold concentration of FABP4 expression. Second, cells should revert if FABP4 expression is switched off. Both predictions could be tested with the doxycycline inducible lines expressing fluorescently tagged FABP4 (Fig. 5). While not strictly essential, performing these additional experiments on the dynamical behavior of the system would strengthen the author's point that FABP4 indeed is embedded in a positive feedback loop, rather than simply functioning in parallel to PPARG during adipogenic differentiation.
This is an excellent point, and we tried in initial experiments to do the dox removal experimentally. It is however difficult to use just removal of dox to lower the expression of FABP4 since it is long-lived and also really highly expressed (makes up 0.5-6% of total protein in adipocytes). Thus, we are currently

planning a follow-up paper where we explore the stability of the differentiated state using dox-auxin regulated PPARG and FABP4 (in order to rapidly and more completely degrade either protein) and plan to do this both in vitro and in a fat pad model in mice. We hope we can use these experiments to explore the stability of the differentiated state and to better understand what might be going wrong in insulin resistant/diabetic conditions when adipocytes may lose key characteristics of the differentiated state. The same experiments can then also be performed during the initial commitment process to much more rapidly downregulate PPARG and FABP4 to more precisely understand how cells use this positive feedback to transition to a terminally differentiated state.

In addition, there are a couple of minor points that should be addressed before publication:
* Fig. 1: Could the authors show primary data with PPARG immunostaining and quantification in the supplement to demonstrate the binary nature of the fate decision? I am aware that they have shown this in previous papers, but it would be nice to have it here again for internal reference.
We have now added primary data and a description of our quantification methodology as Fig. 1B,1C,1D.

* Fig. 1I: Sample identity should be indicated more clearly.
Thank you for pointing this out. We have relabeled the samples in Fig. 1l to make the sample identity clearer.

* The relationship between main text and supplementary material needs to be updated. I could not find the adiponectin mRNA data in Fig. S1 that the main text refers to, instead I find there immunostaining data that is appears to refer to the rosiglitazone experiment in the main figure 6. Figure legend to S2B is different below the figure and in the manuscript file.
We have now corrected this in the text.

* P. 6, remove reference to Fig. 2 when mentioning "Our in vitro and in vivo results…".
We have now corrected this in the text.

* Fig. 4A: Are the traces of PPARG abundance different for the different siRNA treatments?
The blue siRNA and grey control treated conditions are shown at the same scale. We have now clarified in the Methods section "The same scrambled control timecourses were used to compare against the three knockdown timecourses (siCEBPA, siCEBPA, and siFABP4) in Fig. 4A." (Lines 1131-1133).

* P. 10: The sentence "Of note, the PPARG threshold, which marks…." is confusing.
We changed the sentence and wrote a better explanation of how the threshold can be identified. "The PPARG threshold, which marks the transition between Phase 2 and Phase 3, cannot be seen just by inspecting the single-cell or averaged traces. The threshold needs to be identified either (1) by using live-cell imaging to measure PPARG levels, removing the external differentiation stimulus, and then continuing to track each individual cell to know their final differentiation state days later [14,17] or (2) by taking advantage of the binary nature of the differentiation decision and carrying out suitable analyses such as a Gaussian mixture model analysis [17] or a receiver operating characteristic (ROC) analysis in which different threshold levels are surveyed to find the one that maximizes the difference between true and false positive rates for predicting cell fate choice [18,19]." (Lines 298-305).

* Why is the differentiation efficiency reported in Fig. 5B for the control different from that reported for FABP4 KO cells in Fig. 1C? I would expect that the expression of YFP in Fig. 5 should not make such a big difference. This needs to be discussed.

The cells in the original Fig. 5B were made by transfecting in the three overexpression constructs in Fig. 5A into the cells in Fig. 1C. After this, the 3 new cell lines underwent antibiotic selection. The extra passaging and selection steps may have resulted in increased differentiation even when just YFP alone was expressed. We also noticed the difference in efficiency which we realized is the result of multiple rounds of selection of cells. We now instead replaced the data with transient expression data carried out in wildtype OP9 cells to avoid issues with using stable clones that need long selection protocols to make.

* Reference to Fig. 5 panels C and D is switched in the main text.
We have now corrected this in the text.

* In Fig. 6A, B: It would be helpful to show differentiation efficiency for same experiments in the absence of rosiglitazone for comparison.
We have now more clearly stated in the figure legend that the same experiments in the absence of rosiglitazone are shown in Fig. 1F and 1H, and representative images are presented in Supp. Figs. 1C-D and 2C-D.
"(A) Addition of Rosiglitazone (1 μM) to the differentiation stimulus (DMI) rescues the loss of adipogenesis shown in Figure 1F in FABP4-KO OP9 cells and in FABP4-KO OP9 cells transfected with FABP5 siRNA. Percent of differentiated cells was quantitated as in Figure 1D. See Figures S1C-D for representative images.
(B) The addition of Rosiglitazone (1 μM) to the differentiation media (insulin added to the growth media) rescues the loss of adipogenesis in FABP4-KO, FABP5-KO, and DBKO 3T3-F442A cells shown in Figure 1H. See Figures S2C-D for representative images."

* CEBPA: If necessary introduce abbreviation in the beginning of the manuscript, rather than only in the discussion on p. 14.
Thank you for pointing this out.

Reviewer 4:
This manuscript shows that the lipid transporterPARP4 plays a central role in shifting the balance towards adipogenesis differentiation, by directly binding to PPARG and increase its activity. This is a novel concept, that a differentiation marker is directly involved in the lineage decision within the progenitor stage.

Crispr induced knockout of FABP4 were introduced in both, OP9 cells as well as 3T3-F442A cells and resulted in reduced fatty acid differentiation. Overexpression of FABP4 in C3H10T1/2 mesenchymal cells by Crispr led to adipogenesis and robust PPARG expression and lipid accumulation upon an input stimulus. Results from time lapse imaging showed increase of CEBPA, FABP4 and PPARG levels in co-dependent manner . These results were interpreted as independent feedback loops - one early CEBPA-PPARG and a later FABP4-PPARG that increase PPARG in early- and intermediate states of differentiation. Co-immunoprecipitation confirmed the interaction of FABP4 with PPARG in the presence of fatty acids. To test their hypothesis, and a non-fatty acid binding mutant form of FABP4 as well as the small molecule Rosiglitazone which binds to a pocket of PPARG normally activated by fatty acid, were used.

I appreciate the overall quality of this study which will be interesting for many researchers. Lots of efforts were taken by using Crispr/Cas9 to genetically modify and generate knock out or reporter cell lines. The phenotype of knockout cells was further examined with time lapse study and a thorough quantification. These descriptive results were further supported by proving direct interaction of PARAP4 and PPARG using pull down experiment.
Thank you for the overall very positive comments.

However, there were a few points that should be addressed or clarified:

1. - In contrast to the clones generated for Crispr induced activation of PARP4, where sequencing and Southern blot were shown for quality controls (QC), not much was mentioned about the QC controls in Crispr induced FABP4 and FABP55 KO cells as well as in the double KOs. Were these full KOs or frame shift mutants with a residual truncated protein. Where was the gRNA binding site located within the gene? Where was the epitope binding of the antibody used for the KO- detection located? Would the antibody pick up a truncated protein? Could you provide sequencing data on both alleles of the KO cell lines, to determine the exact nature of the Crispr induced mutation and check highest specific off target sites?
We have now included this data as part of Figs. S1 and S2.

How many mutant clones were used for this study or how to did the authors exclude clonal variation?
We agree that this is a relevant control. We now include data showing we see the same effects with a second clone (Fig. S3). We also used siRNA knockdown of FABP4 into wildtype cells, combined with rescue of the defective differentiation by overexpression of siRNA-resistant FABP4, in Fig. 5 as an orthogonal way to validate the FABP4 CRISPR-KO clone results.

2. Live cell imaging experiments in CEBPA or FABP4 knockdowns were interpreted in such way, that CEBPA and PPARG defined an early feedback loop whereas the FABP4 and PPARG a later feedback loop during adipocyte differentiation. However, the authors remain unclear how these feedback loops are different in PPARG activation, as the CEBPA and PPARG feedback fails to lift PPARG above a certain threshold level.

The reviewer brings up a good point that we have been discussing as well. Our interpretation is that PPARG activity is significantly lower at the same level of PPARG when FABP4 is absent. We believe this effectively mimics a few-fold down-regulation of PPARG compared to conditions where PPARG can more readily be activated by fatty acids. For this reason, even though the feedback between PPARG and CEBPA may start to engage, it does not engage strongly enough to start self-amplification and the explosive increase in PPARG. In this way, FABP4 can be considered to be a sensitizing factor that increases PPARG activity above the threshold where PPARG and CEBPA can more fully amplify each other.

3. In Figure 4A PPARG abundance also increased in FABP4 knockdown cells, but at a much slower rate. Is the adipose differentiation in FABP4 KO cells hampered or only slowed down? Would differentiation efficiencies improve in KO cells when given longer times for differentiation?
We think both are happening at the same time. With PPARG being less active without FABP4, its level rises much slower due a lack of the normally engaging CEBPA- and FABP4-mediated positive feedback. We see that the outcome at the level of the cell population is two-fold, a few cells may ultimately increase PPARG high enough to differentiate while others remain undifferentiated even for the long term.

4 and 5. What remains puzzling is that fatty acid binding of wt FABP4 is crucial for PPARG interaction, whereas the non-fatty acid binding mutant FABP4-R126Q interacts with PPARG in all conditions. Wouldn´t one expect that the mutant isoform of FABP4 could not bind to PPARG at all? While the authors could show that binding of FABP4 to PPARG does not have any effect on PPARG stability, they failed to show that this interaction yielded in higher activity of PPARG. As this is a central claim of the paper, I would appreciate more mechanistic data supporting their hypothesis. Maybe in vitro promotor studies could clarify this point.

The reviewer raises good points about what can be learned from the mutant FABP4 data that we previously included. It is well known that fatty acid binding is needed to activate PPARG[20], and other groups have shown that fatty acid binding of wt FABP4 is crucial for PPARG activation [8,13,21]. We agree with the reviewer that the mutant data that we can generate in cells does not resolve the regulatory mechanism. To definitively resolve the detailed mechanism how fatty acid gets delivered to PPARG will ultimately require in vitro biochemical and structural analysis of FABP4 and PPARG that we will work on in the future. Such a detailed molecular in vitro analysis of how FABP4 mediates lipid transport is beyond the scope of our study here, which instead focuses on the regulatory question how FABP4 contributes to the decision whether to stay in a preadipocyte state or undergo terminal differentiation and become an adipocyte. Instead of adding such a challenging biochemical in vitro analysis to address points outside the main novelty of the paper, we have now deemphasized this part and clarified in the Introduction and Discussion that the study focuses on the cellular mechanism of differentiation and that future structural and that reconstitution analysis is needed to conclusively resolve the detailed fatty acid transfer and activation mechanisms.

6. The model of FABP4 function in the terminally differentiated state (Fig7) does not look very convincing and could include the point mutation of FABP4, the fatty acids or illustrate better the different states of commitment of the progenitor cells.
Thank you for pointing this out. We now make our point clearer in the model figure.

REFERENCES

1. Boss M, Kemmerer M, Brune B, Namgaladze D. FABP4 inhibition suppresses PPARgamma activity and VLDL-induced foam cell formation in IL-4-polarized human macrophages. Atherosclerosis. 2015;240: 424–430. doi:10.1016/j.atherosclerosis.2015.03.042

2. Lamas Bervejillo M, Bonanata J, Franchini GR, Richeri A, Marqués JM, Freeman BA, et al. A FABP4-PPARγ signaling axis regulates human monocyte responses to electrophilic fatty acid nitroalkenes. Redox Biology. 2020;29: 101376. doi:10.1016/j.redox.2019.101376

3. Furuhashi M, Saitoh S, Shimamoto K, Miura T. Fatty Acid-Binding Protein 4 (FABP4): Pathophysiological Insights and Potent Clinical Biomarker of Metabolic and Cardiovascular Diseases. Clinical Medicine Insights Cardiology. 2014;8: 23–33. doi:10.4137/CMC.S17067.RECEIvED

4. Coe NR, Simpson MAMA, Bernlohr DA. Targeted disruption of the adipocyte lipid-binding protein (aP2 protein) gene impairs fat cell lipolysis and increases cellular fatty acid levels. Journal of Lipid Research. 1999;40: 967–972.

5. Hotamisligil GS, Johnson RS, Distel RJ, Ellis R, Papaioannou VE, Spiegelman BM. Uncoupling of Obesity from Insulin Resistance Through a Targeted Mutation in aP2, the Adipocyte Fatty Acid Binding Protein. Science (New York, NY). 1996;274: 1377–1379.

6. Shaughnessy S, Smith ER, Kodukula S, Storch J, Fried SK. Adipocyte metabolism in adipocyte fatty acid binding protein knockout mice (aP2-/-) after short-term high-fat feeding: functional compensation by the keratinocyte [correction of keritinocyte] fatty acid binding protein. Diabetes. 2000;49: 904–11. doi:10.2337/diabetes.49.6.904

7. Matsusue K, Peters JM, Gonzalez FJ. PPARβ/δ potentiates PPARγ-stimulated adipocyte differentiation. FASEB j. 2004;18: 1477–1479. doi:10.1096/fj.04-1944fje

8. Tan N-S, Shaw NS, Vinckenbosch N, Liu P, Yasmin R, Desvergne B, et al. Selective Cooperation between Fatty Acid Binding Proteins and Peroxisome Proliferator-Activated Receptors in Regulating Transcription. MOL CELL BIOL. 2002;22: 15.

9. Djian P, Phillips M, Green H. The activation of specific gene transcription in the adipose conversion of 3T3 cells. Journal of Cellular Physiology. 1985;124: 554–556. doi:10.1002/jcp.1041240327

10. Rong JX, Klein JLD, Qiu Y, Xie M, Johnson JH, Waters KM, et al. Rosiglitazone induces mitochondrial biogenesis in differentiated murine 3T3-L1 and C3H/10T1/2 adipocytes. PPAR Research. 2011;2011. doi:10.1155/2011/179454

11. Cho YC, Jefcoate CR. PPARγ1 synthesis and adipogenesis in C3H10T1/2 cells depends on S-phase progression, but does not require mitotic clonal expansion. Journal of Cellular Biochemistry. 2004;91: 336–353. doi:10.1002/jcb.10743

12. Leyva-Díaz E, Hobert O. Transcription factor autoregulation is required for acquisition and maintenance of neuronal identity. Development. 2019;146: dev177378. doi:10.1242/dev.177378

13. Ayers SD, Nedrow KL, Gillilan RE, Noy N. Continuous Nucleocytoplasmic Shuttling Underlies Transcriptional Activation of PPARγ by FABP4. Biochemistry. 2007;46: 6744–6752. doi:10.1021/bi700047a

14. Bahrami-Nejad Z, Zhao ML, Tholen S, Hunerdosse D, Tkach KE, van Schie S, et al. A Transcriptional Circuit Filters Oscillating Circadian Hormonal Inputs to Regulate Fat Cell Differentiation. Cell Metabolism. 2018;27: 854-868.e8. doi:10.1016/j.cmet.2018.03.012

15. Rosenfeld N, Elowitz MB, Alon U. Negative Autoregulation Speeds the Response Times of Transcription Networks. Journal of Molecular Biology. 2002;323: 785–793. doi:10.1016/S0022-2836(02)00994-4

16. Aprile M, Cataldi S, Ambrosio MR, D'Esposito V, Lim K, Dietrich A, et al. PPARγΔ5, a Naturally Occurring Dominant-Negative Splice Isoform, Impairs PPARγ Function and Adipocyte Differentiation. Cell Reports. 2018;25: 1577-1592.e6. doi:10.1016/j.celrep.2018.10.035

17. Zhao ML, Rabiee A, Kovary KM, Bahrami-Nejad Z, Taylor B, Teruel MN. Molecular Competition in G1 Controls When Cells Simultaneously Commit to Terminally Differentiate and Exit the Cell Cycle. Cell Reports. 2020;31: 107769. doi:10.1016/j.celrep.2020.107769

18. Schröter C, Rué P, Mackenzie JP, Arias AM. FGF/MAPK signaling sets the switching threshold of a bistable circuit controlling cell fate decisions in ES cells. Development. 2015; dev.127530. doi:10.1242/dev.127530

19. Zhang Z-B, Sinha J, Bahrami-Nejad Z, Teruel MN. The circadian clock mediates daily bursts of cell differentiation by periodically restricting cell differentiation commitment. Proceedings of the National Academy of Sciences. 2022;in press. doi:10.1101/2022.05.17.492365

20. Tontonoz P, Hu E, Spiegelman BM. Stimulation of adipogenesis in fibroblasts by PPAR gamma 2, a lipid-activated transcription factor. Cell. 1994;79: 1147–1156. doi:10.1016/0092-8674(94)90006-x

21. Adida A, Spener F. Adipocyte-type fatty acid-binding protein as inter-compartmental shuttle for peroxisome proliferator activated receptor γ agonists in cultured cell. Biochimica et Biophysica Acta (BBA) - Molecular and Cell Biology of Lipids. 2006;1761: 172–181. doi:10.1016/j.bbalip.2006.02.006
