# Supplementary material for: Early enforcement of cell identity by a functional component of the terminally differentiated state
Source: PLoS Biol. 2022 Dec 5;20(12):e3001900. doi: 10.1371/journal.pbio.3001900 (PMC9721491; doi:10.1371/journal.pbio.3001900)
Supplement: Peer review history

# Author comment: Author response to previous submission

**DOI:** 10.1371/journal.pbio.3001900.r001

8 Aug 2022

Attachment Submitted filename: 2022-08-08-response-to-reviewers.pdf Click here for additional data file.

# Editor report: Decision Letter 0

**DOI:** 10.1371/journal.pbio.3001900.r002

**Authors:** Ines Alvarez-Garcia

20 Aug 2022

Dear Dr Teruel, 

Thank you for submitting the revised versio of your manuscript entitled "­Early enforcement of cell identity by a functional component of the terminally differentiated state" for consideration as a Research Article by PLOS Biology.

Your manuscript has now been evaluated by the PLOS Biology editorial staff as well as by the original academic editor and I am writing to let you know that we are going to send your submission back to the original reviewers. However, as this is a new submission, we need you to complete again your submission by providing the metadata that is required for peer review. To this end, please login to Editorial Manager where you will find the paper in the 'Submissions Needing Revisions' folder on your homepage. Please click 'Revise Submission' from the Action Links and complete all additional questions in the submission questionnaire.

Once your full submission is complete, your paper will undergo a series of checks in preparation for peer review. After your manuscript has passed the checks it will be sent out for review. To provide the metadata for your submission, please Login to Editorial Manager (https://www.editorialmanager.com/pbiology) within two working days, i.e. by Aug 23 2022 11:59PM.

Feel free to email us at plosbiology@plos.org if you have any queries relating to your submission.

Kind regards,

Ines

--

Ines Alvarez-Garcia, PhD

Senior Editor

PLOS Biology

ialvarez-garcia@plos.org

# Author comment: Author response to Decision Letter 0

**DOI:** 10.1371/journal.pbio.3001900.r003

23 Aug 2022

Attachment Submitted filename: 2022-08-20-response-to-reviewers.pdf Click here for additional data file.

# Peer review: Decision Letter 1

**DOI:** 10.1371/journal.pbio.3001900.r004

**Authors:** Ines Alvarez-Garcia

28 Sep 2022

Dear Mary,

Thank you for your patience while we considered your revised manuscript entitled "­Early enforcement of cell identity by a functional component of the terminally differentiated state" for publication as a Research Article at PLOS Biology. This revised version of your manuscript has been evaluated by the PLOS Biology editors, the Academic Editor and three of the original reviewers.

Based on the reviews (attached below), we are likely to accept this manuscript for publication, provided you satisfactorily address the remaining minor points raised by Reviewer 3. Please also make sure to address the data and other policy-related requests stated below.

In addition, we would like you to consider a suggestion to improve the title:

"A functional component of the terminally differentiated state also induces early enforcement of cell identity in progenitor cells"

As you address these items, please take this last chance to review your reference list to ensure that it is complete and correct. If you have cited papers that have been retracted, please include the rationale for doing so in the manuscript text, or remove these references and replace them with relevant current references. Any changes to the reference list should be mentioned in the cover letter that accompanies your revised manuscript.

We expect to receive your revised manuscript within two weeks. 

To submit your revision, please go to https://www.editorialmanager.com/pbiology/ and log in as an Author. Click the link labelled 'Submissions Needing Revision' to find your submission record. Your revised submission must include the following:

- a cover letter that should detail your responses to any editorial requests, if applicable, and whether changes have been made to the reference list

- a Response to Reviewers file that provides a detailed response to the reviewers' comments (if applicable)

- a track-changes file indicating any changes that you have made to the manuscript. 

NOTE: If Supporting Information files are included with your article, note that these are not copyedited and will be published as they are submitted. Please ensure that these files are legible and of high quality (at least 300 dpi) in an easily accessible file format. For this reason, please be aware that any references listed in an SI file will not be indexed. For more information, see our Supporting Information guidelines:

https://journals.plos.org/plosbiology/s/supporting-information

*Published Peer Review History*

Please note that you may have the opportunity to make the peer review history publicly available. The record will include editor decision letters (with reviews) and your responses to reviewer comments. If eligible, we will contact you to opt in or out. Please see here for more details:

https://blogs.plos.org/plos/2019/05/plos-journals-now-open-for-published-peer-review/

*Press*

Should you, your institution's press office or the journal office choose to press release your paper, please ensure you have opted out of Early Article Posting on the submission form. We ask that you notify us as soon as possible if you or your institution is planning to press release the article.

*Protocols deposition*

To enhance the reproducibility of your results, we recommend that if applicable you deposit your laboratory protocols in protocols.io, where a protocol can be assigned its own identifier (DOI) such that it can be cited independently in the future. Additionally, PLOS ONE offers an option for publishing peer-reviewed Lab Protocol articles, which describe protocols hosted on protocols.io. Read more information on sharing protocols at https://plos.org/protocols?utm_medium=editorial-email&utm_source=authorletters&utm_campaign=protocols

Please do not hesitate to contact me should you have any questions.

Sincerely,

Ines

--

Ines Alvarez-Garcia, PhD

Senior Editor

PLOS Biology

ialvarez-garcia@plos.org

------------------------------------------------------------------------

ETHICS STATEMENT:

Thank you for providing the ethics statement. Please include the license/approval number.

------------------------------------------------------------------------

DATA POLICY: IMPORTANT - PLEASE READ

You may be aware of the PLOS Data Policy, which requires that all data be made available without restriction: http://journals.plos.org/plosbiology/s/data-availability. For more information, please also see this editorial: http://dx.doi.org/10.1371/journal.pbio.1001797

Note that we do not require all raw data. Rather, we ask that all individual quantitative observations that underlie the data summarized in the figures and results of your paper be made available in one of the following forms:

1) Supplementary files (e.g., excel). Please ensure that all data files are uploaded as 'Supporting Information' and are invariably referred to (in the manuscript, figure legends, and the Description field when uploading your files) using the following format verbatim: S1 Data, S2 Data, etc. Multiple panels of a single or even several figures can be included as multiple sheets in one excel file that is saved using exactly the following convention: S1_Data.xlsx (using an underscore).

2) Deposition in a publicly available repository. Please also provide the accession code or a reviewer link so that we may view your data before publication. 

Regardless of the method selected, please ensure that you provide the individual numerical values that underlie the summary data displayed in the following figure panels as they are essential for readers to assess your analysis and to reproduce it:

Fig. 1D, F, H, K-M; Fig. 2B-E; Fig. 3A-C, E, F; Fig. 4A-D; Fig. 5B, C; Fig. 6A-D; Fig. S1B, C; Fig. S3C, D; S4A; Fig. S6F; Fig. S7 and Fig. S8

NOTE: the numerical data provided should include all replicates AND the way in which the plotted mean and errors were derived (it should not present only the mean/average values).

Please also ensure that figure legends in your manuscript include information on WHERE THE UNDERLYING DATA CAN BE FOUND, and ensure your supplemental data file/s has a legend.

Please ensure that your Data Statement in the submission system accurately describes where your data can be found.

**In addition, please make sure that the data you have deposited in the Zenodo database (accession number: 10.5281/zenodo.7012787) is made publicly available at this stage.

------------------------------------------------------------------------

SPECIES INDICATED IN THE ABSTRACT

- Please note that per journal policy, the model system/species studied should be clearly stated in the abstract of your manuscript. 

-------------------------------------------------------------------------

BLOT AND GEL REPORTING REQUIREMENTS:

We require the original, uncropped and minimally adjusted images supporting all blot and gel results reported in an article's figures or Supporting Information files. We will require these files before a manuscript can be accepted so please prepare and upload them now. Please carefully read our guidelines for how to prepare and upload this data: https://journals.plos.org/plosbiology/s/figures#loc-blot-and-gel-reporting-requirements

------------------------------------------------------------------

Reviewers' comments

Rev. 2:

I have now had a chance to go over the revised manuscript. The authors have provided thoughtful comments to the feedback and I find that overall they addressed the key points raised during review, I have no further comments or suggestions.

Rev. 3: Christian Schröter

I was happy to finally see a revised version of Bahrami-Nejad's manuscript. As suggested in my first review, the authors have now changed the way they represent data relating to the temporal relationship between PPARG and FABP4 expression (Fig. 2). I find this new analysis is much clearer and strongly supports the author's points. They have also appropriately attended to all minor issues. Perturbation experiments to further probe the predicted dynamical behavior of the system have not been performed, in line with the previous editorial assessment that these experiments were not essential to the story. The data presented in Fig.5 have now been obtained with a transient overexpression approach, to avoid problems with cell line drift upon extended selection. I notice that, prompted by comments 4 and 5 of reviewer #4, the authors have decided to remove from Figure 5 the analysis of an FABP4 mutant version, with the motivation to focus their story more on the cellular basis of differentiation.

In response to the other reviewer's comments, the authors have performed additional analyses in FABP4/FABP5 mutant 3T23-F442A cells, and characterized their genetic Crispr-mutants more thoroughly. These new data are well-presented and make the work more solid.

I have positively commented on the value of the work before. Given that all essential points have been appropriately addressed, I fully support publication of the work in PLoS Biology.

In the final version, the authors may want to clarify the methods section on cell line tagging (lines 962 ff). In the introductory paragraph, they say that they describe tagging of FABP4 with mKate2, but paragraphs B) and C) (lines 982 ff) seem to refer to their previous work on tagging PPARG with Citrine.

Rev. 4:

This manuscript shows a novel concept of double feedback loop that drives preadipocytes to commit to terminally differentiated adipocytes involving PPARG, CEBPA and FABP4.

The authors of above-mentioned manuscript successfully addressed all comments that were previously raised.

Quality controls for Crispr induced FABP4 and FABP5 KO and double KO cells are now shown in a supplementary figure. Clonal variation of mutant cell lines was excluded by showing the phenotype in a second independent cell clone. Many parts of the manuscript are now written much better and either removed the emphasis on weak points or included better explanations. To resolve the mechanism how fatty acids get delivered to PPARG require in vitro biochemical analysis, which, the authors claim, is beyond the scope for this study. Finally the model figure at the end has been adapted, though I still think the graphical illustration and written explanation could be improved.

Taken all together, I think that this revision has improved this manuscript substantially.

# Editor report: Decision Letter 2

**DOI:** 10.1371/journal.pbio.3001900.r005

**Authors:** Ines Alvarez-Garcia

2 Nov 2022

Dear Mary,

Thank you for the submission of your revised Research Article entitled "­Early enforcement of cell identity by a functional component of the terminally differentiated state" for publication in PLOS Biology. On behalf of my colleagues and the Academic Editor, Sui Huang, I am happy to say that we can in principle accept your manuscript for publication, provided you address any remaining formatting and reporting issues. These will be detailed in an email you should receive within 2-3 business days from our colleagues in the journal operations team; no action is required from you until then. Please note that we will not be able to formally accept your manuscript and schedule it for publication until you have completed any requested changes.

Please take a minute to log into Editorial Manager at http://www.editorialmanager.com/pbiology/, click the "Update My Information" link at the top of the page, and update your user information to ensure an efficient production process.

PRESS

We frequently collaborate with press offices. If your institution or institutions have a press office, please notify them about your upcoming paper at this point, to enable them to help maximise its impact. If the press office is planning to promote your findings, we would be grateful if they could coordinate with biologypress@plos.org. If you have previously opted in to the early version process, we ask that you notify us immediately of any press plans so that we may opt out on your behalf.

We also ask that you take this opportunity to read our Embargo Policy regarding the discussion, promotion and media coverage of work that is yet to be published by PLOS. As your manuscript is not yet published, it is bound by the conditions of our Embargo Policy. Please be aware that this policy is in place both to ensure that any press coverage of your article is fully substantiated and to provide a direct link between such coverage and the published work. For full details of our Embargo Policy, please visit http://www.plos.org/about/media-inquiries/embargo-policy/.

Many congratulations and thanks again for choosing PLOS Biology for publication and supporting Open Access publishing. We look forward to publishing your study. 

Sincerely, 

Ines

--

Ines Alvarez-Garcia, PhD

Senior Editor

PLOS Biology

ialvarez-garcia@plos.org
